# *Acridocarpus smeathmannii* root extracts inhibit human prostate and bladder smooth muscle contraction, porcine arterial vasoconstriction, and cytotoxicity of prostate stromal cells

**DOI:** 10.3389/fphar.2025.1621346

**Published:** 2025-07-22

**Authors:** Oluwafemi Ezekiel Kale, Sheng Hu, Claudia Huber, Felix Schierholz, Anna Ciotkowska, Alexander Tamalunas, Christian G. Stief, Wolfgang Eisenreich, Martin Hennenberg

**Affiliations:** ^1^ Department of Pharmacology and Therapeutics, Faculty of Basic Medical Sciences, Ago-Iwoye, Sagamu Campus, Olabisi Onabanjo University, Ago Iwoye, Nigeria; ^2^ Department of Urology, LMU University Hospital, LMU Munich, Munich, Germany; ^3^ Bavarian NMR Center - Structural Membrane Biochemistry, Department of Chemistry, Technical University of Munich, Munchen, Germany

**Keywords:** *Acridocarpus smeathmannii* root extract, lower urinary tract symptoms, porcine arteries, smooth muscle contraction, phytomedicine

## Abstract

**Introduction:**

The limited tolerability and efficacy of synthetic drugs have hindered the effective management of lower urinary tract symptoms (LUTSs). In African traditional medicine, species of the genus *Acridocarpus* (Malpighiaceae) are commonly used to treat reproductive disorders. In this study, we investigated the bioactivity-guided effects of *Acridocarpus smeathmannii* on smooth muscle contractility using human tissues obtained from radical prostatectomy and cystectomy procedures, as well as porcine coronary and interlobar arteries. Additionally, the impact of *A. smeathmannii* on the proliferation of cultured prostate stromal cells was evaluated.

**Methods:**

Cumulative concentration–response curves were generated for both adrenergic and cholinergic agonists, and electrical field stimulation (EFS) in organ bath experiments. In addition, assays were conducted to evaluate cell proliferation and viability, providing complementary insights into functional and cellular-level effects. The bioactive compounds in the extract were characterized using gas chromatography–mass spectrometry (GC-MS) and nuclear magnetic resonance spectroscopy and were subsequently evaluated *in silico* for their interaction with the α_1_-adrenergic receptor.

**Results:**

Prostate tissue contractions induced by α_1_-adrenergic agonists (0.1–100 µM) were reduced by 50% or more with *A. smeathmannii* at concentrations of 0.25 and 0.50 mg/mL. Bladder tissue contractions induced by the cholinergic agonists (0.1–1000 µM) were reduced by over two-thirds. Neurogenic contractions induced by EFS (2–32 Hz) were inhibited by up to 90% in both prostate and bladder tissues. Similarly, *A. smeathmannii* moderately inhibited contractile responses in porcine arteries. Moreover, *A. smeathmannii* inhibited the proliferation and viability of cultured prostate stromal cells in a concentration-dependent manner. *In silico* studies revealed that stigmasterol and pinostrobin chalcone showed the highest binding affinity to the α_1_-adrenergic receptor.

**Conclusion:**

In this study, we report for the first time that *A. smeathmannii* extract inhibits α_1_-adrenergic and cholinergic contractions in the prostate, bladder, and porcine arteries, with effects comparable to those of α_1_-blockers and anticholinergics. Additionally, *in silico* studies revealed that phytosterols, flavonoids, and benzoate esters in the extract exhibit supportive binding affinity to the α_1_-adrenergic receptor. Hence, *A. smeathmannii* may hold promise as a potential therapeutic agent for mixed-type LUTS.

## 1 Introduction

The global incidence of lower urinary tract symptoms (LUTSs), and benign prostatic hyperplasia (BPH) and overactive bladder (OAB) syndromes has been increasing in recent times (Chughtai et al., 2016; Wang X. et al., 2020; Takeuchi et al., 2023). Voiding symptoms attributed to BPH are characterized by impaired bladder emptying and weak urinary flow, whereas storage symptoms in OAB include urinary urgency, daytime frequency, nocturia, and incontinence (Przydacz et al., 2020). Both storage and voiding disorders may affect more than 60% of men and women worldwide (Huang et al., 2023). A considerable proportion of patients with LUTS suggestive of BPH also suffer from OAB-related symptoms, collectively referred to as mixed LUTS (Gravas et al., 2021).

Storage symptoms in OAB are caused by involuntary, uncontrolled smooth muscle contractions in the urinary bladder wall (detrusor muscle), where cholinergic voiding contractions during bladder emptying are induced by neurogenic activation of muscarinic acetylcholine receptors (Hennenberg and Michel, 2023). Voiding symptoms result from urethral obstruction, caused by increased smooth muscle tone and prostatic enlargement in the hyperplastic prostate, most commonly as a direct consequence of BPH (Chughtai et al., 2016; Takeuchi et al., 2023). Consequently, smooth muscle tone and cell proliferation are important targets for medical therapy in BPH and OAB (Adan et al., 2016; Przydacz et al., 2020). Available drugs include α_1_-adrenoceptor antagonists (α_1_-blockers), used for LUTS suggestive of BPH, and muscarinic receptor antagonists (anticholinergics), used for OAB, as contractions are induced by the activation of α_1_-adrenoceptors in the prostate and muscarinic receptors in the detrusor (Hennenberg and Michel, 2023). 5α-Reductase inhibitors are used to inhibit prostate growth, delaying progression and resulting in complications in BPH. Phosphodiesterase-5 inhibitors, such as tadalafil, and β_3_-adrenoceptor agonists are alternative therapeutic options. However, α_1_-blockers do not improve international prostate symptom scores (IPSSs) and urinary flow rate (Q_max_) by more than 50% (Watanabe et al., 2020). Disappointing efficacy, combined with inappropriate adverse events, contributes to high discontinuation rates, which can reach up to 90% with combination therapies in BPH within 12 months after the first prescription (Koudonas et al., 2023). Discontinuation leads to disease progression due to ongoing hyperplastic growth, complications (including painful infections or impaired renal function, both requiring emergency care), hospitalization, and surgery for BPH (Koudonas et al., 2023).

Erectile dysfunction (ED) and hypertension are common comorbidities of BPH and LUTS, both caused by exaggerated vasocontraction. Thus, inhibition of vasocontraction by antihypertensives and induction of vasorelaxation by phosphodiesterase-5 inhibitors in the corpus cavernosum are strategies for their medical treatment. The shared implications of phosphodiesterase-5 inhibitors for drug treatment in BPH and ED point to the potential for simultaneous action of a single drug to treat age-dependent comorbidities, which may reduce adverse drug effects (Watanabe et al., 2020). The progression and severity of LUTS may result from the maturation of cardiovascular disease (Vignozzi et al., 2016). Several reports have documented mechanistic links between LUTS and cardiovascular health, suggesting that the latter may adversely affect one or more organs, thereby contributing to the development of LUTS (He et al., 2016; Bauer et al., 2022).

Despite the clear need for medical attention and disease management, some patients turn to traditional medicine due to treatment costs, negative attitudes toward synthetic drugs, or undisclosed reasons (Kale and Awodele, 2016). Drug development from medicinal plants is at the forefront of identifying and developing new candidate compounds, including their subsequent chemical modification and application in bioactivity studies. This serves as the basis for introducing plant preparations as alternatives to synthetic drugs. Recently, some medicinal products were recommended for treating voiding symptoms by the guidelines for managing non-neurogenic male LUTS (Adler et al., 2012; Brookman-May et al., 2019). The recommendation followed meta-analyses supporting preclinical findings to improve prostate functions, with effect sizes resembling those of α_1_-blockers *in vitro* (Tamalunas et al., 2022). Plants and plant products have been used by large populations worldwide to treat various ailments, so the global focus on complementary and alternative plant-based medicine is increasing. Thus, ongoing research on phytomedicine is in high demand to explore and provide evidence of their efficacy, identify bioactive compounds, and discover novel candidates for drug development while also meeting the high patient demand for phytotherapy.

*Acridocarpus smeathmannii* (DC.) Guill. and Perr. (Malpighiaceae) is a tropical West and sub-tropical African plant, and its roots have been used traditionally to rejuvenate reproductive health (Catarino et al., 2016). Despite its long-standing use in African traditional medicine (ATM) for treating reproductive disorders, scientific evidence supporting its application remains limited. Studies reflecting the interest in this plant family have investigated its antioxidant (Rehman et al., 2021; Konaré et al., 2024), hematological (Kale et al., 2019b), immunomodulatory (Jamshidi-Adegani et al., 2020), sexual and reproductive (Kale et al., 2019a), hepatoprotective (Lotfy et al., 2020), and cytotoxic activities (Cao et al., 2004; Balhamar et al., 2019). To the best of our knowledge, reports documenting its potential for LUTS are limited; however, impaired reproductive function and voiding symptoms are common comorbidities in men, and both depend on smooth muscle function.

The aim of the present study was to explore the potential of *A. smeathmannii* extracts on LUTS-relevant functions, including prostate and bladder smooth muscle contractions, as well as the growth of prostate stromal cells. This was accompanied by the identification of bioactive candidate compounds and the assessment of inhibitory effects on vasocontraction.

Therefore, in this study, we provide information on the potential use of *A. smeathmannii* root extract for multiple indications, based on its bioactivity in controlling smooth muscle tone across different lower urinary tract organs and blood vessels. In addition, bioactive compounds in the extract were subsequently evaluated *in silico* for their interaction with the α_1_-adrenergic receptor to ascertain their potential involvement in the possible pharmacological effects.

## 2 Materials and methods

### 2.1 Drugs and chemicals

Phenylephrine (PHE), noradrenaline (NA), carbachol (CcH), methacholine (McH), n-hexane, CDCl_3_, KCl, and Cell Counting Kit-8 (CCK-8) were purchased from Sigma-Aldrich (St. Louis, MO, United States). 5-Ethynyl-2ʹ-deoxyuridine (EdU) solution, the EdU-Click 555 Cell Proliferation Assay Kit (Baseclick, Munich, Germany), 4ʹ,6-diamidino-2-phenylindole (DAPI), 96-well plates, and 16-well chambered coverslips were obtained from Thermo Fisher Scientific (Munich, Germany).

### 2.2 Plant collection and authentication

Leaves and roots of *Acridocarpus smeathmannii* were collected in 2023 from a farmland secondary settlement forest in Akinmorin village, Oyo State, Nigeria (7°51′9.25″N, 3°55′52.5″E; 298 m above sea level). The collection was processed out by Dr. Odewo A. Samuel, a botanist at the Forest Research Institute of Nigeria (FRIN), Ibadan. The plant voucher was deposited in the FRIN herbarium (Voucher No. FHI: 113685) and at the University of Lagos Herbarium (LUH 6638) by Dr. O. O. Oyebanji. Research involving the plant was approved by the Health Research and Ethics Committee, College of Medicine, the University of Lagos (CMUL/HREC/09/18/424). In addition, a phytosanitary certificate (No. 0124876) was issued by the Nigeria Agricultural Quarantine Service. The plant material was dried at approximately 23°C and pulverized using a Christy and Norris Lab Mill (No. 50158, England) at the Department of Pharmacognosy, Olabisi Onabanjo University, Nigeria.

### 2.3 Soxhlet extraction

A Soxhlet extractor thimble was loaded with 25 g of *A. smeathmannii* root powder and extracted with n-hexane (EMPLURA^®^, Merck KGaA, Germany) (Kale et al., 2025).

### 2.4 Gas chromatography–mass spectrometry analysis

Gas chromatography–mass spectrometry (GC-MS) analysis was performed using a Shimadzu QP2010 Plus instrument with a fused silica capillary column (Equity TM-5; 30 m × 0.25 mm, 0.25 µm film thickness; Supelco Merck KGaA, Darmstadt, Germany) (Kale et al., 2025).

### 2.5 Nuclear magnetic resonance analysis

Nuclear magnetic resonance (NMR) spectroscopy was performed using 5 mg of *A. smeathmannii* extract dissolved in 600 μL of CDCl_3_ (Kale et al., 2025).

### 2.6 Contraction measurements with human prostate and detrusor tissues

This study was in line with the Declaration of Helsinki and was approved by the Ethics Committee of Ludwig-Maximilians University (LMU), Munich, Germany. Informed consent was obtained from all patients. Prostate (periurethral zone) and bladder (lateral wall) tissues were collected from patients undergoing radical prostatectomy or cystectomy, respectively. Only tissues free from macroscopic tumor infiltration were used. The urothelial layer was removed from bladder samples to isolate the detrusor muscle. Tissues were transported in Custodiol^®^ solution (Köhler, Bensheim, Germany), and experiments were initiated within 3 h of collection. Tissue strips (6 × 3 × 3 mm) were mounted in 10 mL tissue baths (Danish Myotechnology, Denmark) containing Krebs–Henseleit solution (37°C, pH 7.4) and aerated with 95% O_2_ and 5% CO_2_. After equilibration at 4.9 mN for 45 min, maximum contraction was induced with 80 mM KCl. Tissues were rinsed and treated with either *A. smeathmannii* extract (0.05–0.5 mg/mL final concentration) or ethanol control (1%). Cumulative concentration–response curves for NA and PHE in prostate tissues, as well as for CcH and McH in bladder tissues, were generated 30 min after treatment. Each sample was divided into control and *A. smeathmannii*-treated groups for intra-patient comparisons. Contractile responses were normalized to the KCl-induced maximum (100%). Dose–response curves were fitted using GraphPad Prism (GraphPad Software Inc., San Diego, CA, United States) to calculate maximum agonist-induced contraction (EC_50_), maximum possible contraction (E_max_), and maximum EFS-induced contraction (Ef_50_) values. EC_50_ values were also expressed as pEC_50_ (-log molar concentration) (Kale et al., 2025; Keller et al., 2025).

### 2.7 Porcine interlobar and coronary arteries

Porcine hearts and kidneys were obtained from a local abattoir (Metzgerei Brehm, Planegg, Germany), in compliance with LMU ethics approval (LMU/MH060922). Organs were kept at 4°C (Custodiol^®^ solution) and processed within 2 h. Segments of coronary and interlobar arteries were dissected, cleared of connective and adipose tissue, and cut into rings (3–4 mm). Arterial rings were mounted in tissue baths under a resting tension of 9.8 mN (interlobar) or 19.8 mN (coronary) (Huang et al., 2022), and adjusted during a 45-min equilibration period. Functional studies with *A. smeathmannii* and controls were conducted similarly to prostate and bladder tissue protocols.

### 2.8 Determination of *Acridocarpus smeathmannii* effects on the prostate or detrusor smooth muscle contractile activity

The anticontractile effects of increasing concentrations of *A. smeathmannii* extract (0.05, 0.1, 0.25, and 0.5 mg/mL) on prostate and bladder tissues were assessed by comparing to contractions induced with ethanol (1%) as a vehicle control. Contractions were elicited using NA (nonselective) and PHE (α_1_-selective) agonists (0.1–100 µM) in prostate tissues and using CcH and McH (0.1–1,000 µM) in bladder tissues. Anticontractile effects (*A. smeathmannii* extract or vehicle) were expressed as a positive percentage relative to the contraction induced by the agonist (Kale et al., 2025; Keller et al., 2025).

### 2.9 Determination of *Acridocarpus smeathmannii* effects on the porcine coronary and interlobar artery contractility

The anticontractile effects of increasing concentrations of *A. smeathmannii* on porcine coronary and interlobar arteries were assessed using a similar protocol. Contractions were induced using CcH (0.1–1,000 µM) in coronary arteries and using NA (0.1–100 µM) in interlobar arteries. A reduction in agonist-induced tone by *A. smeathmannii* compared to the corresponding vehicle control was considered an anticontractile effect and expressed as a positive percentage relative to the control response (Huang et al., 2022).

### 2.10 Electrical field stimulation

Electrical field stimulation (EFS) was used to generate frequency–response curves for contractions mediated by neurogenic activation, 30 min after the addition of *A. smeathmannii* extract or vehicle (ethanol). EFS evokes action potentials that cause the release of endogenous neurotransmitters, including noradrenaline and acetylcholine. Tissue strips were mounted between two parallel platinum electrodes connected to a CS4 stimulator (Danish Myotechnology Aarhus, Denmark). Square-wave pulses (positive monophasic) of 1 m duration and 20 V amplitude were delivered at frequencies of 2, 4, 8, 16, and 32 Hz, with 60-s intervals between stimulations. Only one frequency–response curve was recorded for each sample. EFS-induced contraction amplitudes were expressed as a percentage of the maximal response to 80 mM KCl. E_max_ and the frequency producing 50% of maximal EFS-induced contraction (Ef_50_) were calculated using curve fitting in GraphPad Prism.

### 2.11 Cell proliferation assay

WPMY-1 cells were cultured in a medium containing 10% fetal calf serum (FCS) and 1% penicillin/streptomycin at 37°C in a 5% CO_2_ atmosphere and seeded at a density of 50,000 cells/well on 16-well chambered coverslips. Cells were treated with 10 µL of *A. smeathmannii* extract at concentrations of 0.05, 0.10, and 0.25 mg/mL or with vehicle control and incubated for 12 h. Parallel experiments were performed with 24- and 48-h incubation periods. Following treatment, the medium was replaced with 10 mM EdU in an FCS-free medium containing the respective treatments, and cells were fixed with 3.7% formaldehyde. EdU incorporation into DNA was detected using a fluorescent 5-carboxy tetramethylrhodamine probe. Nuclear counterstaining was performed using DAPI. Fluorescence microscopy was used for analysis (excitation: 546 nm; emission: 479 nm) (Tamalunas et al., 2022).

### 2.12 Cell viability assay

The cell viability in response to *A. smeathmannii* extract was assessed in 96-well plates seeded with 20,000 cells/well and incubated for 24 h. Cells were treated for 12, 24, or 48 h, after which 10 μL of WST-8 reagent from the CCK-8 was added to each well. Absorbance at 450 nm was measured after 2 h of incubation at 37°C (Wang H. et al., 2020).

### 2.13 Binding affinity and *in vitro* pharmacokinetic, physicochemical, and medicinal chemistry properties

The cryo-EM structure of the alpha1A-adrenergic receptor (α1AAR) bound to tamsulosin (complexed with Nb6) is available from the Protein Data Bank (PDB ID: 7YMJ). Protein structures were visualized and analyzed using Swiss-PdbViewer 4.1.0 and Biovia Discovery Studio 2024, respectively. Chemical structures of GC-MS-identified compounds from the hexane root extract of *A. smeathmannii* were retrieved from ChemAxon and PubChem and converted to a PDB format using Open Babel. These PDB files, with Gasteiger charges, were converted to PDBQT format for protein–ligand docking using AutoDock Vina v4.2.6. Docking results were visualized using PyMOL 2.6.0, and interaction analyses were performed in Biovia Discovery Studio to generate 2D diagrams and 3D visualizations. Pharmacokinetic, physicochemical, and medicinal chemistry properties were evaluated using the SwissADME and pkCSM web servers.

### 2.14 Data and statistical analyses

Data from concentration–response and frequency–response curves are expressed as mean ± standard deviation (SD). *Post hoc* analyses of multiple comparisons at different agonist concentrations or stimulation frequencies were conducted using two-way ANOVA with multiple comparisons. Cell culture results were analyzed using repeated-measures one-way ANOVA and column statistics. All data are presented as scatterplots, showing individual values from each independent experiment (Michel et al., 2020). Each series of organ bath and cell culture experiments consisted of *n* = 5 independent replicates with paired samples. Statistical analyses were performed using GraphPad Prism 9.5.0 (GraphPad Software Inc., San Diego, CA, United States). E_max_, pEC_50_, and Ef_50_ values were compared using paired t-tests. Changes in responses are expressed as percentage differences relative to the control [mean difference (MD) with 95% confidence intervals], normalized to the KCl-induced maximal contraction.

## 3 Results

### 3.1 Effects of *Acridocarpus smeathmannii* on adrenergic contractions of human prostate tissues

**FIGURE 1:**
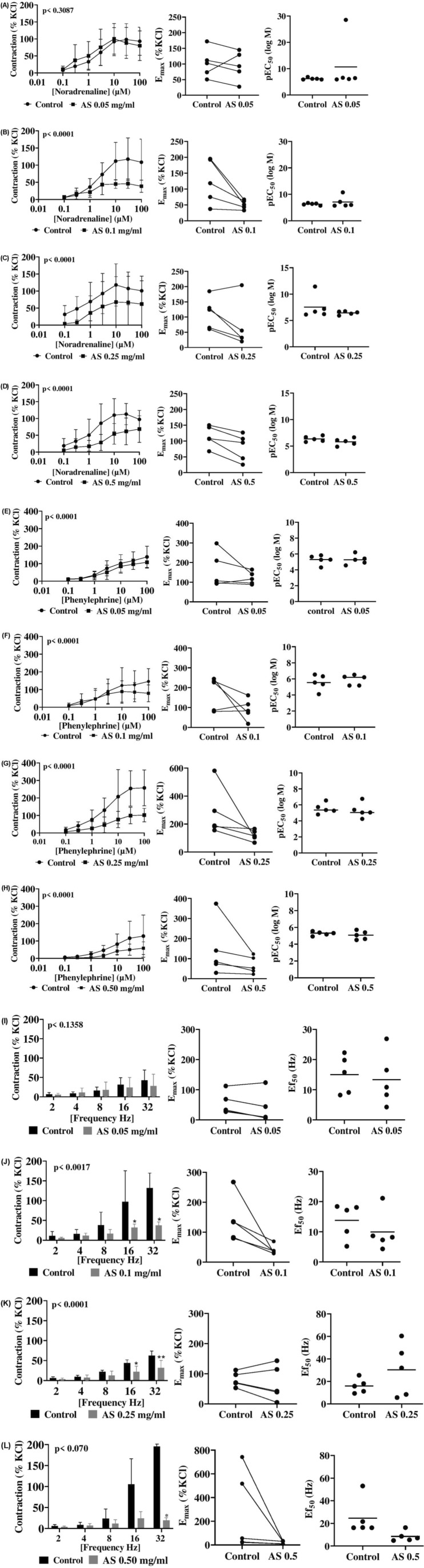
Effects of *Acridocarpus smeathmannii* extract on adrenergic human prostate smooth muscle contraction. PR, prostate. Contractions in an organ bath were induced by noradrenaline **(A–D)**, phenylephrine **(E–H)**, and EFS **(I–L)**. Results are expressed as mean ± SD (n = 5 patients per series, with tissue from each patient split between the *A. smeathmannii* extract and ethanol control groups). Tensions are expressed as a percentage of the high-molar KCl-induced contraction assessed prior to application of either the ethanol control or *A. smeathmannii*. E_max_ and pEC_50_ or Ef_50_ were calculated by curve fitting for each experiment.

Figure 1 shows contractions of human prostate tissue induced by the nonselective adrenergic agonist NA. Following incubation with *A. smeathmannii* (0.05–0.50 mg/mL) or ethanol (control), the NA response (0.1–100 µM) was concentration dependent but attenuated by the extract compared to controls (Figures 1A–D; Table 1). At 0.05 mg/mL *A. smeathmannii*, NA-induced contractions were largely unaffected at most concentrations, with only a small reduction [up to 12% at 100 μM NA; MD 12.98 (−25.44 to 55.41) % of KCl]. However, higher *A. smeathmannii* concentrations produced sustained inhibition. In particular, 0.10 mg/mL *A. smeathmannii* reduced contractions by 52%–54% at 10, 30, and 100 µM NA [e.g., MD 66.8 (16.1–117.2) %, p = 0.005] (Figure 1B). At 0.25 mg/mL, contraction at 10 µM NA decreased by 51% [MD 50.3 (2.8–97.7) %] (Figure 1C). At 0.50 mg/mL, NA-induced contractions were reduced by 47%–57% across 3–30 µM [e.g., 57.3 (16.5–98.1) %, p = 0.003 at 3 µM] (Figure 1D). Curve-fitting revealed that *A. smeathmannii* (0.10 and 0.50 mg/mL) lowered E_max_ values, whereas EC_50_ shifts were minor and not statistically significant (Figures 1A–H, J–L).

**TABLE 1:** Mean differences (MDs) for agonist-induced contractions after application of AS extract or control and 95% confidence intervals (CIs) (in parentheses, low to high) (% of KCl-induced contractions).

				Agonist concentration							
			0.1 μM	0.3 μM	1 μM	3 μM	10 μM	30 μM	100 μM		
Prostate	AS 0.05	Noradrenaline	−2 [−40 to 37]	−17 [−56 to 21]	−17 [−56 to 21]	−15 [−54 to 23]	−8 [−47 to 30]	10 [−28 to 51]	13 [−25 to 51]	NA	NA
	AS 0.10		1 [−50 to 50]	−5 [−55 to 46]	15 [−35 to 65]	29 [−21 to 80]	67 [16 to 117	72 [21 to 122]	70 [19 to 120]	NA	NA
	AS 0.25		27 [−20 to 75]	40 [−8 to 87]	33 [−14 to 80	38 [−10 to 85]	50 [8 to 98]	41 [−7 to 88]	39 [−9 to 86]	NA	NA
	AS 0.50		14 [−27 to 55]	18 [−23 to 58]	33 [−8 to 74]	57 [17 to 98]	56 [15 to 97]	51 [10 to 91]	29 [−12 to 70]	NA	NA
Prostate	AS 0.05	Phenylephrine	1 [−48 to 51]	1 [48 to 51]	5 [45 to 54]	23 [−26 to 73]	17 [−32 to 67]	20 [−29 to 69]	31 [−19 to 80]	NA	NA
	AS 0.10		3 [−71 to 77]	13 [−60 to 87]	−2 [−76 to 71]	15 [−59 to 89]	34 [−39 to 108]	41 [−32 to 115]	67 [−7 to 140]		
	AS 0.25		11 [−128 to 151]	26 [−114 to 1,654]	50 [−90 to 189]	84 [−56 to 223]	130 [−9 to 270]	155 [15 to 294]	155 [15 to 295]		
	AS 0.50		3 [−57 to 63]	8 [−53 to 68]	21 [−40 to 81]	32 [−29 to 92]	40 [−21 to 100]	66 [6 to 126]	70 [109 to 130]		

Agonist concentration											
			0.1 μM	0.3 μM	1 μM	3 μM	10 μM	30 μM	100 μM	300 μM	1,000 μM
Bladder	AS 0.05	Carbachol	20 [−11 to 50]	22 [−8 to 53]	21 [−10 to 51]	37 [7 to 67]	38 [8 to 69]	34 [3 to 64]	42 [11 to 72]	34 [3 to 64]	32 [1 to 62]
	AS 0.10		7 [−33 to 47]	18 [−22 to 58]	39 [−1 to 79]	43 [3 to 83]	42 [1 to 82]	35 [−5.3 to 75]	18 [−22 to 58]	25 [−16 to 65]	23 [−17 to 63]
	AS 0.25		8 [−55 to 70]	32 [−31 to 94]	68 [6 to 131]	83 [21 to 146]	80 [17 to 142]	61 [−2 to 123]	82 [19 to 144]	62 [−0 to 125]	53 [−9. to 116]
	AS 0.50		7 [−80 to 94]	34 [−53 to 121]	78 [−9 to 166]	113 [26 to 200]	121 [34 to 208]	98 [11 to 185]	77 [−10 to 164]	38 [−60 to 135]	43 [−55 to 140]
Bladder	AS 0.05	Methacholine	7 [−33 to 46]	10 [−29 to 49]	25 [−14 to 64.62]	42 [2 to 81]	44 [5 to 84]	48 [9 to 88]	59 [20 to 99]	58 [18 to 97]	44 [4 to 83]
	AS 0.10		7 [−40 to 54]	38 [−8 to 85]	39 [−8 to 86]	64 [17 to 111]	62 [15 to 109]	62 [15 to 109]	46 [−1 to 92	46 [−1 to 93]	40 [−7 to 86]
	AS 0.25		9 [−38.9 to 58]	22 [−26 to 70]	38 [−19 to 87]	38 [−10 to 86]	29 [−19 to 77]	29 [−19 to 78]	31 [−18 to 79]	25 [−23 to 74]	20 [−28 to 68]
	AS 0.50		17 [−21 to 55]	28 [−10 to 66]	59 [21 to 97]	29 [−9 to 66]	28 [−10 to 66]	38 [−1 to 76]	28 [−10 to 66]	24 [−18 to 67]	22.1 [−20 to 65]

Calculations were performed for those agonists and tissues, where a possible inhibition of contraction by AS extract was observed in concentration–response curves. AS (0.05 mg/mL), AS (0.1 mg/mL), AS (0.25 mg/mL), and AS (0.5 mg/mL) are concentrations of AS extracts. For each single experiment, contractions with inhibitors were calculated as percent of the corresponding control in the same experiment and subtracted from the control [100 − (contraction with inhibitor)/(contraction control)×100], i.e., between inhibitor and control ethanol group, for corresponding, paired samples from the same prostate, bladder, coronary artery, or interlobar artery in each single experiment and are expressed as MD with 95% CI. Results are expressed as mean ± SD (n = 5 patients per series, with tissue from each patient split to both the AS extract and ethanol control groups).

PHE (0.1–100 µM), an α_1_-selective agonist, also induced concentration-dependent prostate contractions, which *A. smeathmannii* reduced in a dose-dependent manner (Figures 1E–H; Table 1). At 0.05 mg/mL *A. smeathmannii*, contraction at 100 µM PHE decreased by 17% [MD 30.7 (−18.6 to 80.0) %, p = 0.45] (Figure 1E). At 0.10 mg/mL *A. smeathmannii*, the reduction reached 36.8% [MD 67.0 (−6.7 to 140.7])%, p = 0.09] at the same PHE level (Figure 1F). At 0.25 mg/mL, reductions reached 52.9%–54% at 30 and 100 µM PHE (p = 0.02) (Figure 1G); similar effects occurred at 0.50 mg/mL (44.8%–51.2%; p = 0.02) (Figure 1H). *Acridocarpus smeathmannii* reduced E_max_ compared to ethanol controls.

### 3.2 Effects of *Acridocarpus smeathmannii* on electric field stimulation-induced contraction of human prostate tissues

Electric field stimulation (2–32 Hz) to produce neurogenic contraction showed that *A. smeathmannii* inhibited prostate responses (Figures 1I–L; Table 3). At 0.05 mg/mL *A. smeathmannii*, inhibitory effects reached 45% at 32 Hz [MD 14.5 (−1.7 to 31.0) %]. At 0.10 mg/mL, inhibition was 54.1% at 16 Hz [MD 65.0 (−1.9 to 131.9), p = 0.05] and 63.9% at 32 Hz [MD 94.7 (27.8–161.5)]. At 0.25 mg/mL, reductions were 50% at both 16 Hz [MD 22.0 (9.7–34.3), p = 0.003] and 32 Hz [MD 30.6 (18.3–42.9), p < 0.0001]. At 0.50 mg/mL, inhibition reached 68% at 32 Hz [MD 176.0 (−5.0 to 357.0), p = 0.05]. *Acridocarpus smeathmannii* decreased E_max_ values for EFS-induced contractions; Ef_50_ was unaffected except at 0.50 mg/mL.

### 3.3 Effects of *Acridocarpus smeathmannii* on cholinergic contractions of human bladder tissues

**FIGURE 2:**
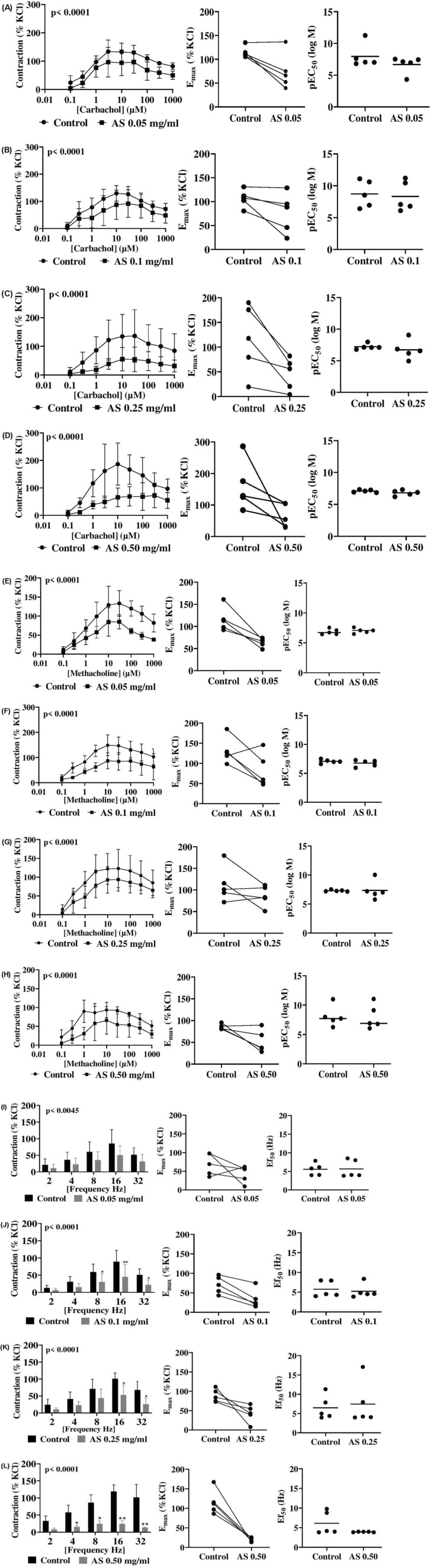
Effects of *Acridocarpus smeathmannii* extract on cholinergic human detrusor (bladder) smooth muscle contraction. DSM, detrusor smooth muscle. Contractions in an organ bath were induced by carbachol **(A–D)**, methacholine **(E–H)**, and EFS **(I–L)**. Results are expressed as mean ± SD (n = 5 patients per series, with tissue from each patient split between the *A. smeathmannii* extract and ethanol control groups). Tensions are expressed as a percentage of the high-molar KCl-induced contraction assessed prior to application of either the ethanol control or *A. smeathmannii*. E_max_ and pEC_50_ or Ef_50_ were calculated by curve fitting for each experiment.

Carbachol-induced (0.1–1,000 µM) contractions of detrusor smooth muscle were attenuated by *A. smeathmannii* (0.05–0.50 mg/mL) (Figures 2A–D; Table 1). At 0.05 mg/mL, inhibition reached 39.1% at 100 µM [MD 41.5 (11.1–72.0) %, p = 0.003]. At 0.10 mg/mL, inhibition was 47.2% at 3 µM [MD 43.4 (3.3–83.4), p = 0.03] and 35% at 10 µM [MD 42.0 (2.0–82.0), p = 0.03]. At 0.25 mg/mL, inhibition ranged between 57% and 72% across 1–30 µM (p < 0.01–0.03). At 0.50 mg/mL, inhibition reached 59%–62% at 3–30 µM (all p < 0.01–0.02). E_max_ values were reduced at 0.10 and 0.50 mg/mL, whereas EC_50_ remained unchanged.

Methacholine-induced (0.1–1,000 µM) bladder contractions were similarly inhibited by *A. smeathmannii* (Figures 2E–H). At 0.05 mg/mL, inhibition ranged from 33% to 59% across 3–1,000 µM (p < 0.01–0.032). At 0.10 mg/mL, inhibition was 48% (3 µM), 40% (10 µM), and 42% (30 µM) (all p < 0.001–0.004). Both 0.25 and 0.50 mg/mL produced 44% and 65% inhibition at 1 µM. E_max_ values were reduced by *A. smeathmannii* at 0.05–0.25 mg/mL.

### 3.4 Effects of *Acridocarpus smeathmannii* on electric field stimulation-induced contractions of human bladder tissues

*Acridocarpus smeathmannii* inhibited EFS-induced bladder contractions (2–32 Hz) (Figures 2I–L; Table 3). At 0.05 mg/mL, inhibition reached 30.7% at 16 Hz and 15.8% at 32 Hz (p > 0.05). At 0.10 mg/mL, contraction was reduced by 52%–58% across 8–32 Hz (p < 0.001). At 0.25 mg/mL, inhibition ranged from 48% to 52% at 16–32 Hz (p < 0.001–0.003). At 0.50 mg/mL, inhibition reached 69%–86% across 16–32 Hz (p < 0.001–0.009). *Acridocarpus smeathmannii* lowered E_max_ without significantly altering Ef_50_.

### 3.5 Effects of *Acridocarpus smeathmannii* on vascular tissues (porcine coronary and interlobar arteries)

**FIGURE 3:**
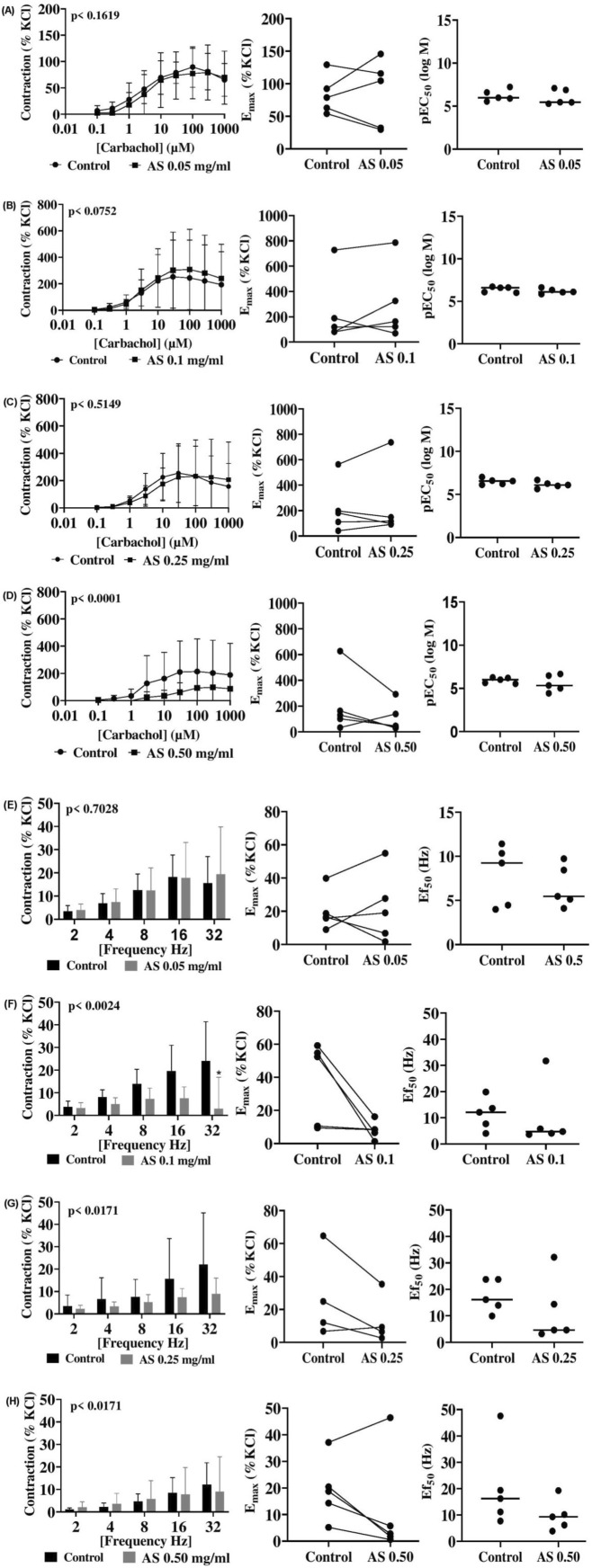
Effects of *Acridocarpus smeathmannii* extract on cholinergic porcine coronary artery smooth muscle contraction. Contractions in an organ bath were induced by carbachol **(A–D)** and EFS **(E–H)**. Results are expressed as mean ± SD (n = 5 animals). Tensions are expressed as a percentage of the high-molar KCl-induced contraction assessed prior to application of either the ethanol control or *A. smeathmannii*. E_max_ and pEC_50_ or Ef_50_ were calculated by curve fitting for each experiment.

In porcine coronary artery, *A. smeathmannii* (less than 0.50 mg/mL) produced only mild, insignificant inhibition of carbachol-induced contraction (less than 14%) (Figures 3A–D; Table 2). EFS at 32 Hz was inhibited by 76% at 0.10 mg/mL (p = 0.01) and 46% at 0.25 mg/mL (p = 0.06); a concentration of 0.50 mg/mL had no significant effect (Figures 3E–H; Table 3).

**TABLE 2:** Mean differences (MDs) for agonist-induced contractions on porcine coronary and interlobar arteries after application of AS extract or control and 95% confidence intervals (CIs) (in parentheses, low to high) (% of KCl-induced contractions).

			Agonist concentration								
			0.1 μM	0.3 μM	1 μM	3 μM	10 μM	30 μM	100 μM	300 μM	1,000 μM
Coronary artery	AS 0.05	Carbachol	4 [−32 to 40]	9 [−28 to 45]	10 [−26 to 46]	10 [−26 to 46]	5 [−31 to 41]	5 [−31 to 41]	12 [−24 to 49]	2 [−35 to 38]	−4 [−40 to 32]
	AS 0.10		2 [−123 to 126]	12 [−113 to 136]	14 [−111 to 138]	−20 [−145 to 105]	−21 [−145 to 104]	−50 [−174 to 75]	−64 [−188 to 61]	−59 [−184 to 65]	−46 [−171 to 78]
	AS 0.25		2 [−93 to 97]	1 [−94 to 96]	16 [−79 to 111]	51 [−44 to 147]	50 [−45 to 145]	30 [−66 to 125]	2 [−93 to 97]	−39 [−134 to 56]	−50 [−145 to 45]
	AS 0.50		1 [−169 to 169]	16 [−153 to 185	36 [−133 to 204]	102 [−66 to 271]	127 [−42 to 296]	149 [−20 to 317]	122 [−47 to 290]	106 [−63 to 274]	102 [−67 to 270]
			0.1 μM	0.3 μM	1 μM	3 μM	10 μM	30 μM	100 μM		
Interlobar artery	AS 0.05	Noradrenaline	7 [−61 to 75]	26 [−43 to 94]	20 [−48 to 88]	−6 [−74 to 62]	−18 [−86 to 51]	−12 [−80 to 56]	−24 [−93 to 44]	NA	NA
	AS 0.10		49 [−186 to 283]	75 [−160 to 309]	106 [−128 to 341]	122 [−112 to 357]	122 [−113 to 356]	111 [−123 to 346]	120 [−115 to 354]	NA	NA
	AS 0.25		53 [−240 to 346]	93 [−200 to 386]	82 [−211 to 375]	26 [−267 to 319]	−24 [−317 to 269]	−44 [−337 to 250]	−49 [−342 to 244]	NA	NA
	AS 0.50		−2 [−86 to 82]	28 [−56 to 113]	28 [−56 to 113]	49 [−35 to 133]	75 [−9 to 160]	83 [−1 to 168]	92 [8 to 177]	NA	NA

Calculations were performed for those agonists and tissues, where a possible inhibition of contraction by AS extract was observed in concentration–response curves. AS (0.05 mg/mL), AS (0.1 mg/mL), AS (0.25 mg/mL), and AS (0.5 mg/mL) are concentrations of AS extracts. For each single experiment, contractions with inhibitors were calculated as percent of the corresponding control in the same experiment and subtracted from the control: [100 − (contraction with inhibitor)/(contraction control)×100], i.e., between inhibitor and control ethanol groups, for corresponding, paired samples from the same prostate, bladder, coronary artery, or interlobar artery in each single experiment and are expressed as MD with 95% CI. Results are expressed as mean ± SD (n = 5 patients per series, with tissue from each patient split to both the AS extract and ethanol control group).

**TABLE 3:** Mean differences (MDs) for EFS-induced contractions on prostate, bladder, and porcine arteries after application of AS extract or control and 95% confidence intervals (CIs) (in parentheses, low to high) (% of KCl-induced contractions).

			Neurogenic stimulation				
Prostate	Freq		2 Hz	4 Hz	8 Hz	16 Hz	32 Hz
	AS 0.05	EFS	10 [−31 to 51]	14 [−27 to 55]	24 [−16 to 65]	35 [−6 to 76]	20 [−21 to 61]
	AS 0.10		6 [−11 to 23]	15 [−2 to 32]	29 [12 to 45]	44 [28 to 61]	29 [12 to 46]
	AS 0.25		13 [−21 to 47]	17 [−16 to 52]	27 [−7 to 61]	15 [15 to 82]	42 [9 to 76]
	AS 0.50		25 [−8 to 58]	42 [9 to 75]	62 [29 to 95]	94 [61 to 127]	89 [55 to 121]
Bladder			2 Hz	4 Hz	8 Hz	16 Hz	32 Hz
	AS 0.05	EFS	10 [−31 to 51]	14 [−27 to 55]	24 [−16 to 65]	35 [−6 to 76]	20 [−21 to 61]
	AS 0.10		6 [−11 to 23]	15 [−2 to 32]	29 [12 to 45]	44 [28 to 61]	29 [12 to 46]
	AS 0.25		13 [−21 to 47]	18 [−16 to 52]	27 [−7 to 61]	48 [15 to 82]	42 [9 to 76]
	AS 0.50		25 [−8 to 58]	42 [9 to 75]	62 [29 to 95]	94 [61 to 127]	89 [55 to 122]
			2 Hz	4 Hz	8 Hz	16 Hz	32 Hz
Coronary artery	AS 0.5	EFS	−1 [−14 to 14]	−1 [−15 to 13]	0.2 [−14 to 14]	0.4 [−14 to 14]	−4 [−18 to 10]
	AS 0.10		1 [−16 to 17]	4 [−13 to 20]	7 [−9 to 23]	13 [−4 to 29]	21 [4 to 37]
	AS 0.25		1 [−13 to 15]	3 [−11 to 17]	2 [−11.4 to 16]	8 [−6 to 22]	13 [−1 to 27]
	AS 0.50		1 [−8 to 6]	−1 [−8 to 6]	−1 [−8 to 6]	1 [−6 to 8]	3 [−4 to 10]
			2 Hz	4 Hz	8 Hz	16 Hz	32 Hz
Interlobar artery	AS 0.05	EFS	5 [−95 to 104]	5 [−94 to 105]	24 [−76 to 123]	86 [−13 to 185]	138 [39 to 238]
	AS 0.10		1 [−31 to 32]	1 [−31 to 32]	6 [−25 to 38]	8 [−23 to 40]	7 [−24 to 38]
	AS 0.25		2 [−22 to 26]	2 [−26 to 22]	3 [−21 to 27]	14 [−10 to 38]	26 [2 to 50]
	AS 0.50		−2 [−29 to 24]	1 [−27 to 26]	4 [−22 to 31]	53 [27 to 80]	85 [59 to 111]

Calculations were performed for those agonists and tissues, where a possible inhibition of contraction by AS extract was observed in concentration–response curves. AS (0.05 mg/mL), AS (0.1 mg/mL), AS (0.25 mg/mL), and AS (0.50 mg/mL) are concentrations of AS extracts. NA, not applicable. For each single experiment, contractions with inhibitors were calculated as percent of the corresponding control in the same experiment and subtracted from the control: [100 − (contraction with inhibitor)/(contraction control)×100], i.e., between inhibitor and control ethanol group, for corresponding, paired samples from the same prostate, bladder, coronary artery, or interlobar artery in each single experiment and are expressed as MD with 95% CI. Results are expressed as mean ± SD (n = 5 patients per series, with tissue from each patient split to both the AS extract and ethanol control groups).

**FIGURE 4:**
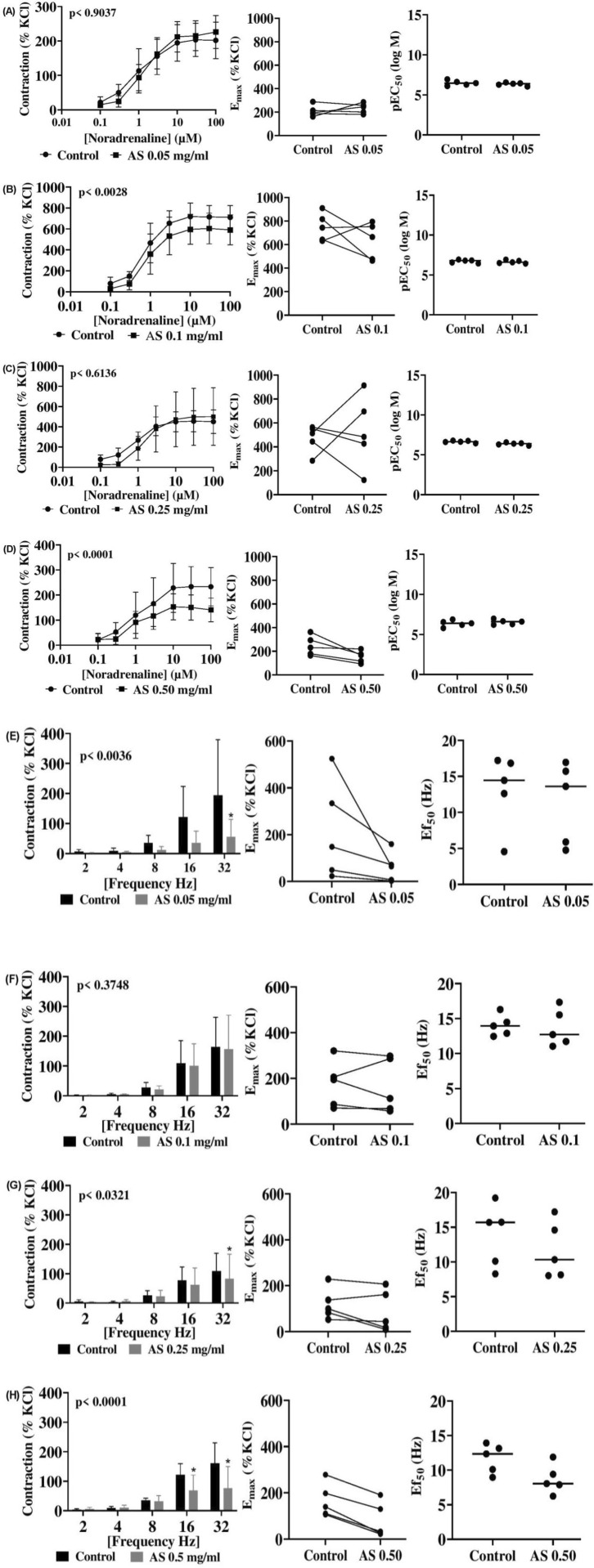
Effects of *Acridocarpus smeathmannii* extract on adrenergic porcine interlobar artery smooth muscle contraction. Contractions in an organ bath were induced by noradrenaline **(A–D)** and EFS **(E–H)**. Results are expressed as mean ± SD (n = 5 animals). Tensions are expressed as a percentage of the high-molar KCl-induced contraction assessed prior to application of either the ethanol control or *A. smeathmannii*. E_max_ and pEC_50_ or Ef_50_ were calculated by curve fitting for each experiment.

In porcine interlobar artery, NA-induced contractions were inconsistently inhibited at less than 0.25 mg/mL, but at 0.50 mg/mL, 100 µM NA-induced contraction decreased by 38.1% (p = 0.03) (Figures 4A–D). For EFS, 0.05 mg/mL *A. smeathmannii* reduced contraction by 78.7% at 32 Hz (p = 0.004); a concentration of 0.25 mg/mL showed a 35% decrease at 32 Hz (p = 0.03); at 0.50 mg/mL, inhibition at 16 and 32 Hz reached 47%–60% (p < 0.001). E_max_ and Ef_50_ remained unchanged.

### 3.6 Effects of *Acridocarpus smeathmannii* on the viability of WPMY-1 cells

**FIGURE 5:**
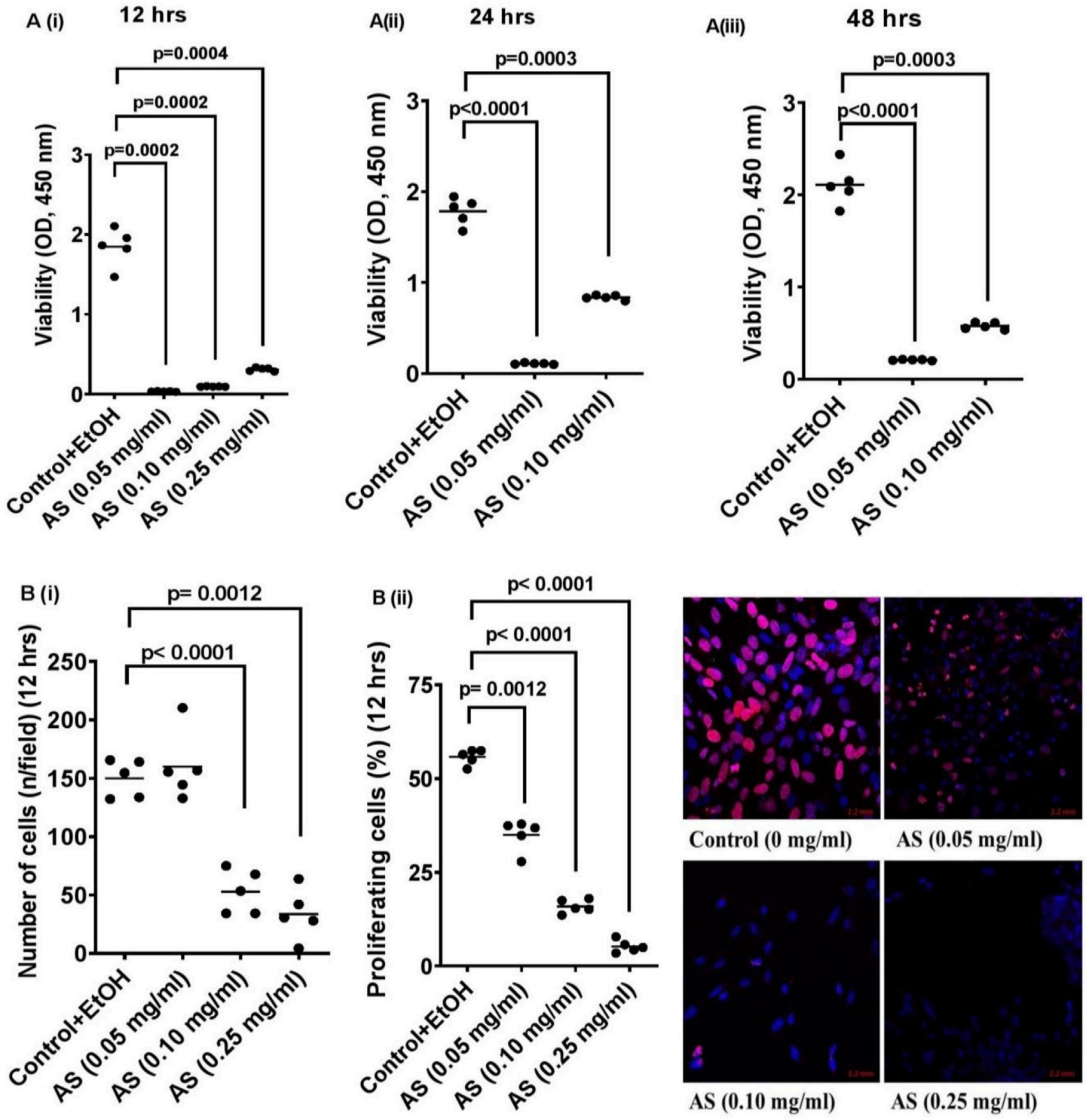
Effects of *Acridocarpus smeathmannii* on cell viability and proliferation of prostate stromal cells (WPMY-1). Viability was assessed using Cell Counting Kit-8 (CCK8), whereas proliferation was assessed using EdU assays. The effects of *Acridocarpus smeathmannii* (0.05, 0.1, and 0.25 mg/mL) are concentrations of *A. smeathmannii* on the **(A)** CCK8 assays showing viability of WPMY-1 cells in the time periods as indicated (i–iii) and **(B)** EdU assays showing the proliferation of WPMY-1 cells in the concentrations indicated **B** (i,ii). Ethanol-treated cells under same condition were used as control. Results are presented as mean (n = 5 independent experiments). p < 0.05 was considered significant versus control. OD, optical density.

CCK-8 assays demonstrated a profound, dose-dependent reduction in WPMY-1 cell viability. At 12 h, viability decreased by 98.2% (0.05 mg/mL), 94.5% (0.10 mg/mL), and 83.8% (0.25 mg/mL) compared to controls (Figure 5Ai). After 24 h, colony formation decreased by 94.2% and 50.6% at 0.05 and 0.10 mg/mL, respectively (Figure 5Aii), and by 90.1% and 73.2% at 0.05 and 0.10 mg/mL, respectively, after 48 h (Figure 5Aiii).

### 3.7 Effects of *Acridocarpus smeathmannii* on the proliferation of WPMY-1 cells

EdU assays revealed dose-dependent inhibition of proliferation, with an IC_50_ of 0.0518 μg/mL at 12 h. Compared to controls, proliferation decreased by 37%, 72%, and 91% at 0.05, 0.10, and 0.25 mg/mL, respectively (Figure 5Bi). Cell counts per field decreased by 65% (0.10 mg/mL) and 78% (0.25 mg/mL) (Figure 5Bii).

### 3.8 Cumulative concentration–response curve to exogenous and endogenous stimulations

**FIGURE 6:**
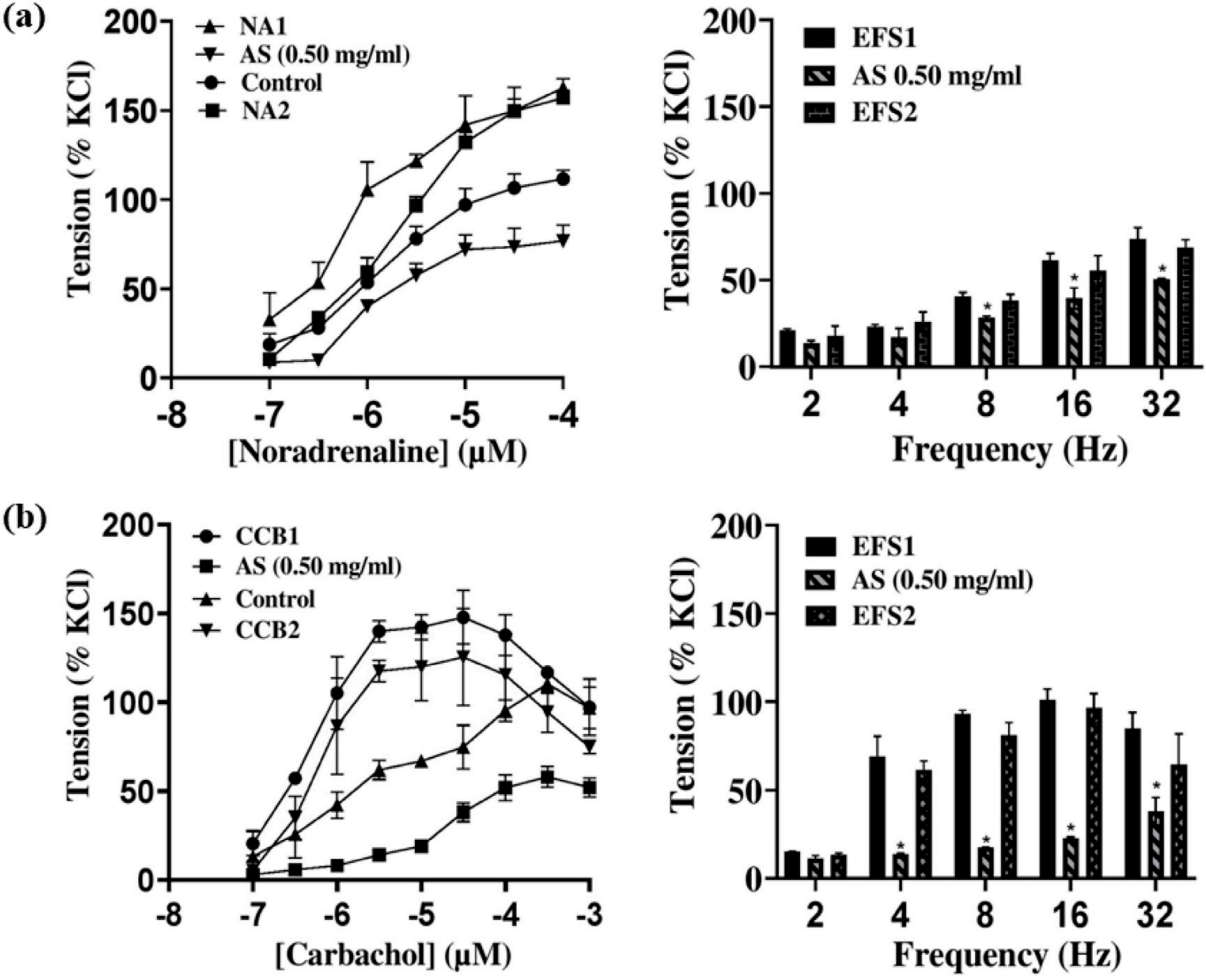
Cumulative concentration–response curves to exogenous (noradrenaline, carbachol) and endogenous (EFS) stimuli in the absence, presence, and after washout of *A. smeathmannii*. **(a)** Noradrenaline and EFS in human prostate; **(b)** carbachol and EFS in human detrusor (bladder). NA1/CCB1/EFS1: contraction without *A. smeathmannii* extract. NA2/CCB2/EFS2: Contraction after *A. smeathmannii* extract and washout. Results are expressed as mean ± SD (n = 4 patients per series, with tissue from each patient split between the *A. smeathmannii* and ethanol control groups). Tensions are expressed as a percentage of the high-molar KCl-induced contraction assessed prior to application of either the ethanol control or *A. smeathmannii*.

*Acridocarpus smeathmannii* reduced contractility elicited by exogenous (NA and CcH) and endogenous (EFS) stimuli in prostate and bladder tissues, with no significant differences pre- versus post-washout curves (Figure 6).

### 3.9 GC/MS analysis

GC-MS of the hexane extract identified the following major compounds: glutaric acid di(2-methoxybenzyl) ester (10.35%), p-cymene (5.64%), α-terpinene (4.53%), benzyl benzoate (4.44%), tau-cadinol (4.42%), amorpha-4,7(11)-diene-2-α-acetoxy (4.69%), cis-mentha-1(7),8-dien-2-ol (7.40%), pinostrobin chalcone (2.94%), stigmasterol (2.05%), γ-sitosterol (1.41%), α-patchoulene (1.95%), and 9-octadecenoic acid (5.15%), among others (Supplementary Table S1).

### 3.10 NMR spectroscopy

**FIGURE 7:**
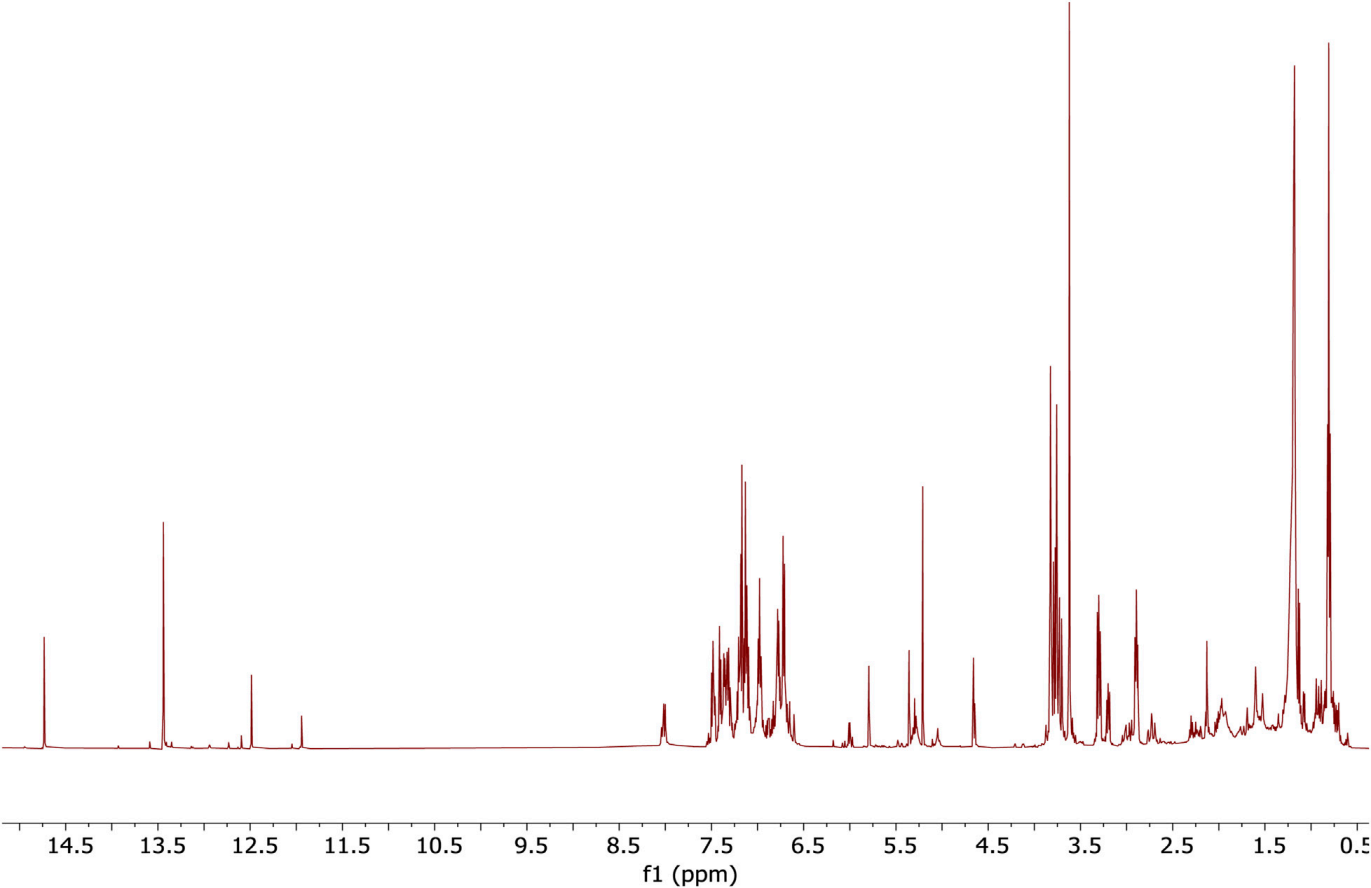
^1^H NMR analysis of the hexane extract of *Acridocarpus smeathmannii* (DC.) Guill. and Perr. root.

The ^1^H NMR spectrum of the hexane extract downfield-shifted singlet signals >11.5 ppm suggested the presence of carboxylic acid moieties (R–COOH) and hydrogen-bonded OH protons (R–OH) (e.g., phenolic moieties); strong signals between 7.0 and 8.0 ppm reflected the presence of several aromatic systems, possibly from molecules containing multiple benzene or heteroaromatic rings. Signals in the 3.5–4.0 ppm region suggested alcohol (HO–CH_x_–), ether (–O–CH_x_–), or ester functional groups, and signals below 3.0 ppm indicated aliphatic chains indicative of branched methylene and methyl groups (Figure 7).

## 4 Discussion

In recent years, extracts from various medicinal plants have been investigated for their potential to improve LUTS associated with BPH, in both preclinical studies and clinical trials (Tamalunas et al., 2022; Adler et al., 2012; Brookman-May et al., 2019; Stewart and Lephart, 2023; Kale et al., 2025).

The effect of *A. smeathmannii* on smooth muscle contractility in isolated human tissue preparations has not been studied. We, therefore, assessed the inhibitory effects of *A. smeathmannii* on adrenergic and cholinergic contractions in human prostate and bladder tissues. Concentration-dependent responses to adrenergic agonists (adrenaline and phenylephrine; Figure 1), cholinergic agonists (carbachol and methacholine; Figure 2), and neurogenic stimulations by EFS were evaluated using different concentrations of *A. smeathmannii*. Additionally, the anticontractile actions of *A. smeathmannii* were examined in porcine coronary and interlobar arteries (Figures 3, 4). Parallel experiments assessed the effects of *A. smeathmannii* on growth-related functions in cultured prostate stromal cells (Figure 5).

The clinical management of LUTS with currently available drugs is limited (Lerner et al., 2021). Both OAB and BPH are largely influenced by the physiology and pharmacology of prostate and bladder smooth muscle, particularly concerning adrenergic and cholinergic systems. Whereas voiding symptoms and BPH respond to α-adrenoceptor antagonists and 5α-reductase inhibitors, β-adrenoceptor agonists and muscarinic receptor antagonists are commonly used for storage symptoms/OAB (Trajanoska et al., 2023). Evidence indicates that both BPH and OAB can coexist in patients with LUTS. However, an ideal pharmacological agent capable of simultaneously treating both voiding and storage symptoms is lacking. The resulting polypharmacy may lead to poor compliance and adverse drug reactions. These limitations present opportunities for phytotherapeutic agents that may offer comprehensive management of LUTS suggestive of BPH. *Acridocarpus smeathmannii* extract could potentially combine the benefits of current combination therapies, improving the management of both OAB and BPH.

Recent overviews of pharmacotherapeutic options and essential strategies for managing LUTS/BPH have been updated (Takeuchi et al., 2023; Koudonas et al., 2023; Gravas et al., 2023). Although these agents have provided symptomatic relief not exceeding 50%, our results show that *A. smeathmannii* exerts anticontractile effects on smooth muscle contractions, mimicking adrenergic and cholinergic inhibition, as well as EFS-induced contractions *in vitro*. This positions *A. smeathmannii* as a potential candidate for treating mixed LUTS. The observed degree of inhibition parallels the effects seen with α_1_-blockers in *in vitro* EFS-induced contractions. These findings may support the use of *A. smeathmannii* in traditional and alternative medicine not only for voiding symptoms but also for treating disorders related to vasocontraction, such as erectile dysfunction, which often coexists with LUTS.

Mechanistically, *A. smeathmannii* extract demonstrated inhibitory effects on adrenergic stimulations with both NA and PHE, with greater consistency in PHE-induced contractions (Figures 1, 2). These effects were noncompetitive and associated with a decreased E_max_ of contractions induced by either agonists or EFS, without significant changes in EC_50_ values. Furthermore, *A. smeathmannii* extract inhibited cholinergic contractions of the bladder induced by CcH, McH, and EFS. Relaxation of a bladder tone is a desirable strategy in managing storage symptoms of OAB (Beland et al., 2022). These findings support the potential of *A. smeathmannii* in targeting both adrenergic and cholinergic pathways, and reinforce it to be promising for urological conditions.

Investigations in porcine coronary and interlobar arteries further elucidated the potential effects of *A. smeathmannii* extract. The extract slightly inhibited CcH- and EFS-induced contractions in porcine coronary arteries. Although both prostatic and vascular smooth muscle share adrenergic mechanisms, differences in post-receptor pathways and α_1_-adrenergic receptor subtypes may explain varied responses (De Luna Alves et al., 2024). In the porcine interlobar artery, *A. smeathmannii* showed inhibitory effects on NA-induced contractions. Endothelium-dependent and -independent mechanisms may also underlie these actions, suggesting possible neurohumoral involvement and novel cellular interactions. These findings provide support for the ethnomedicinal use of *A. smeathmannii*. However, the exact mechanisms by which *A. smeathmannii* regulates neurotransmitter release remain unclear, requiring further investigation.

As animal study replacement gains traction in pharmacology (Hankenson et al., 2024), cell-based assays are increasingly used to evaluate efficacy and safety. In this study, we used the CCK-8 assay to assess the viability and cytotoxicity of *A. smeathmannii* extract on immortalized WPMY-1 cells. Compared to MTT, CCK-8 offers improved sensitivity, reduced interference from pigmented phytochemicals, and better performance at low cell densities (Khalef et al., 2024). Our results showed a time-dependent inhibitory effect of *A. smeathmannii* extract on WPMY-1 cells (Figure 5Ai–iii), suggesting lower dosing requirements with prolonged exposure and potentially fewer side effects. The EdU assay confirmed concentration-dependent inhibition of cell proliferation by *A. smeathmannii* (Figure 5Bi,ii). This inhibitory effect on cultured stromal cell growth parallels the action of 5α-reductase inhibitors used in BPH management. Given the low tolerability of 5α-reductase inhibitors, plant extracts like *A. smeathmannii* with better safety profiles may represent promising alternatives. This study is the first to demonstrate the smooth muscle relaxant potential of *A. smeathmannii*. More importantly, our findings align with previous reports suggesting that members of this plant family may contain cytotoxic compounds capable of modulating multiple cell death pathways (Cao et al., 2004; Balhamar et al., 2019; Jamshidi-Adegani et al., 2020).

**FIGURE 8:**
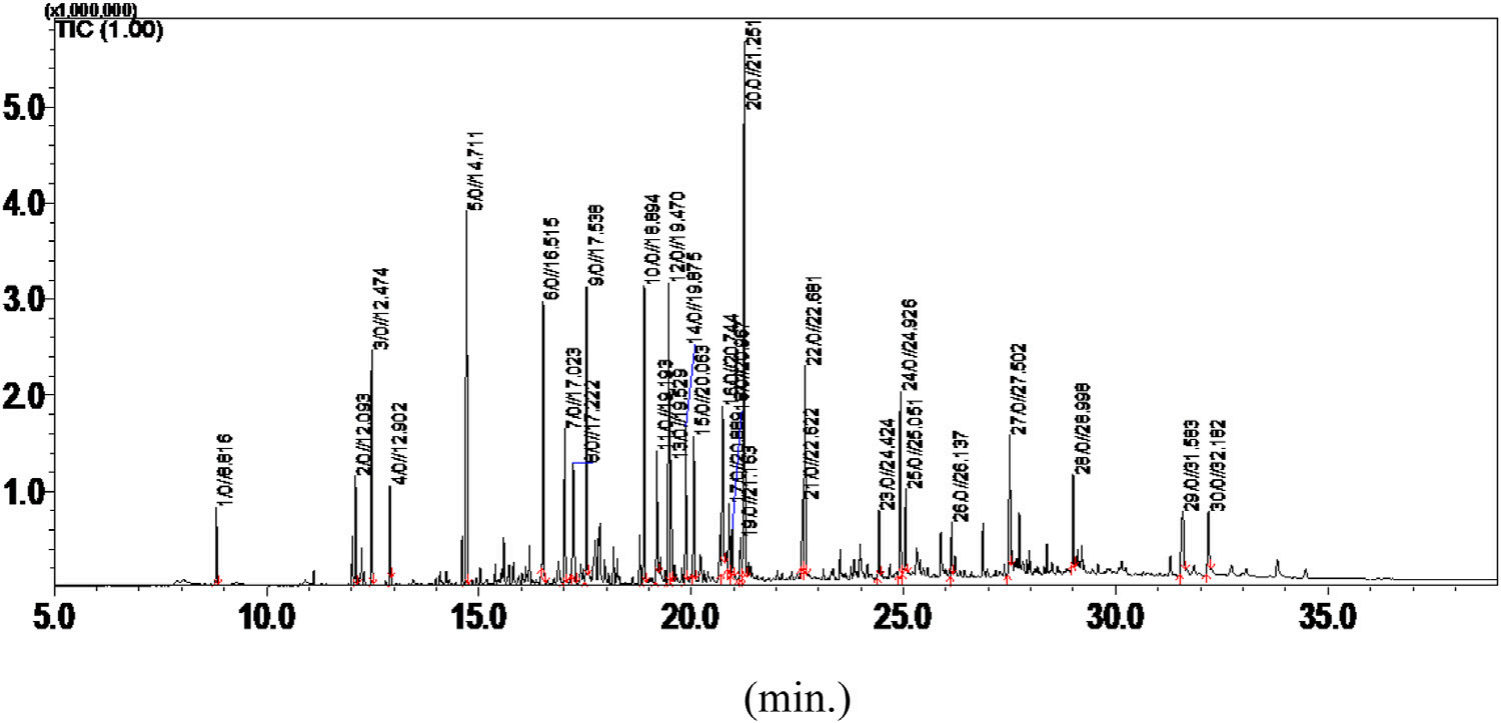
GC/MS analysis of the hexane extract of *Acridocarpus smeathmannii* (DC.) Guill. and Perr. root.

Preliminary GC-MS analysis (Figure 8; Table 4) identified sesquiterpene hydrocarbons and phytosterols such as stigmasterol, γ-sitosterol, α-pinostrobin chalcone, α-patchoulene, γ-terpinene, thymol methyl ether, p-cymene, benzyl benzoate, and tau-cadinol. Other compounds included fatty acids and antioxidant agents such as glutaric acid di(2-methoxybenzyl) ester and l-(+)-ascorbic acid 2,6-dihexadecanoate (Johra et al., 2023). These compounds are known for their anti-inflammatory and antioxidant properties, which confirm the ^1^H-NMR spectrum of the crude extract (Figure 8). The diverse composition of *A. smeathmannii* may contribute to synergistic antioxidant effects, potentially supporting its use in complementary and alternative medicine (Guo et al., 2021).

**TABLE 4:** Bioactive compounds in *Acridocarpus smeathmannii* root extract and their binding affinities.

S,N	Compound name	PubChem CID	Molecular formula	Molecular weight (g/mol)	Binding affinity vs. α_1_ receptor (kcal/mol)	RMSD/ub	RMSD/lb
1	2-α-acetoxyamorpha-4,7 (11)-diene	91,752,529	C_17_H_26_O_2_	262.40	−6.9	0	0
2	p-Cymene	7,463	C_10_H_14_	134.22	−5.0	0	0
3	Guaiol	227,829	C_15_H_26_O	222.37	−6.2	0	0
4	tau-Cadinol	160,799	C_15_H_26_O	222.37	−6.1	0	0
5	Benzyl benzoate	2,345	C_14_H_12_O_2_	212.24	−5.8	0	0
6	α-Patchoulene	521,710	C_15_H_24_	204.35	−6.1	0	0
7	γ-Terpinene	7,461	C_10_H_16_	136.23	−5.0	0	0
8	l-(+)-Ascorbic acid 2,6-dihexadecanoate	54,722,209	C_38_H_68_O_8_	652.90	−7.3	0	0
9	Thymol, methyl ether	14,104	C_11_H_16_O	164.24	−4.9	0	0
10	Benzyl oleate, Octadecanoic acid	5,368,218	C_25_H_40_O_2_	372.60	−5.2	0	0
11	1,4-Methanoazulene, 7-bromodecahydro-4,8,8-trimethyl-9-methylene	608,959	C_15_H_23_Br	283.25	−6.3	0	0
12	2-Methoxybenzyl alcohol	69,154	C_8_H_10_O_2_	138.16	−4.6	0	0
13	Cyclohexene, 3,4-diethenyl-1,6-dimethyl-l	564,668	C_12_H_18_	162.27	−5.2	0	0
14	9-Hexadecenoic acid	5,282,745	C_16_H_30_O_2_	254.41	−4.6	0	0
15	Pinostrobin chalcone	5,316,793	C_16_H_14_O_4_	270.28	−6.1	0	0
16	Glutaric acid, di(2-methoxybenzyl) ester	91,715,745	C_21_H_24_O_6_	372.40	−6.4	0	0
17	cis-α-Necrodyl acetate	91,748,886	C_12_H_20_O_2_	196.29	−5.8	0	0
18	Stigmasterol	5,280,794	C_29_H_48_O	412.70	−7.8	0	0
19	γ-Sitosterol	457,801	C_29_H_50_O	414.70	−7.1	0	0
20	2,5-Cyclohexadiene, 1,4-diethyl-1,4-dimethyl-	572,347	C_12_H_20_	164.29	−5.1	0	0
21	Tamsulosin	129211	C_20_H_28_N_2_O_5_S	408.50	−8.2	0	0

Our findings show that *A. smeathmannii* inhibits contractile responses to both exogenous and endogenous stimuli. Its ability to reduce cell proliferation and viability further supports its therapeutic potential. Concentration–response curves constructed using the highest dose (0.50 mg/mL) showed consistent inhibition of contractions elicited by noradrenaline, carbachol, and EFS (Figure 6). However, no significant differences were found between pre- and post-treatment contractions, supporting the relative safety of the extract at moderate doses (Kale et al., 2019c).

*In silico* absorption, distribution, metabolism, excretion, and toxicity (ADMET) and molecular docking studies further evaluated the drug metabolism and pharmacokinetics (DMPK) properties of *A. smeathmannii* compounds. Several bioactive compounds demonstrated strong binding affinities to the α_1_-adrenergic receptor, comparable to tamsulosin. Stigmasterol and α-pinostrobin chalcone showed the highest binding affinities and protein interactions (Huang et al., 2024). ADMET predictions using SwissADME and pkCSM suggested good lipophilicity and gastrointestinal absorption for major compounds, including glutaric acid esters, γ-sitosterol, and tau-cadinol. Most compounds were not substrates of P-glycoprotein; however, some inhibited P-glycoprotein I and II, with overall favorable safety profiles.

This study had certain limitations. It was experimental in design and did not involve patient recruitment. Normal tissues were obtained from the transitional periurethral zones of patients undergoing radical prostatectomy or cystectomy, without BPH diagnoses. A tissue was split evenly between treatment and control groups, ensuring consistent comparison of contractile responses.

## 5 Conclusion

This study reports for the first time that *A. smeathmannii* extract inhibits α_1_-adrenergic and cholinergic contractions in the prostate, bladder, and porcine arteries, with effects comparable to those of α_1_-blockers and anticholinergics. In addition, *in silico* studies revealed that stigmasterol, pinostrobin chalcone, γ-sitosterol, and l-(+)-ascorbic acid 2,6-dihexadecanoate showed the highest binding affinity to the α_1_-adrenergic receptor. These findings validate its ethnobotanical use and support further investigation of its bioactive compounds for therapeutic development.

## Data Availability

The original contributions presented in the study are included in the article/Supplementary Material; further inquiries can be directed to the corresponding author.

## Abbreviations

AS, *Acridocarpus smeathmannii*; ATM, African traditional medicine; ADMET, absorption, distribution, metabolism, excretion, and toxicity; BPH, benign prostatic hyperplasia; CcH, carbachol; CCK-8, Cell Counting Kit-8; DMPK, drug metabolism and pharmacokinetics; EFS, electrical field stimulation; GC-MS, gas chromatography–mass spectrometry; KH, Krebs–Henseleit; LUTSs, lower urinary tract symptoms; EC_50_, maximum agonist-induced contraction; E_max_, maximum possible contraction; McH, methacholine; NMR, nuclear magnetic resonance; NA, noradrenaline; PHE, phenylephrine; rCx, radical cystectomy; rPx, radical prostatectomy; f, frequency; Ef_50_, maximum EFS-induced contraction; MD, mean difference; EdU, 5-ethynyl-2ʹ-deoxyuridine.
