# Supplementary material for: Acridocarpus smeathmannii root extracts inhibit human prostate and bladder smooth muscle contraction, porcine arterial vasoconstriction, and cytotoxicity of prostate stromal cells
Source: Front Pharmacol. 2025 Jul 22;16:1621346. doi: 10.3389/fphar.2025.1621346 (PMC12321789; doi:10.3389/fphar.2025.1621346)
Supplement: Supplementary file 1 [file Table1.docx]

Supplementary table 1. GC/MS analysis of the hexane extract of *A. smeathmannii* (DC.) Guill. & Perr. root.

| Retention Time (min.) | Peak Area% | Name |
| --- | --- | --- |
| 8.817 | 0.97 | Benzyl alcohol |
| 12.093 | 1.95 | Thymol, methyl ether |
| 12.473 | 3.17 | 2-Methoxybenzyl alcohol |
| 12.900 | 1.34 | Bornyl acetate |
| 14.710 | 5.64 | p-Cymene |
| 15.583 | 0.67 | Phenol, 3-(1,1-dimethylethyl)-4-methoxy- |
| 16.513 | 4.69 | 2-α-Acetoxyamorpha-4,7(11)-diene |
| 17.023 | 2.02 | Guaiol |
| 17.220 | 2.28 | β-Copaen-4-alpha-ol |
| 17.537 | 4.42 | tau-Cadinol |
| 17.740 | 0.67 | Selina-6-en-4-ol |
| 17.850 | 0.81 | 5-Azulenemethanol, 1,2,3,3a,4,5,6,7-octahydro-.alpha.,.alpha.,3,8-tetramethyl- |
| 18.893 | 4.44 | Benzyl Benzoate |
| 19.193 | 1.95 | α-Patchoulene |
| 19.470 | 4.53 | γ-Terpinene |
| 19.530 | 1.64 | Cyclohexene, 3,4-diethenyl-1,6-dimethyl- |
| 19.873 | 2.15 | 2,5-Cyclohexadiene, 1,4-diethyl-1,4-dimethyl- |
| 20.063 | 2.02 | 1,3-Cyclohexadiene, 1-methyl-4-(1-methylethyl)- |
| 20.677 | 0.67 | 8a-Methyl-5-methylene-3-([(pyridin-3-ylmethyl)-amino]-methyl)-decahydro-naphtho[2,3-b]furan-2-one |
| 20.743 | 3.33 | l-(+)-Ascorbic acid 2,6-dihexadecanoate |
| 21.163 | 0.77 | trans-β-Terpinyl benzoate |
| 21.250 | 10.35 | Glutaric acid, di(2-methoxybenzyl) ester |
| 22.623 | 1.35 | Octadecanoic acid |
| 24.423 | 0.97 | 1,4-Methanoazulene, 7-bromodecahydro-4,8,8-trimethyl-9-methylene- |
| 24.927 | 2.94 | 2-Propen-1-one, 1-(2,6-dihydroxy-4-methoxyphenyl)-3-phenyl-, (E)- |
| 26.137 | 0.74 | Dodecanoic acid, phenylmethyl ester |
| 27.503 | 2.51 | 9-Hexadecenoic acid, phenylmethyl ester, (Z)- |
| 28.997 | 1.45 | cis-α-Necrodyl acetate |
| 31.583 | 2.05 | Stigmasterol |
| 32.183 | 1.41 | α-sitosterol |


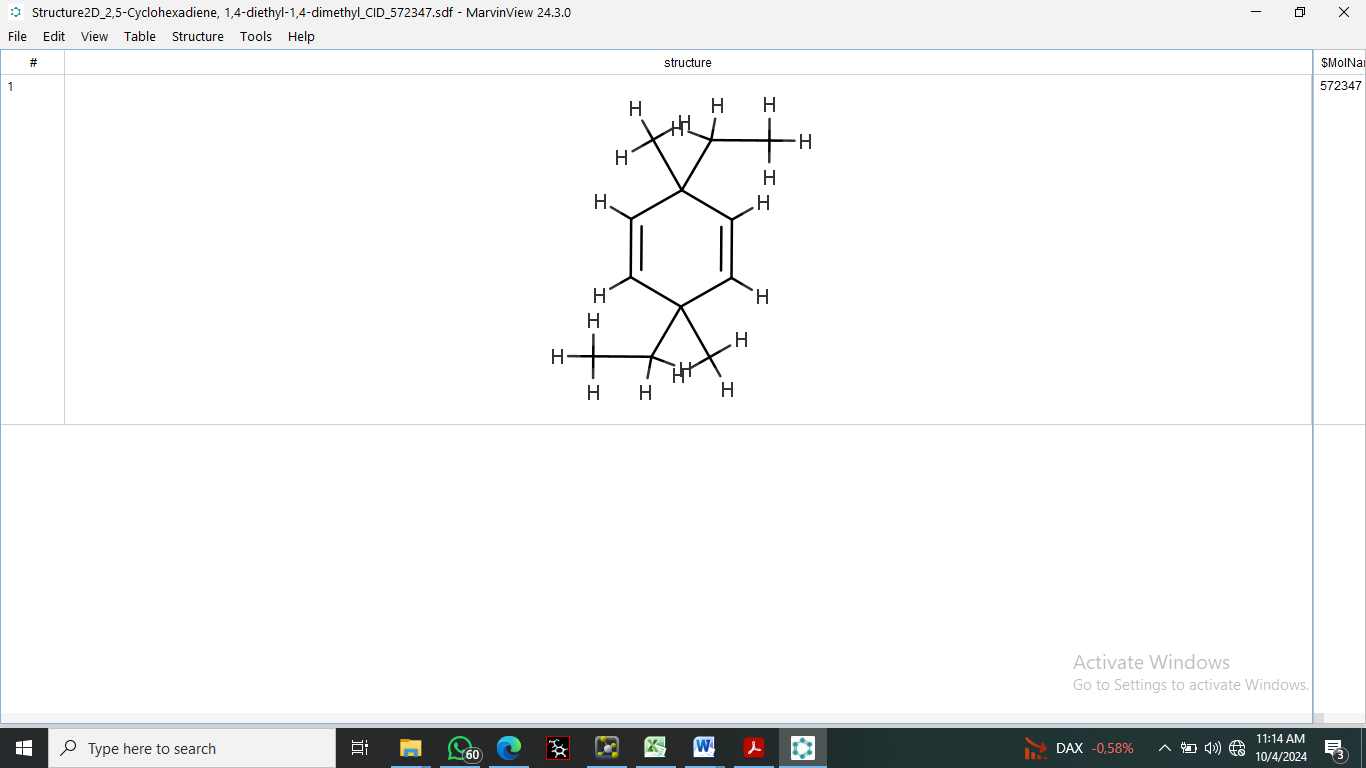

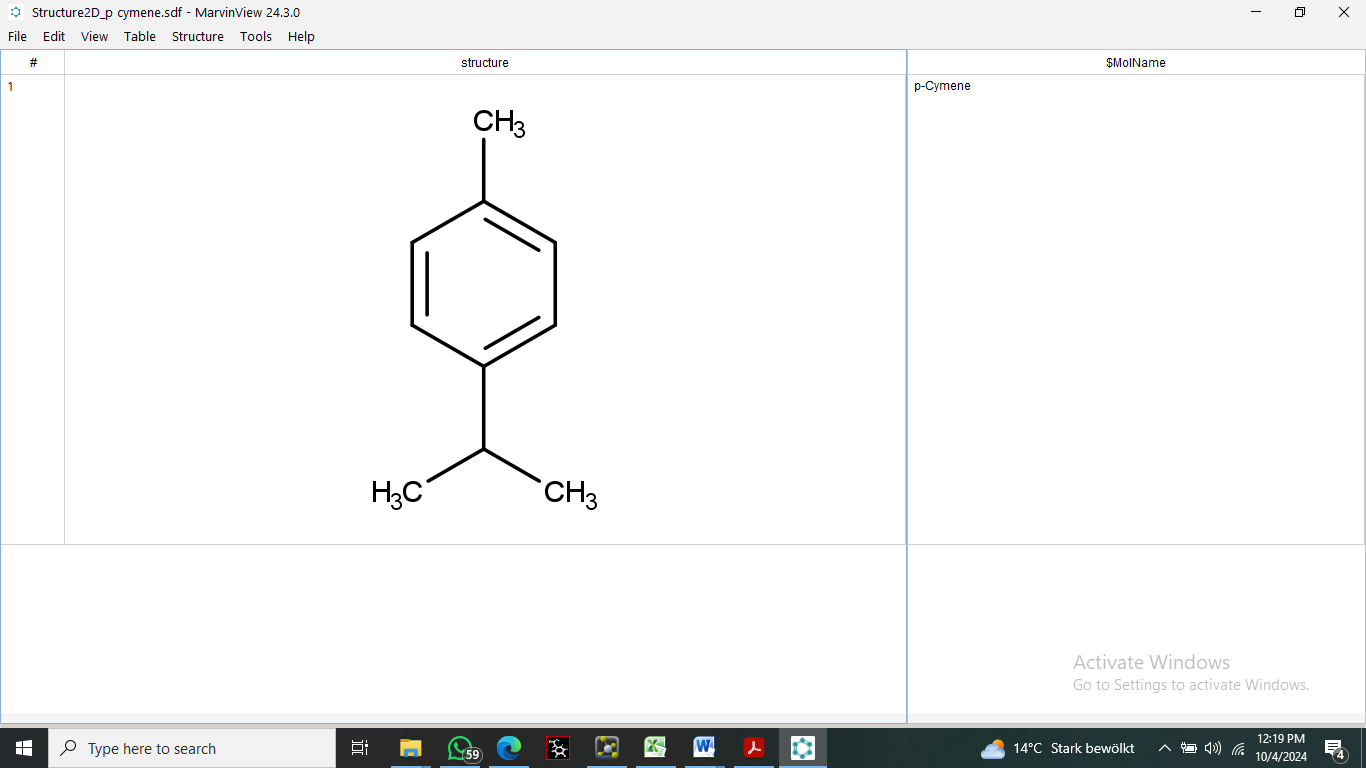

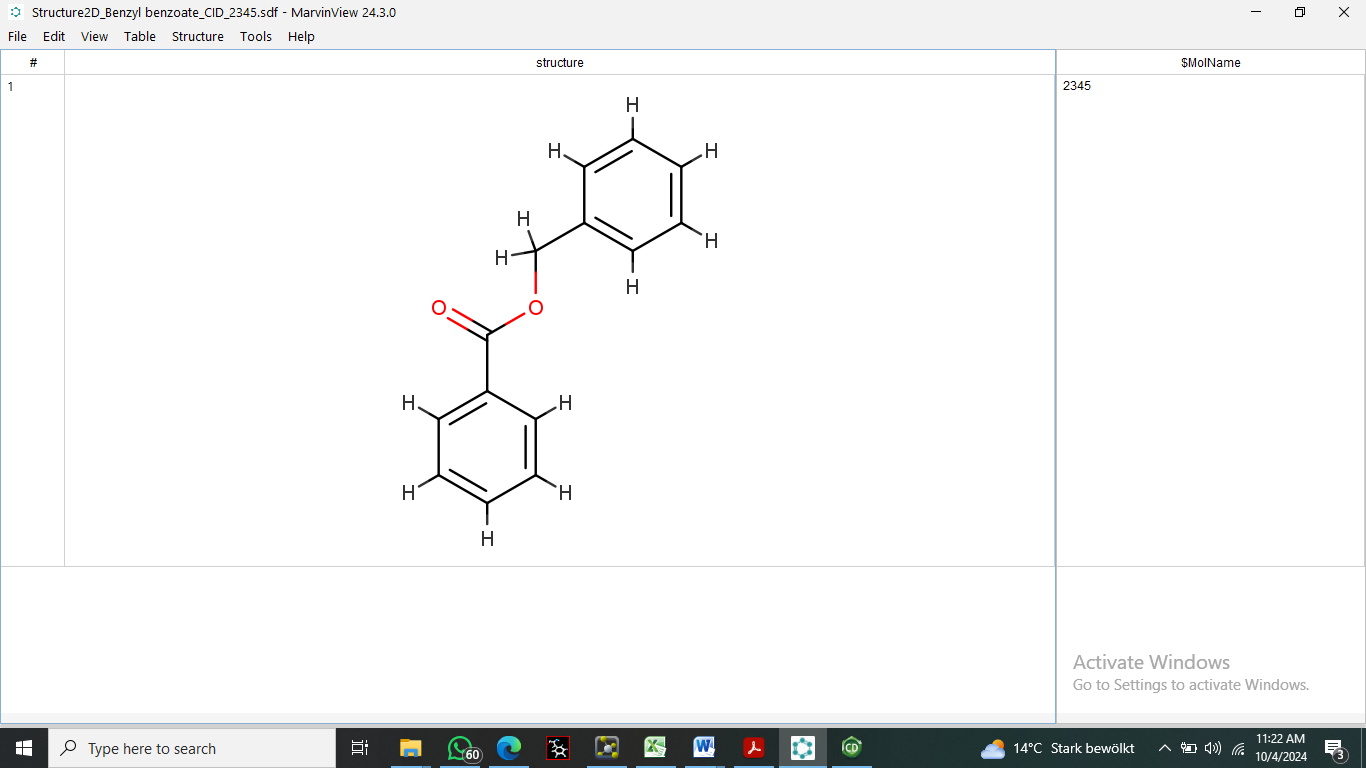

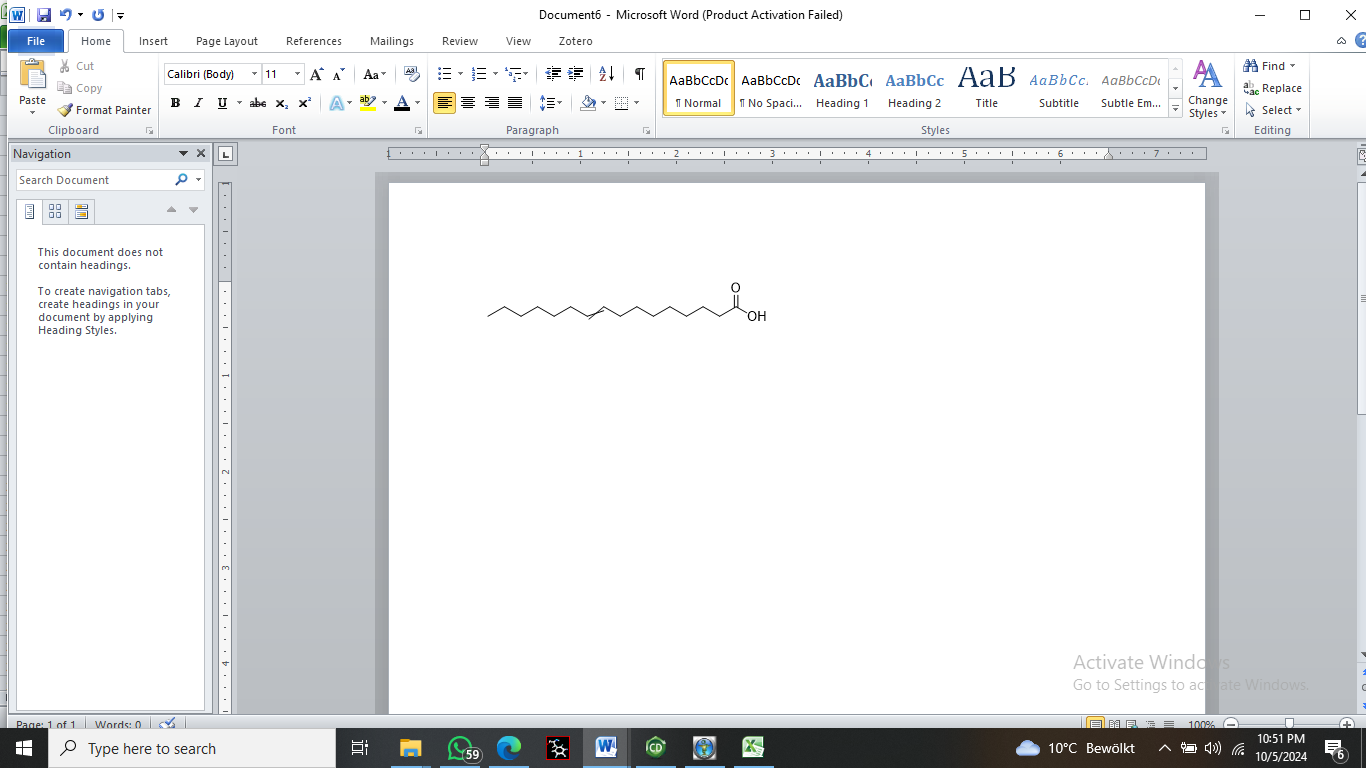


2,5-Cyclohexadiene, 1,4-diethyl-1,4-dimethyl

P-cymene

9-Hexadecenoic acid

Benzyl Benzoate

2-Methoxybenzyl alcohol


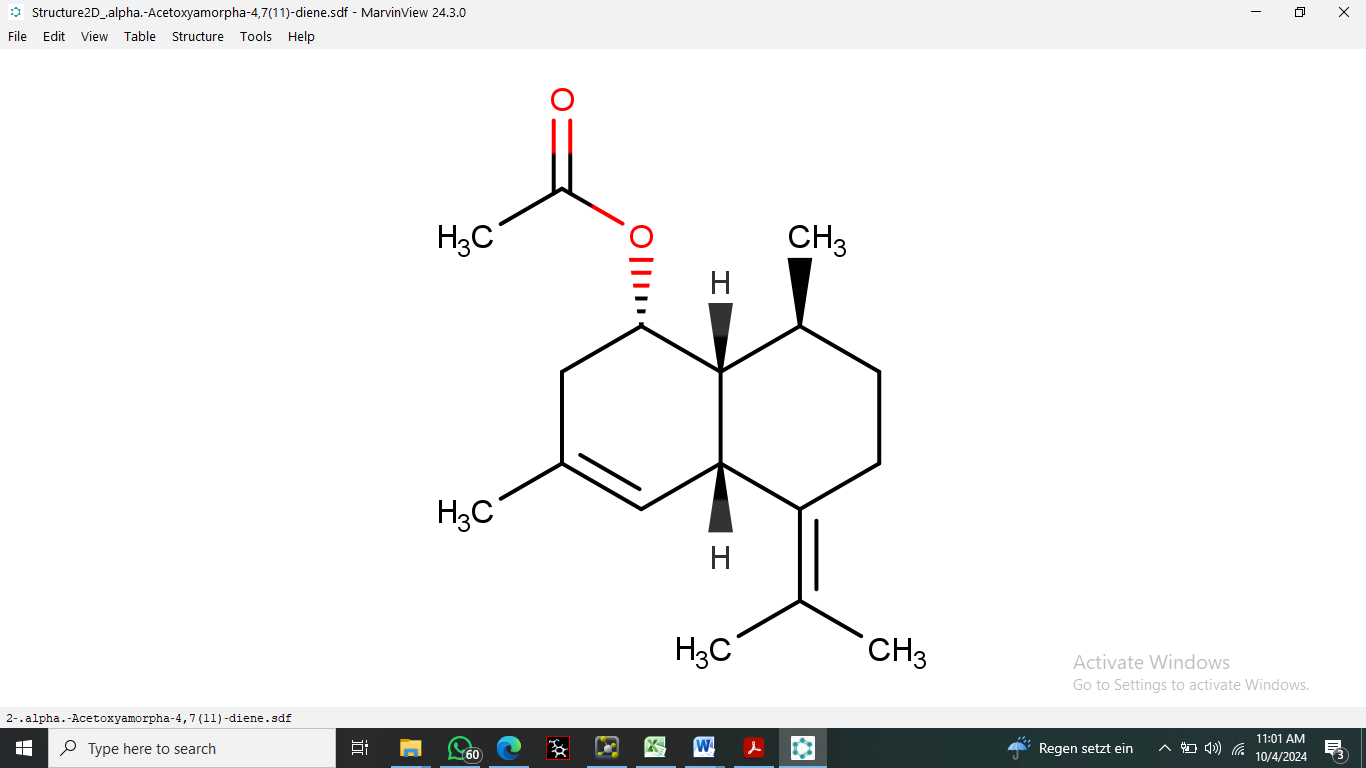

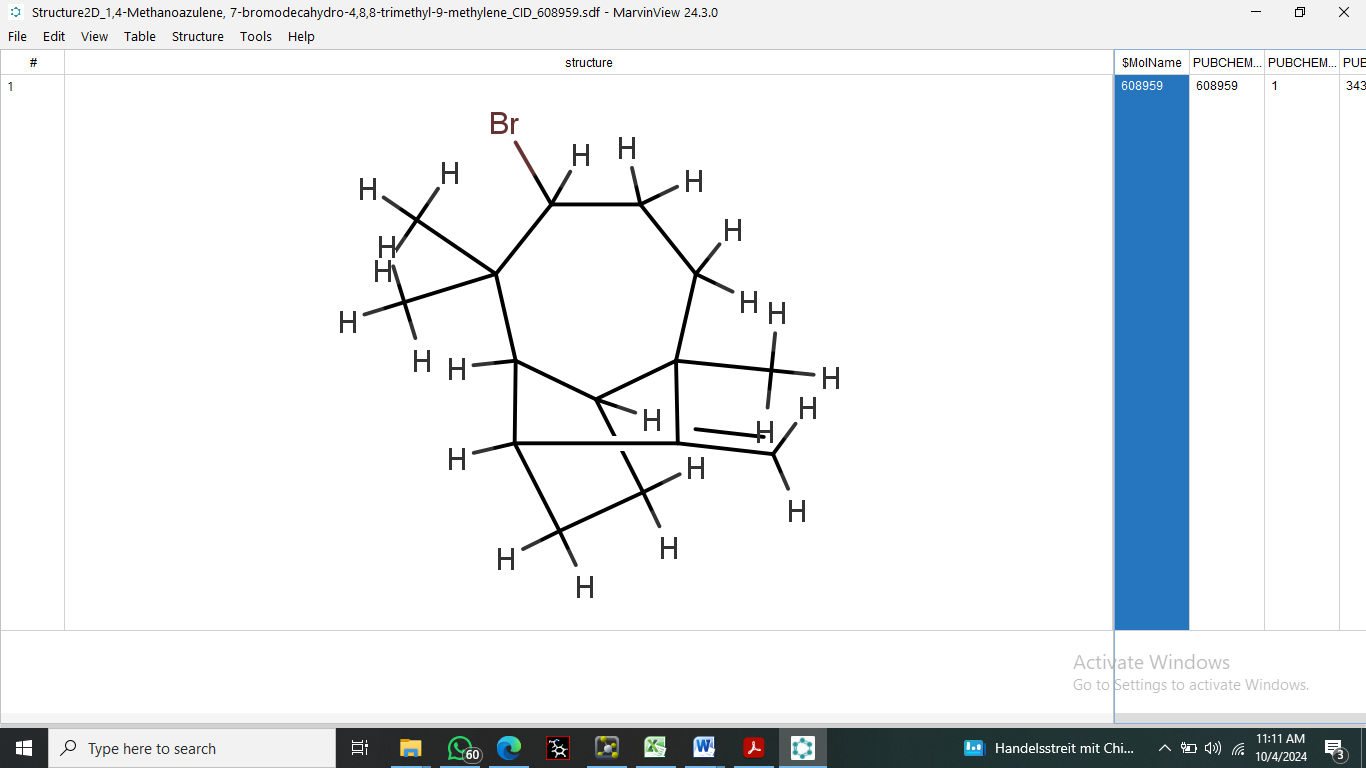

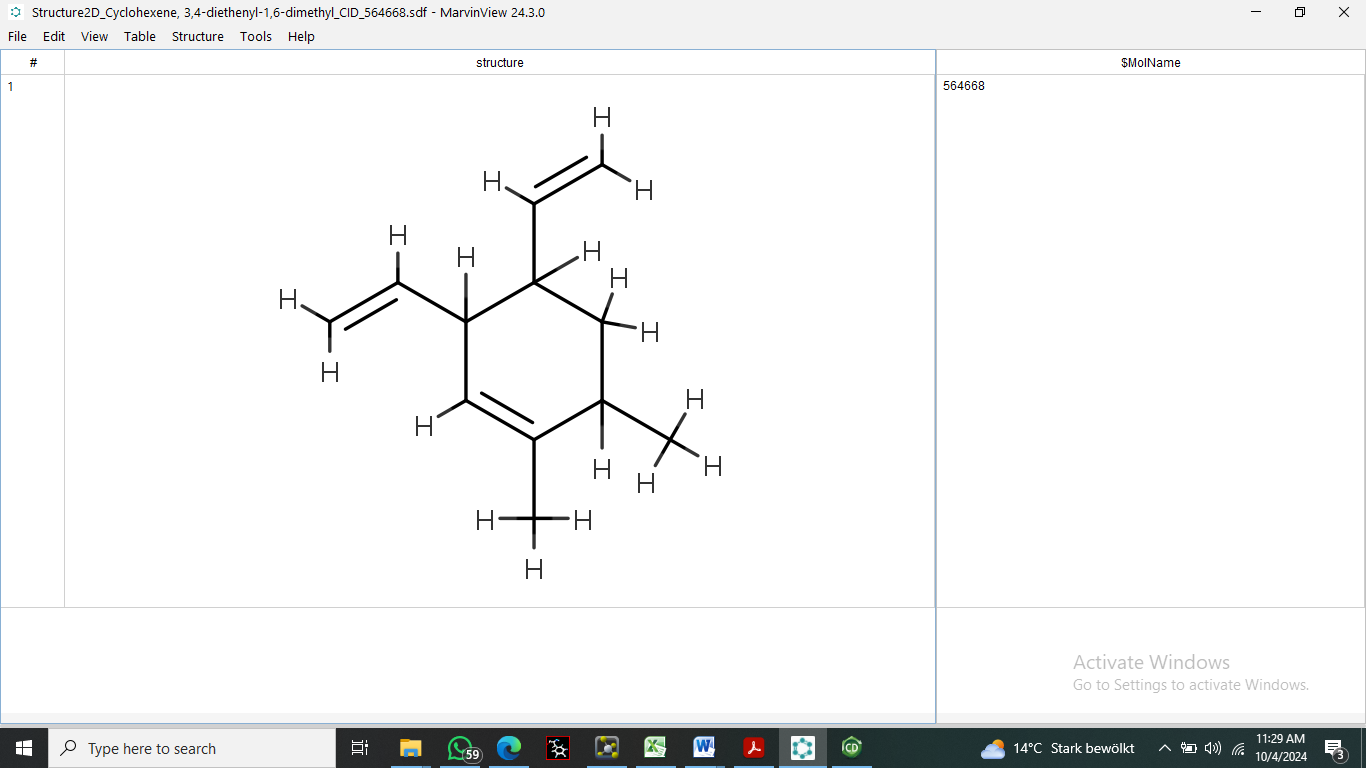

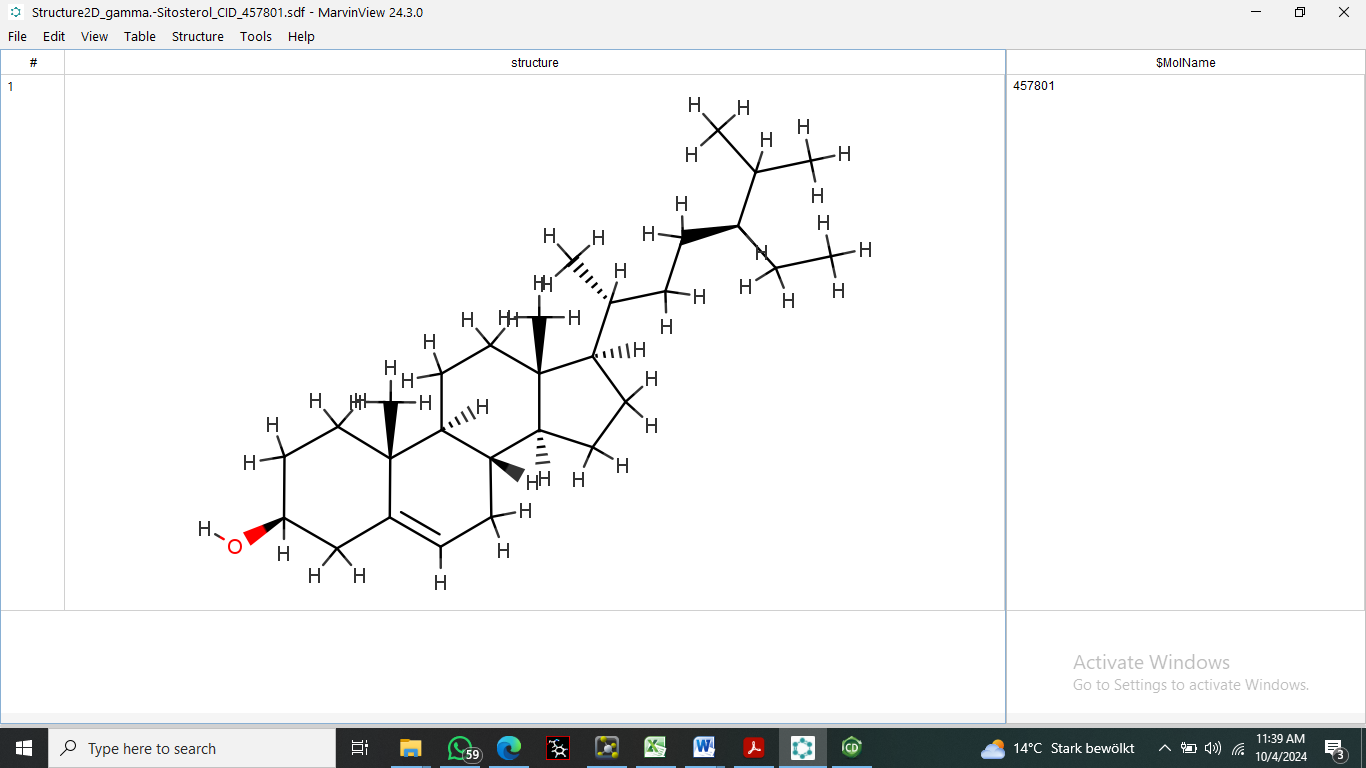

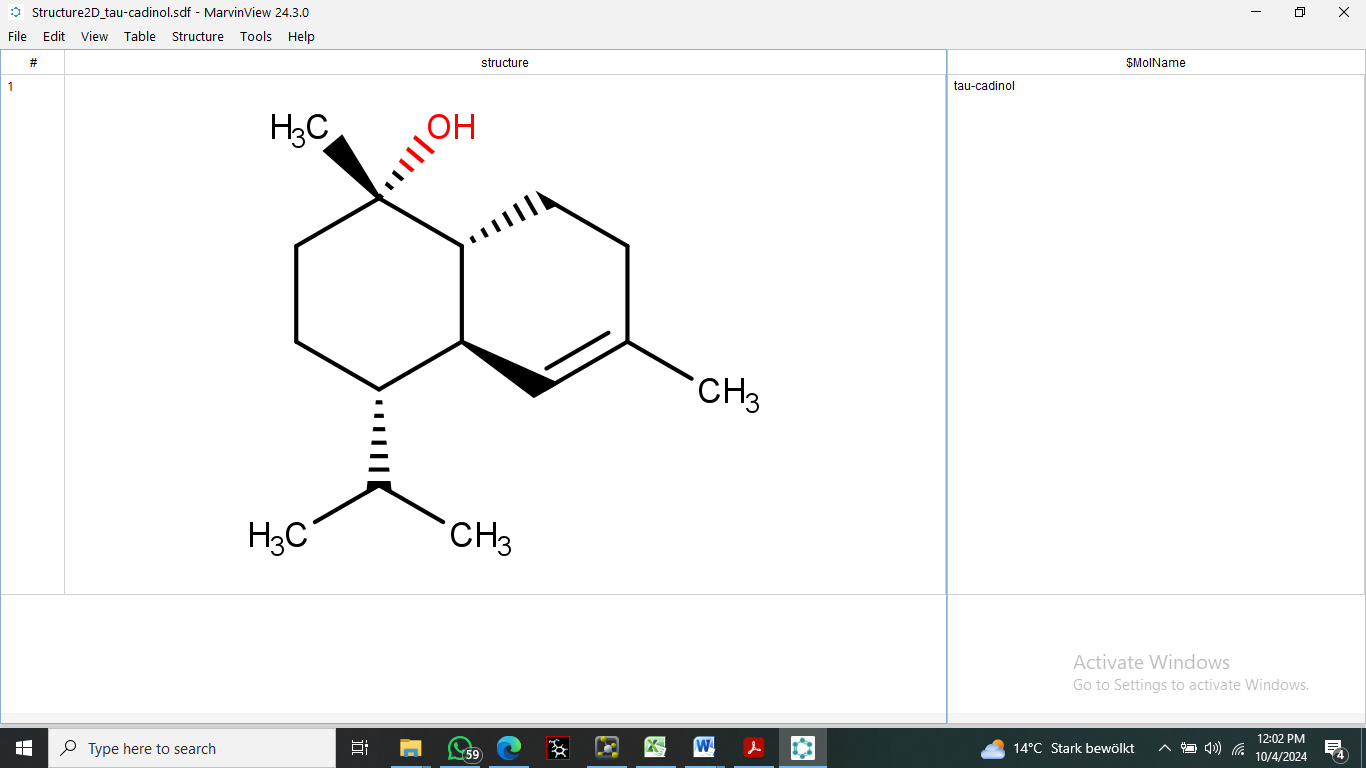


2.α.-Acetoxyamorpha-4,7(11)-diene

tau-Cadinol

γ-sitosterol

1,6-Dimethyl-3,4-divinyl-1-cyclohexene

1,4-Methanoazulene


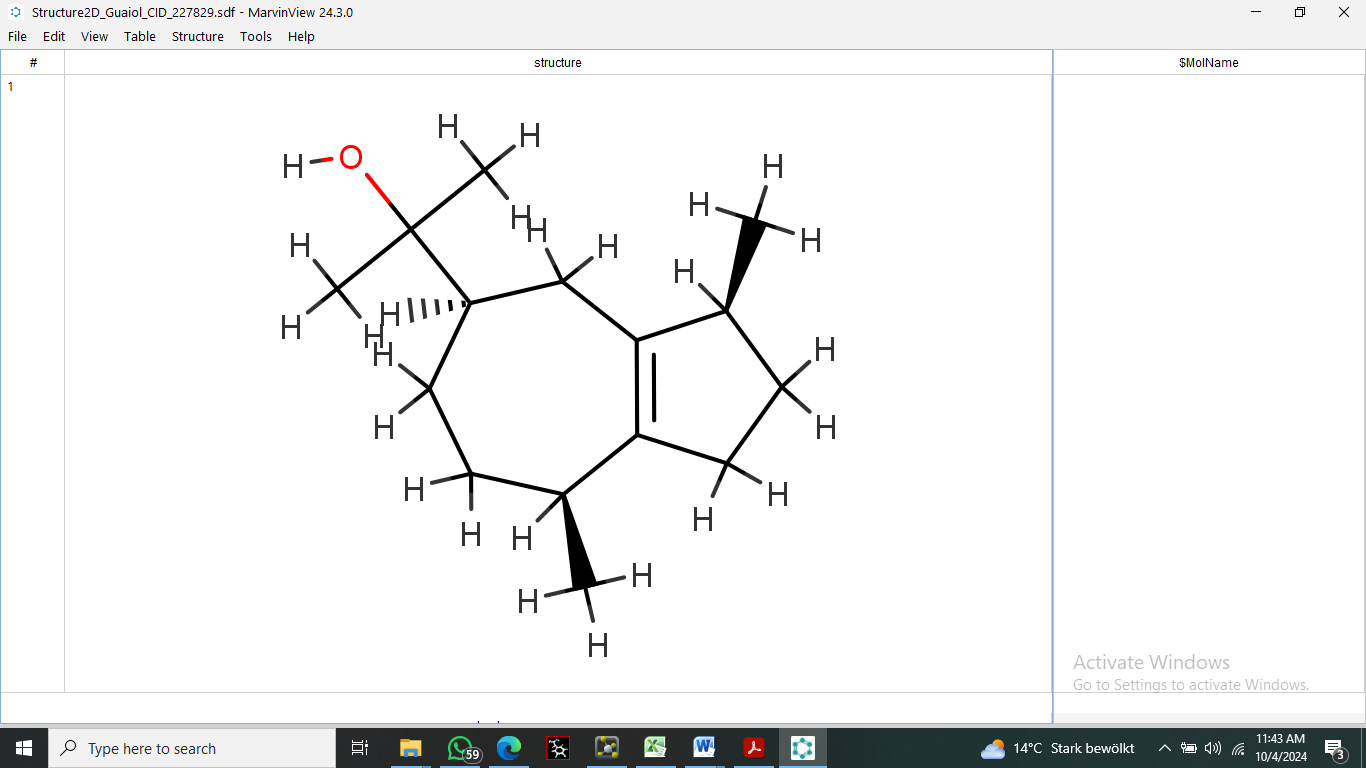

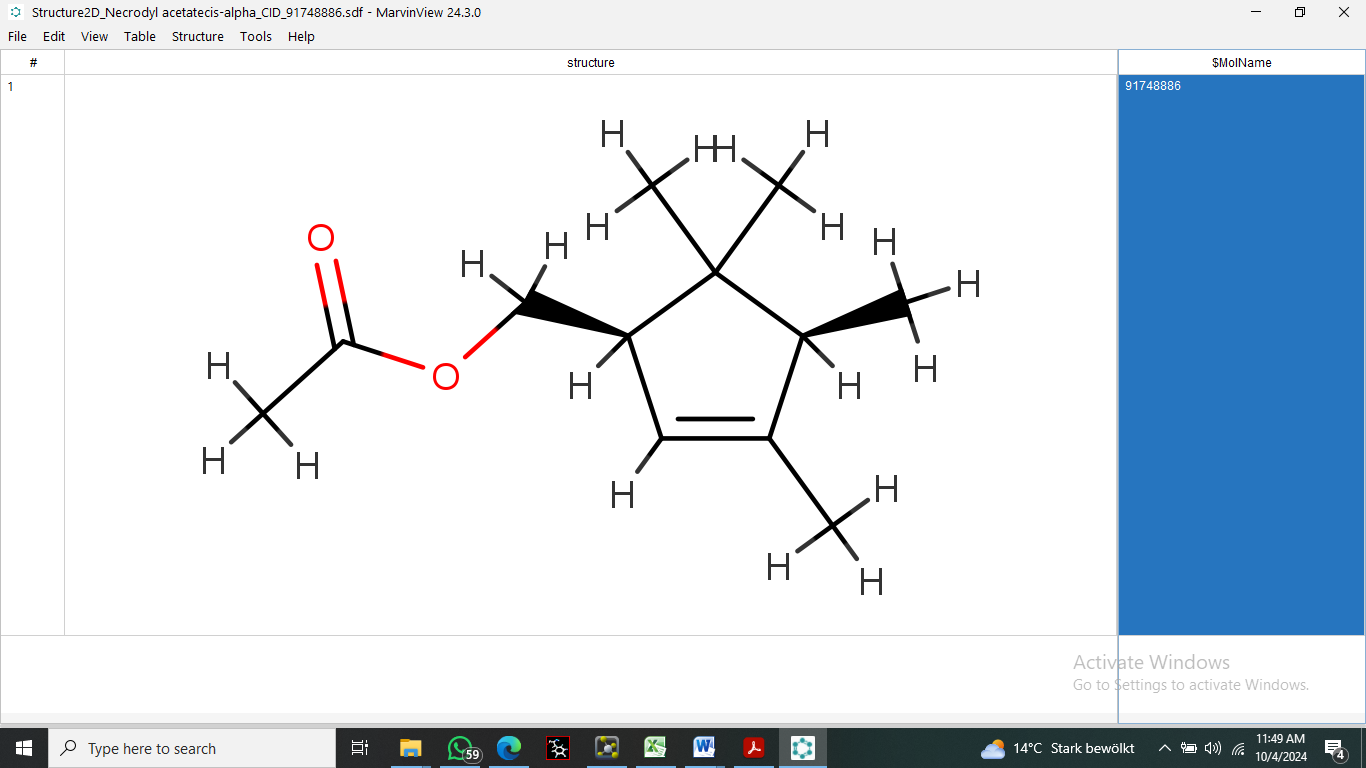

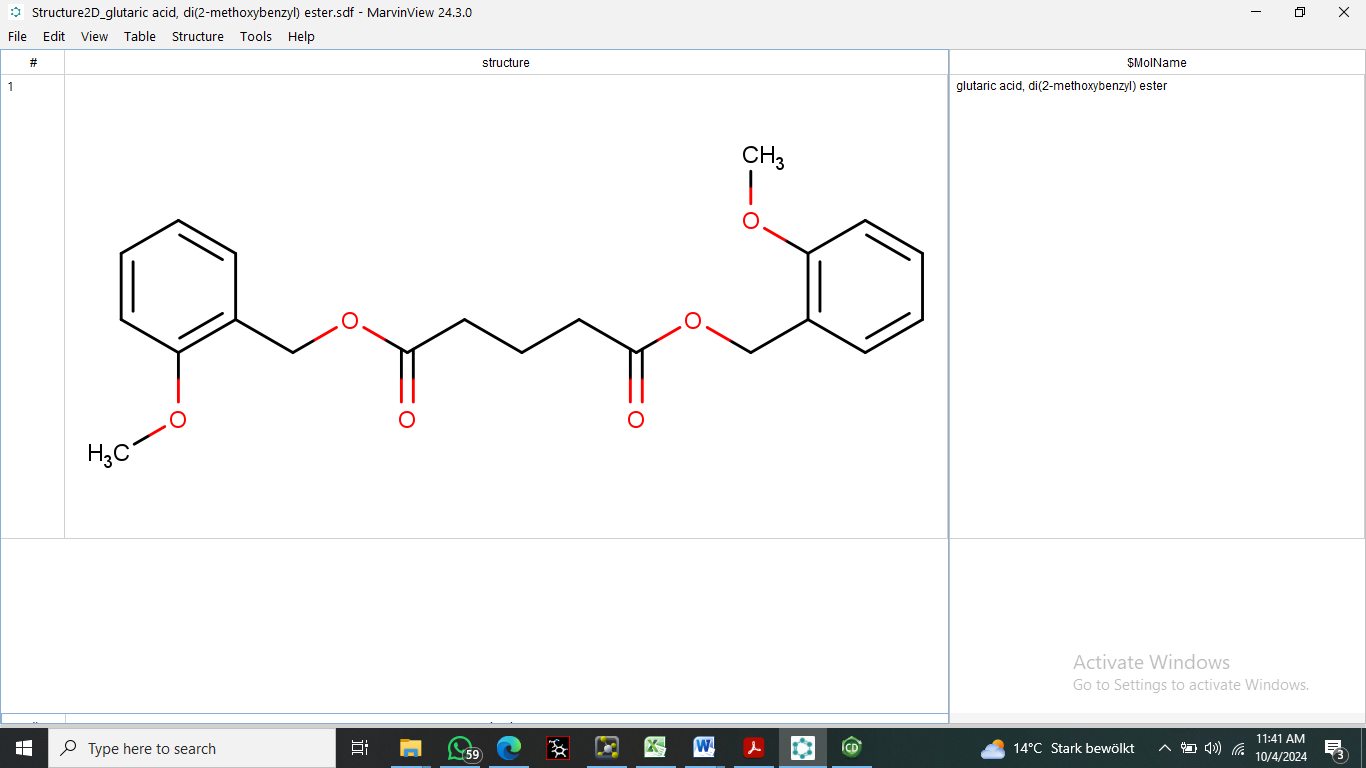

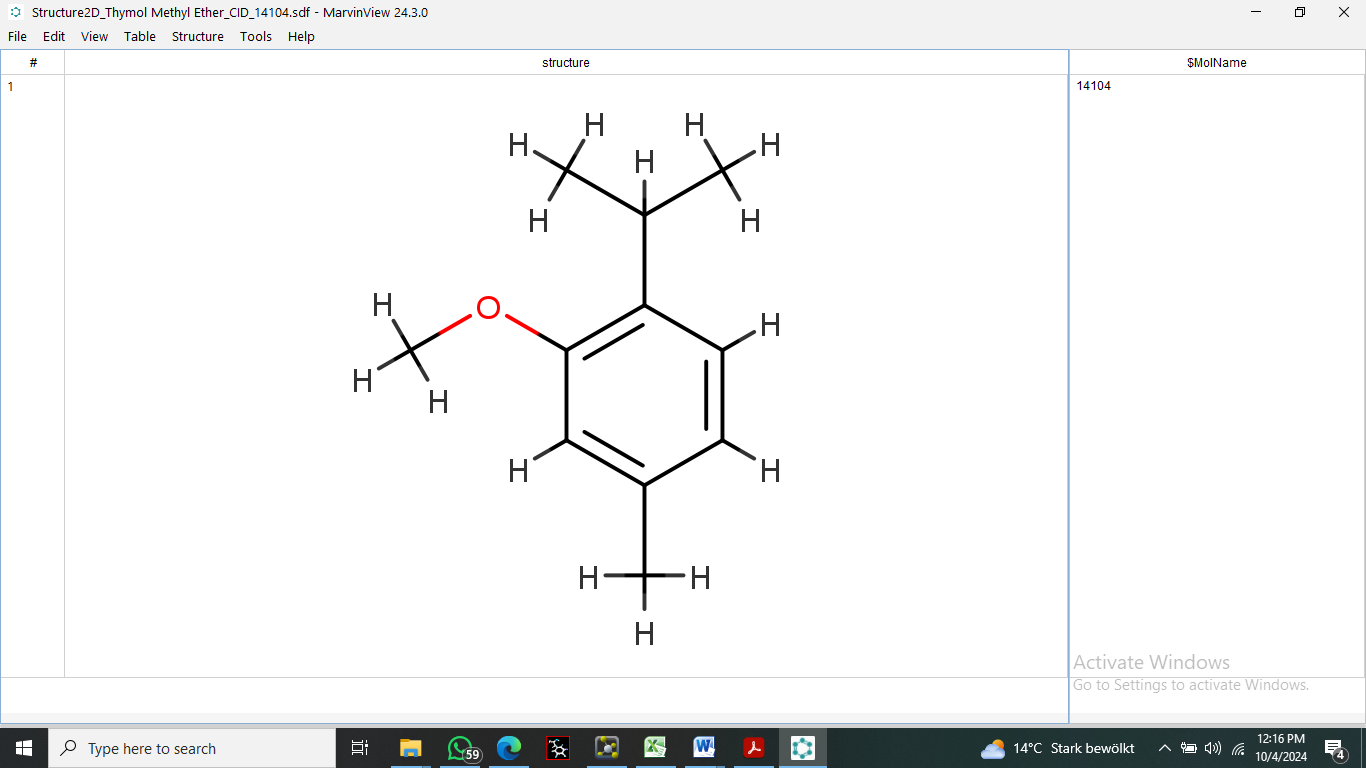

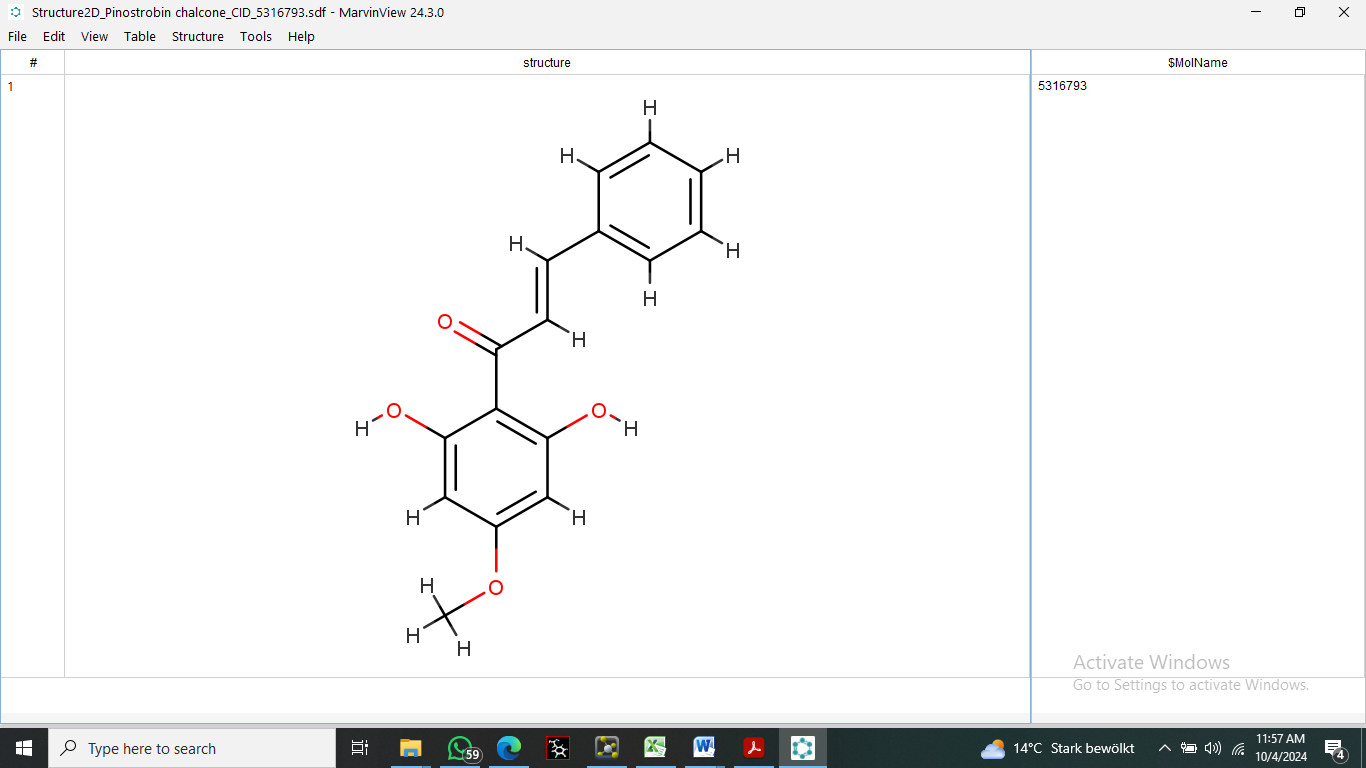


Thymol methyl ether

Glutaric acid,

di(2-methoxybenzyl) ester

cis-α-Necrodyl acetate

Guaiol

Pinostrobin chalcone


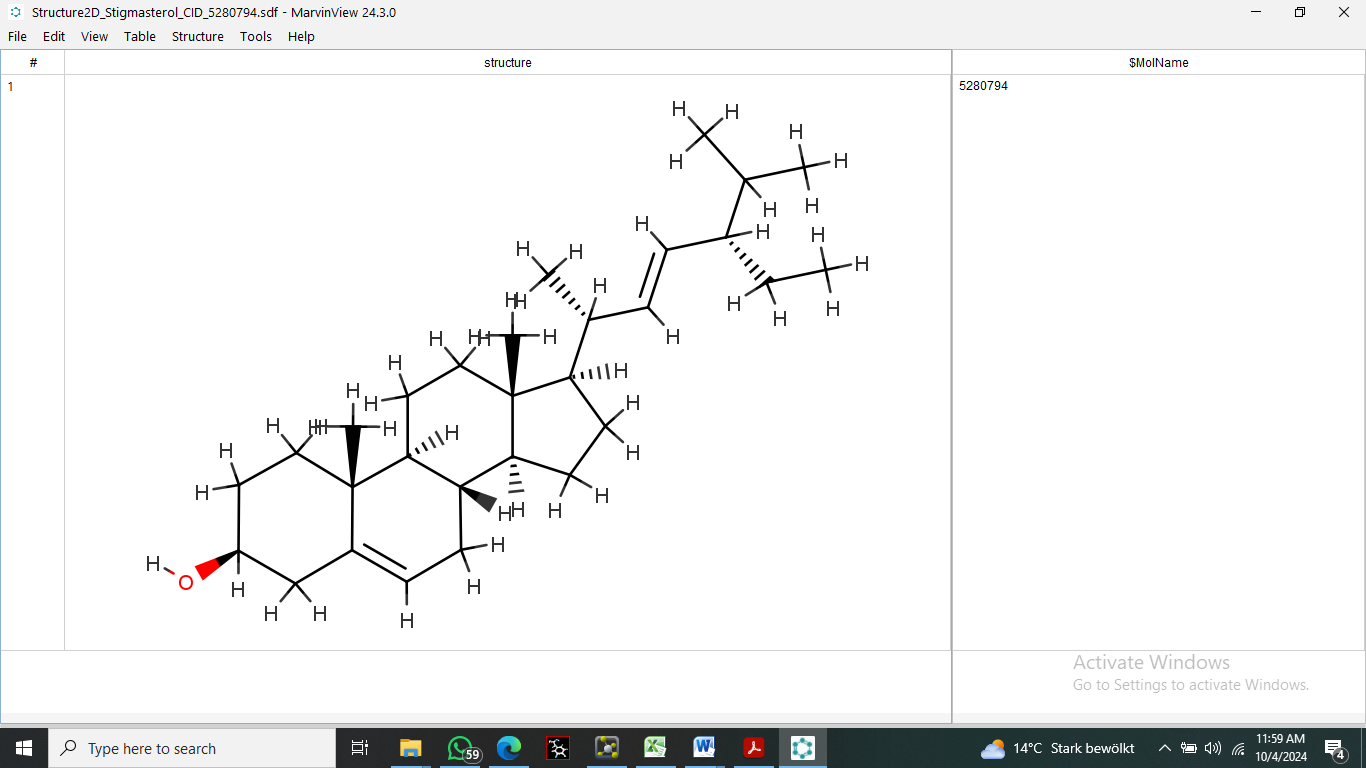

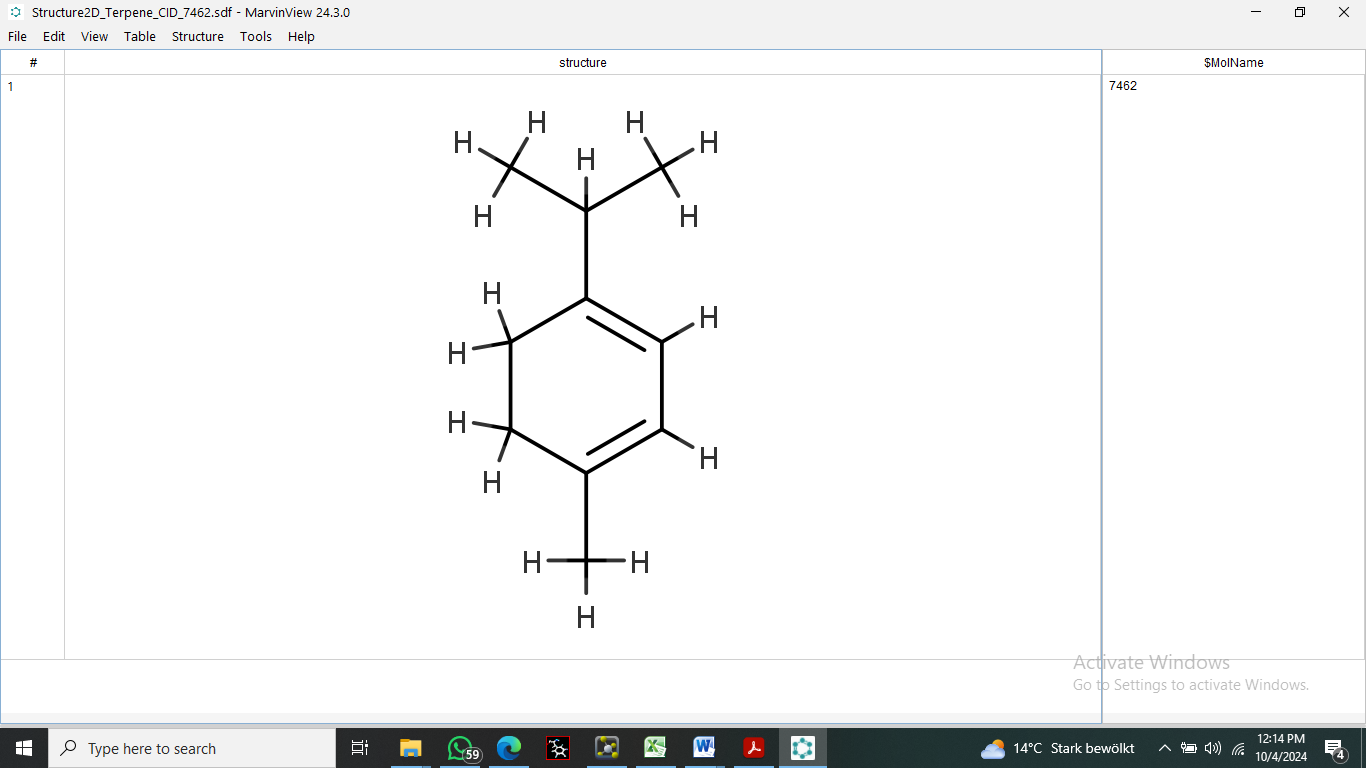

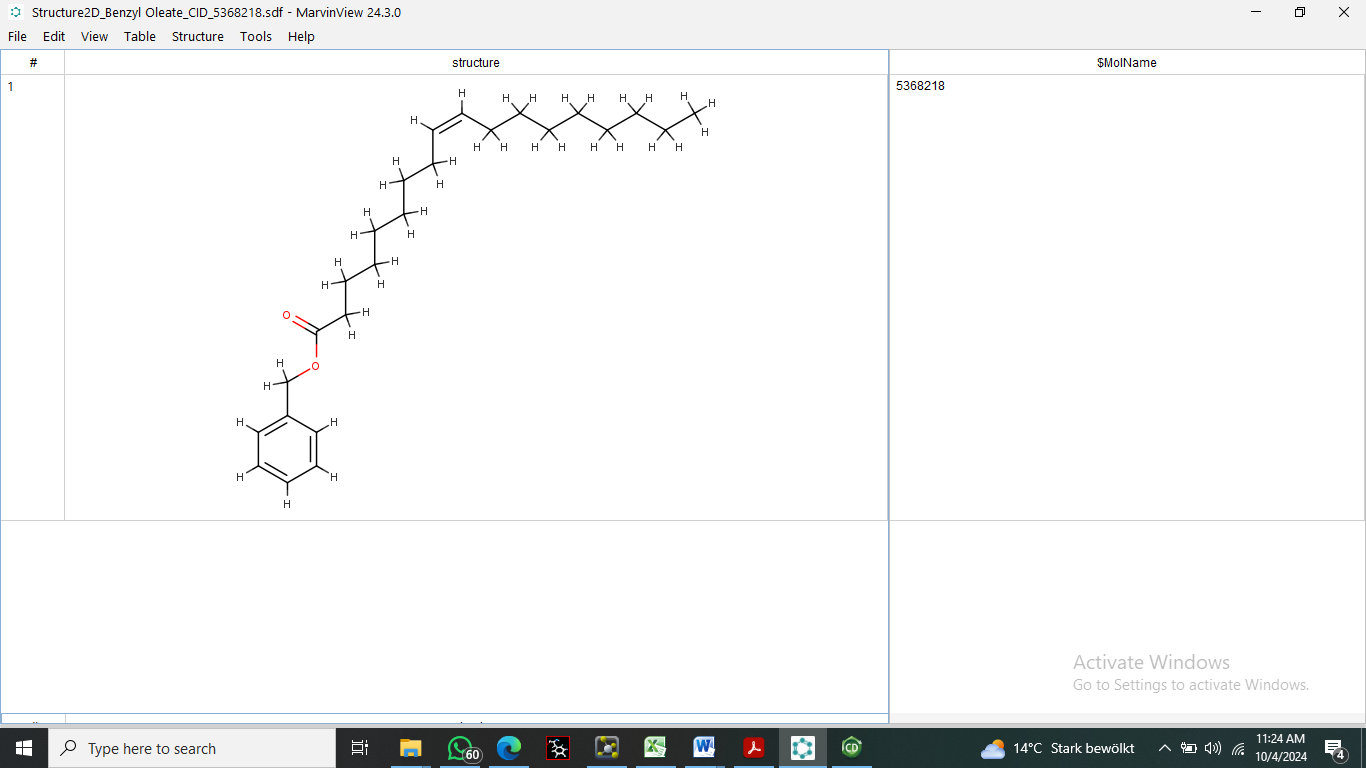

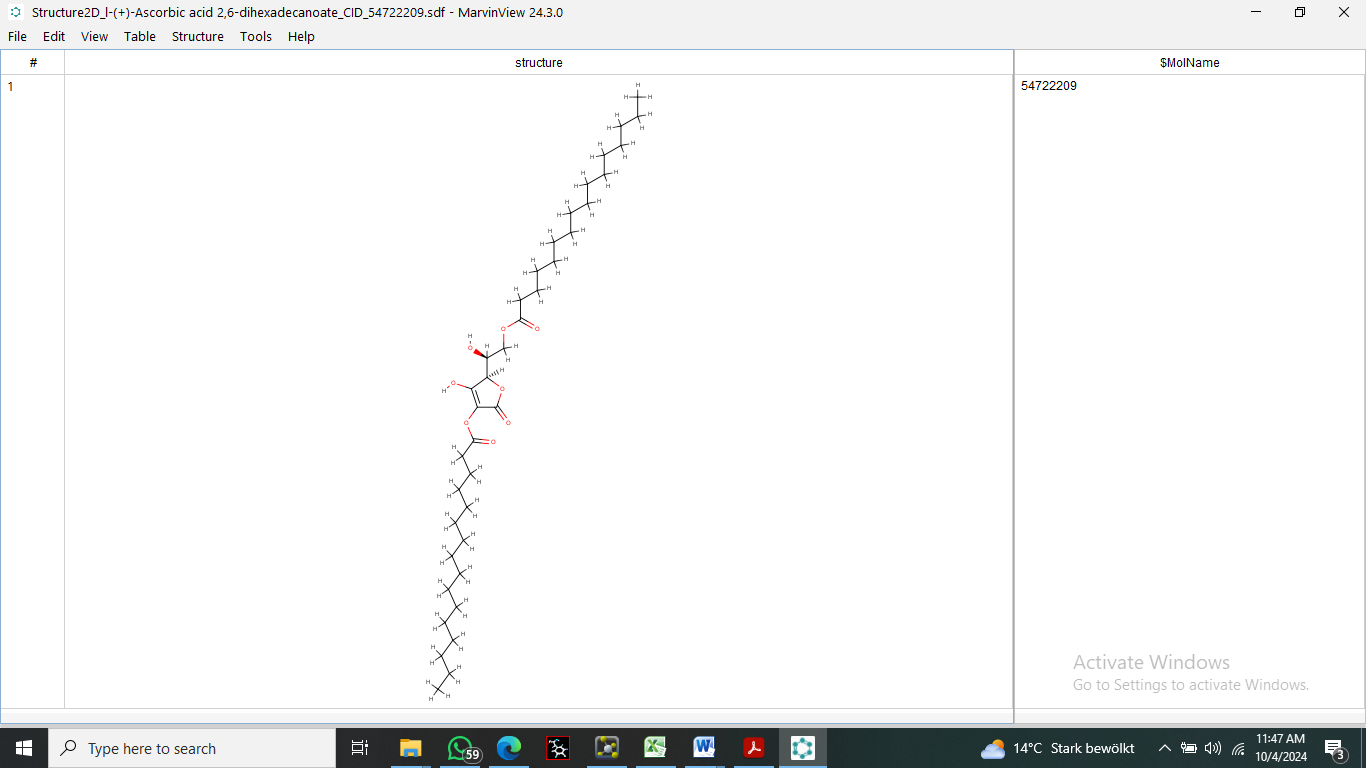

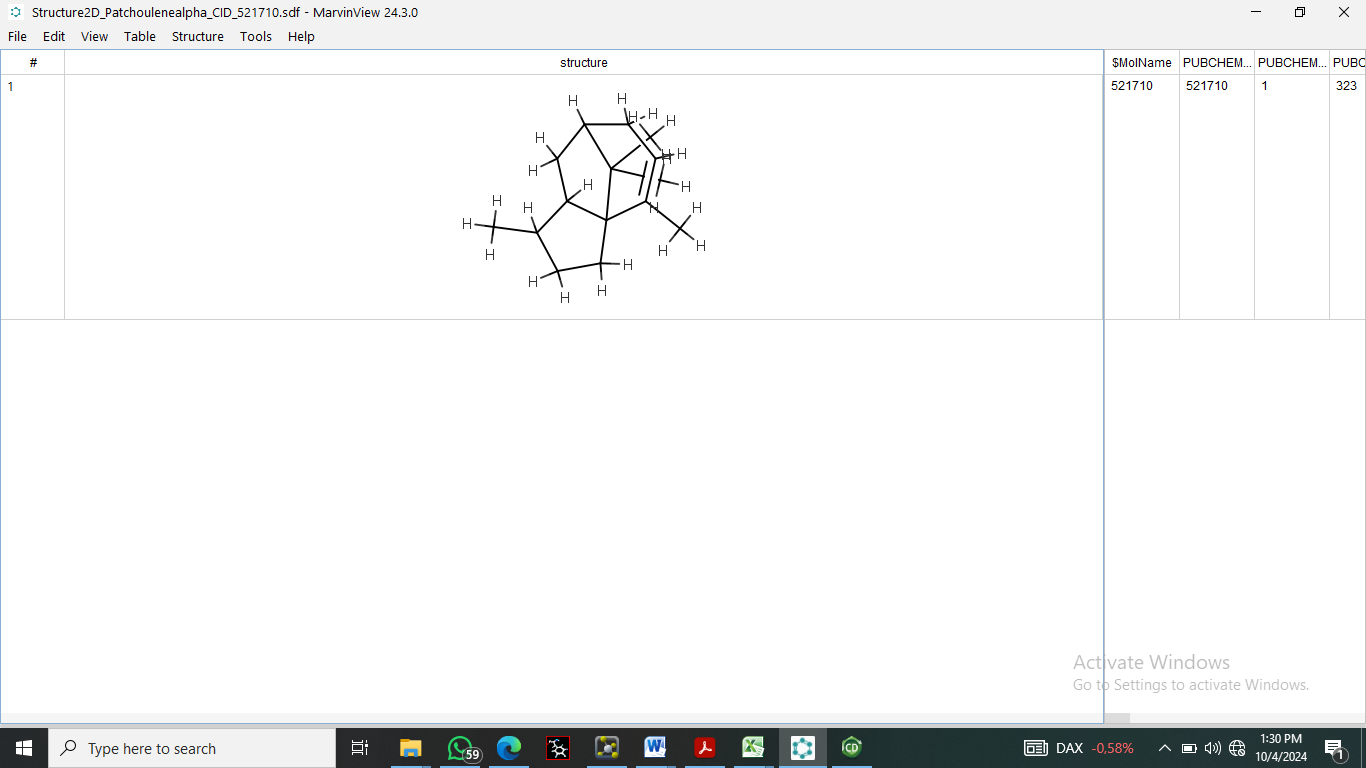


α-Patchoulene

l-(+)-Ascorbic acid 2,6-dihexadecanoate

Stigmasterol

α-terpinene

Benzyl oleate

Supplementary Figure 1. Chemical structures of bioactive compounds from essential oil of A. smeathmannii (DC.) Guill. & Perr. ) root.


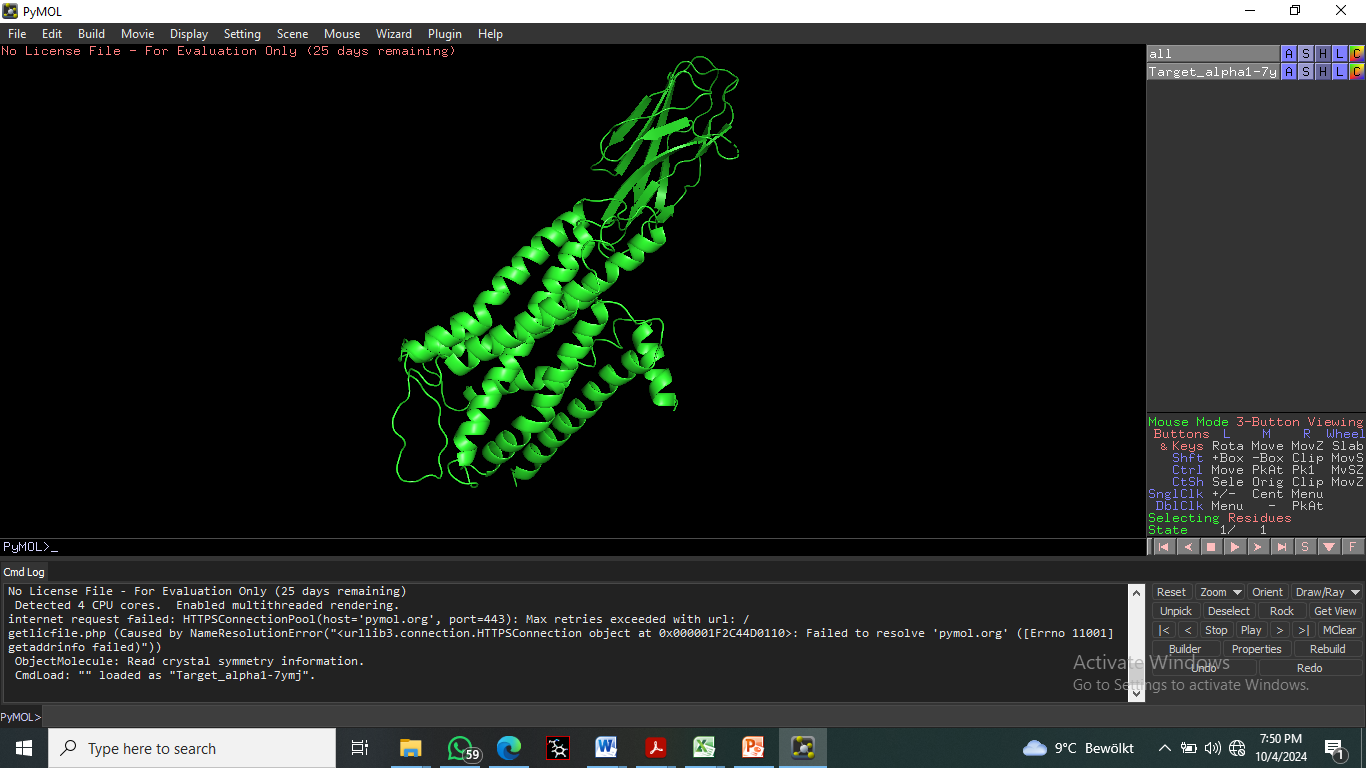

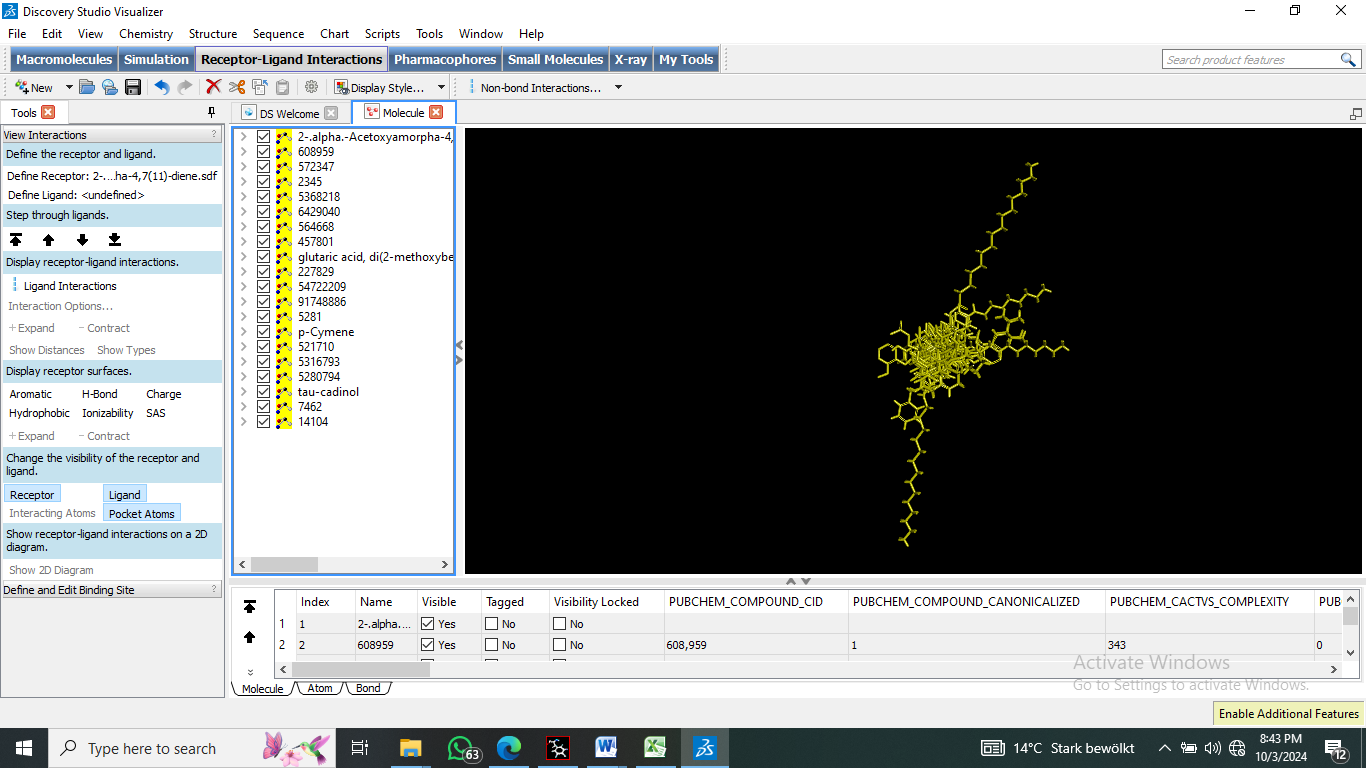


(A)

(B)

Supplementary Figure 2. Purified proteins of (A) Cryo-EM structure of alpha1AAR-Nb6 (7YMJ) (PDB DOI: <https://doi.org/10.2210/pdb7YMJ/pdb>). (B) Ligand_cluster.

. (a)
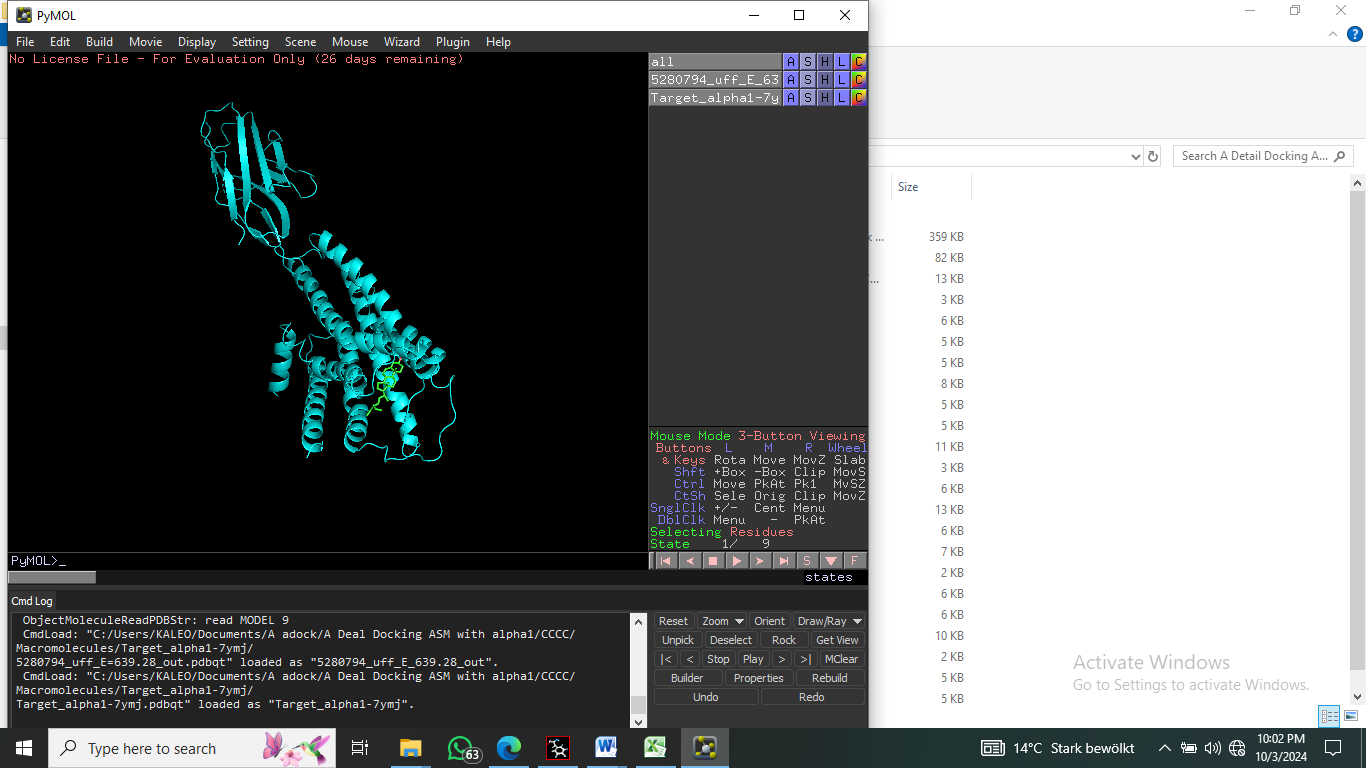

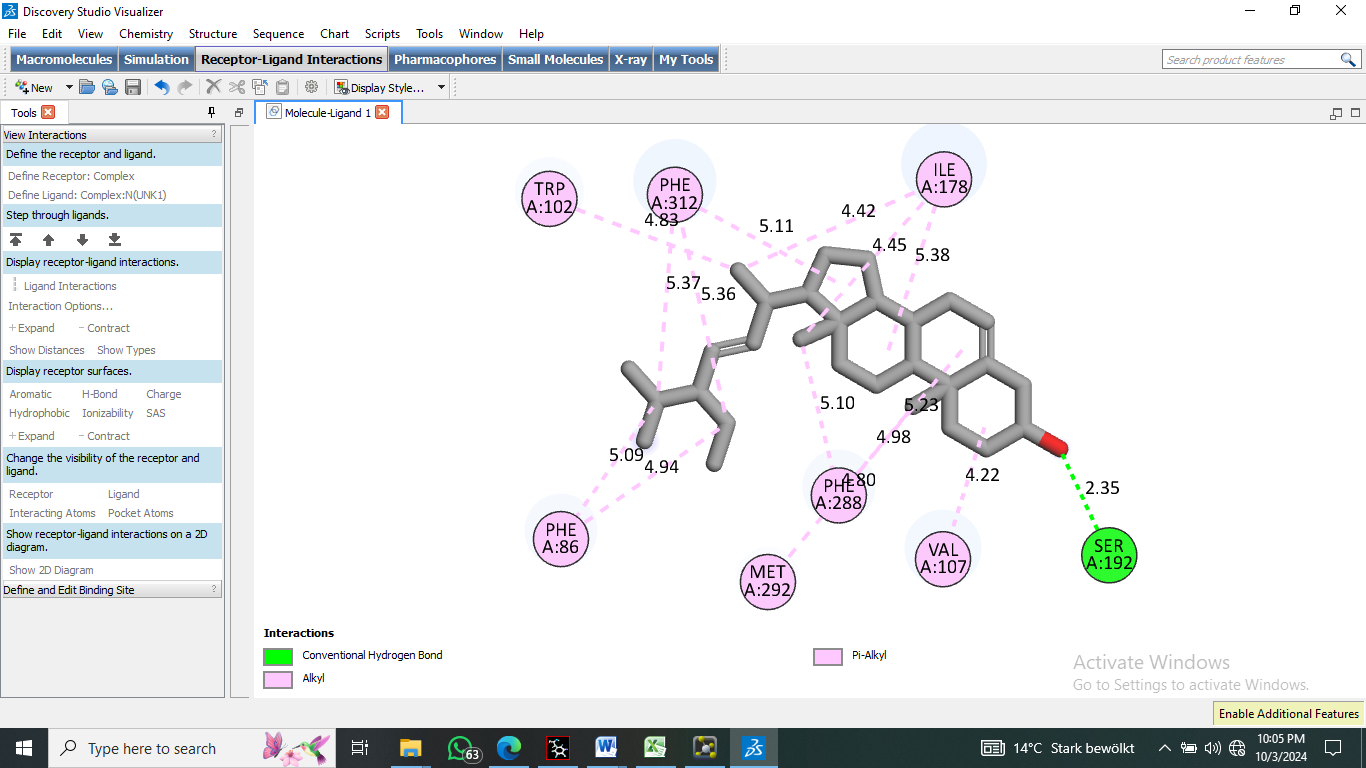


Stigmasterol (5280794)

(b)
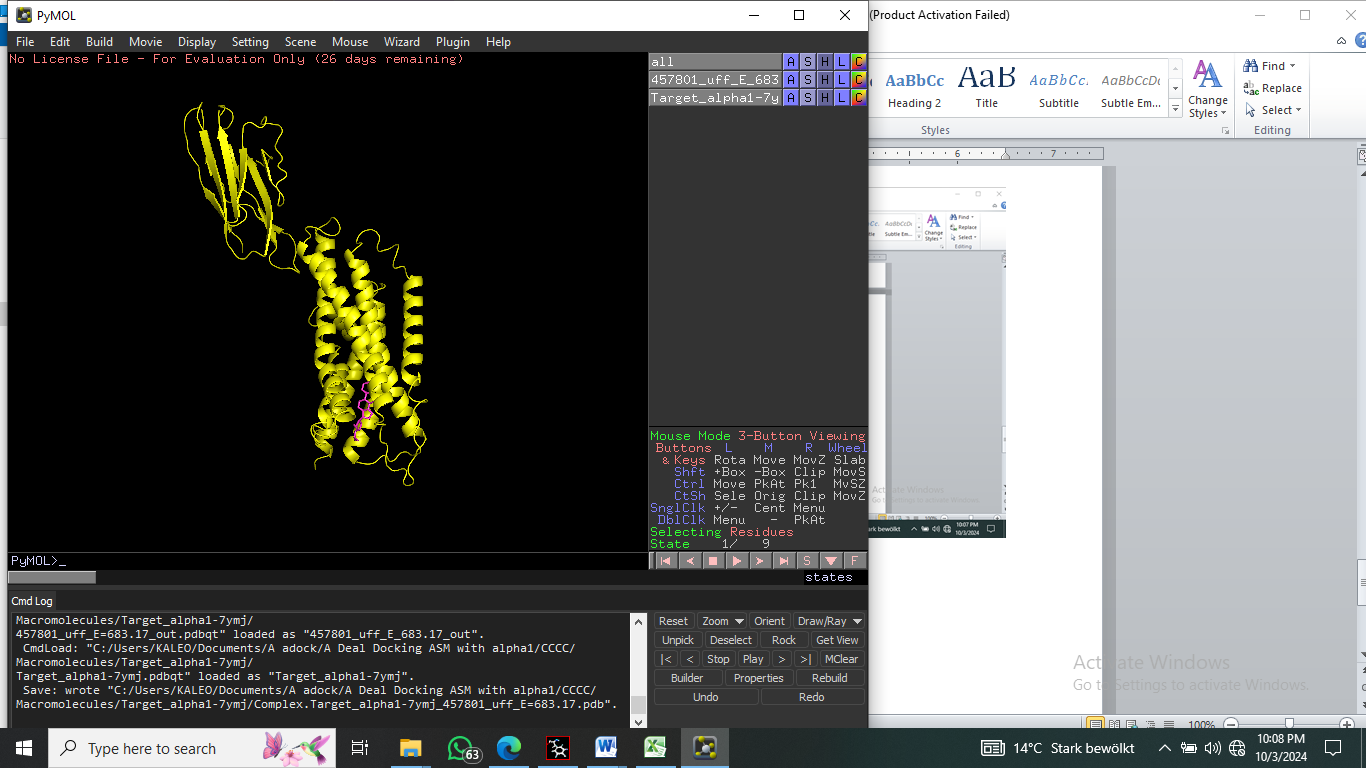

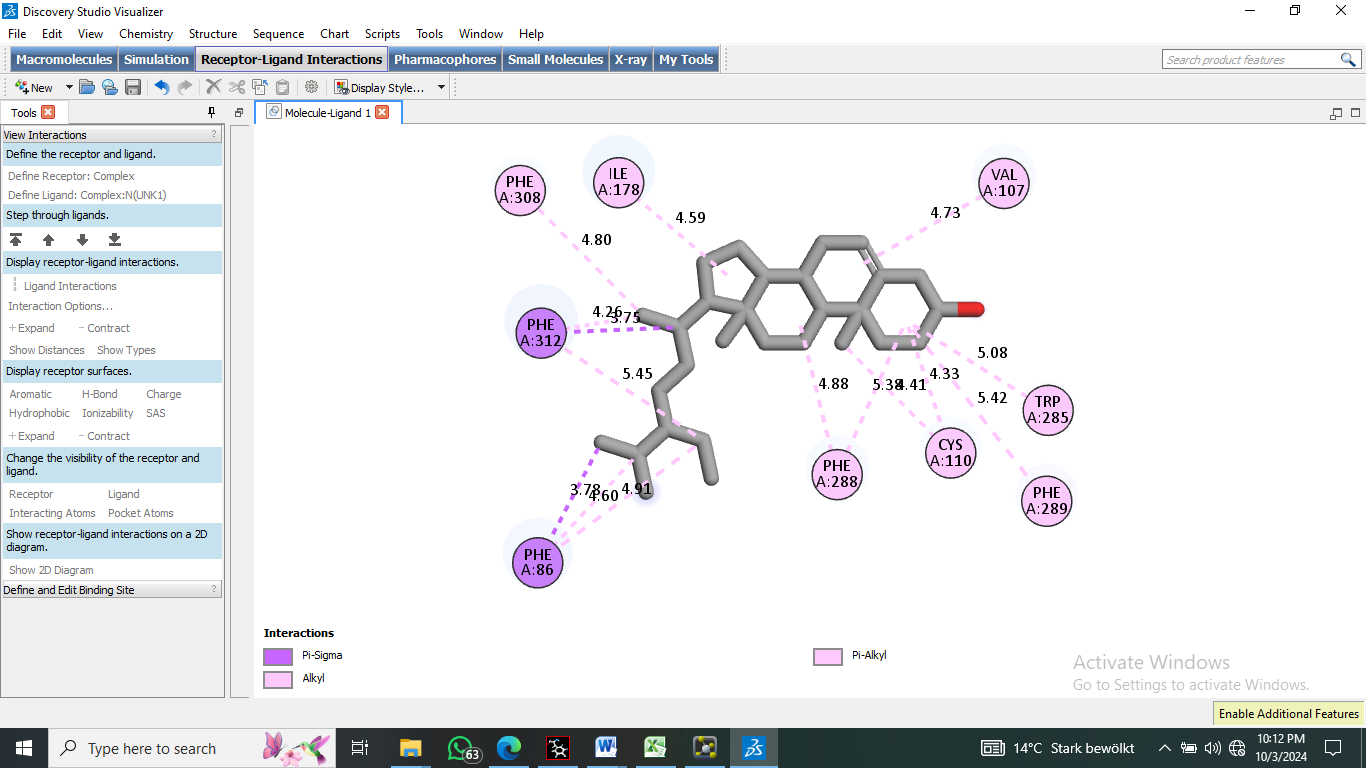


γ-Sitosterol (457801)

(c)
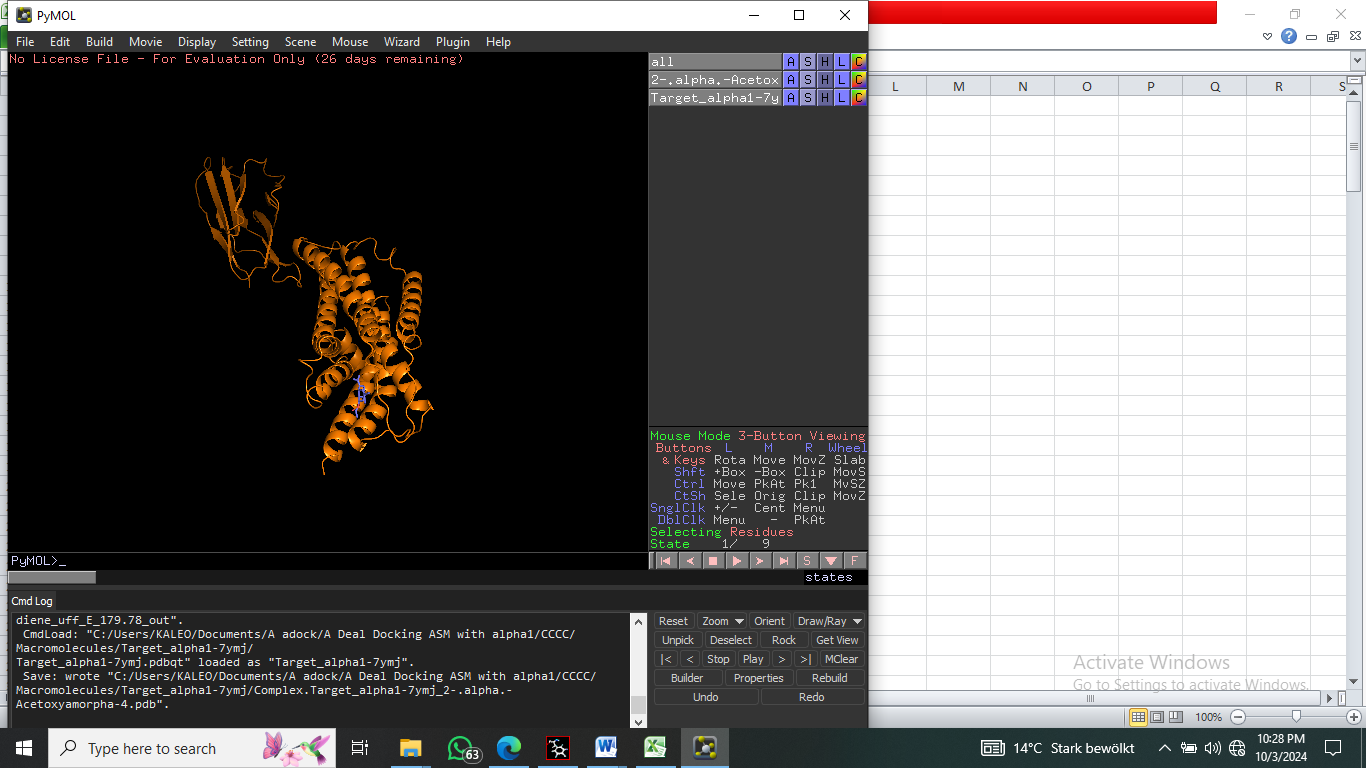

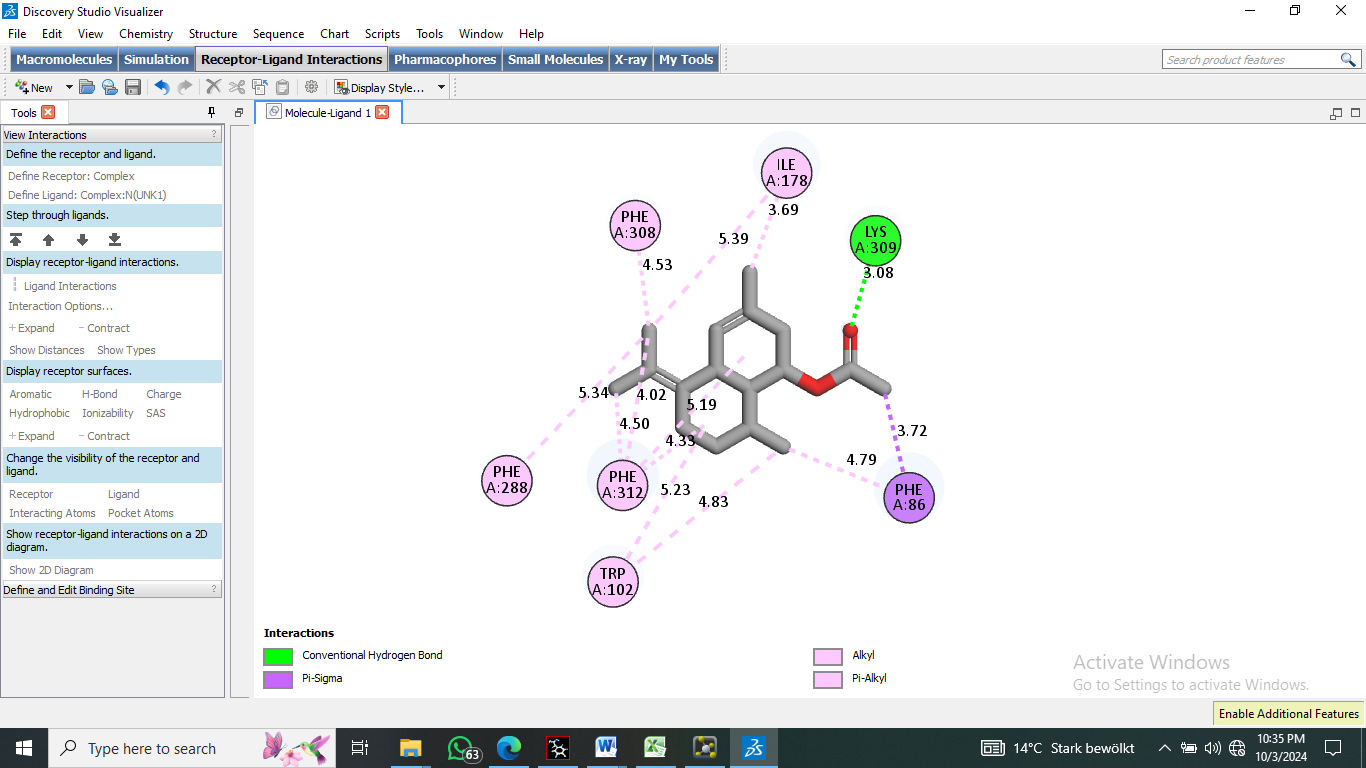


2-α-acetoxyamorpha-4,7(11)-diene (91752529)

(d)
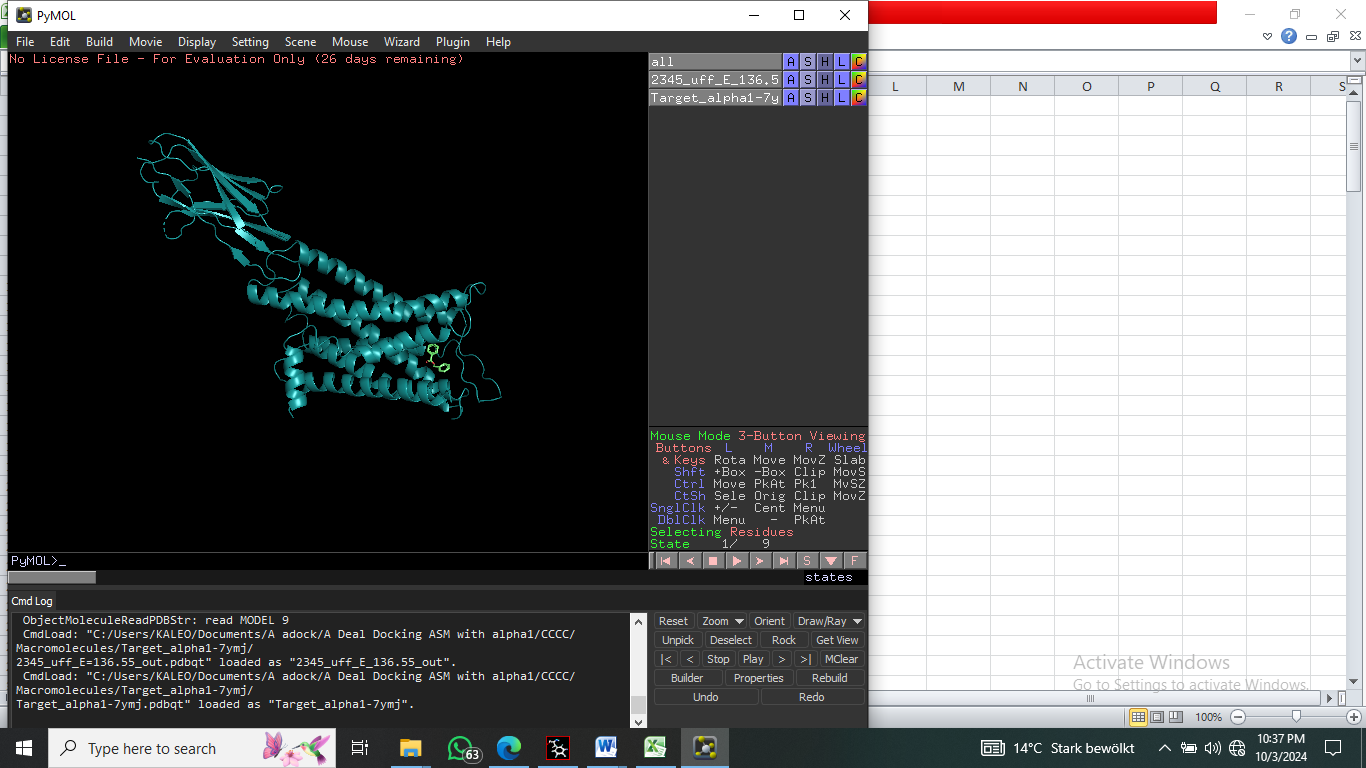

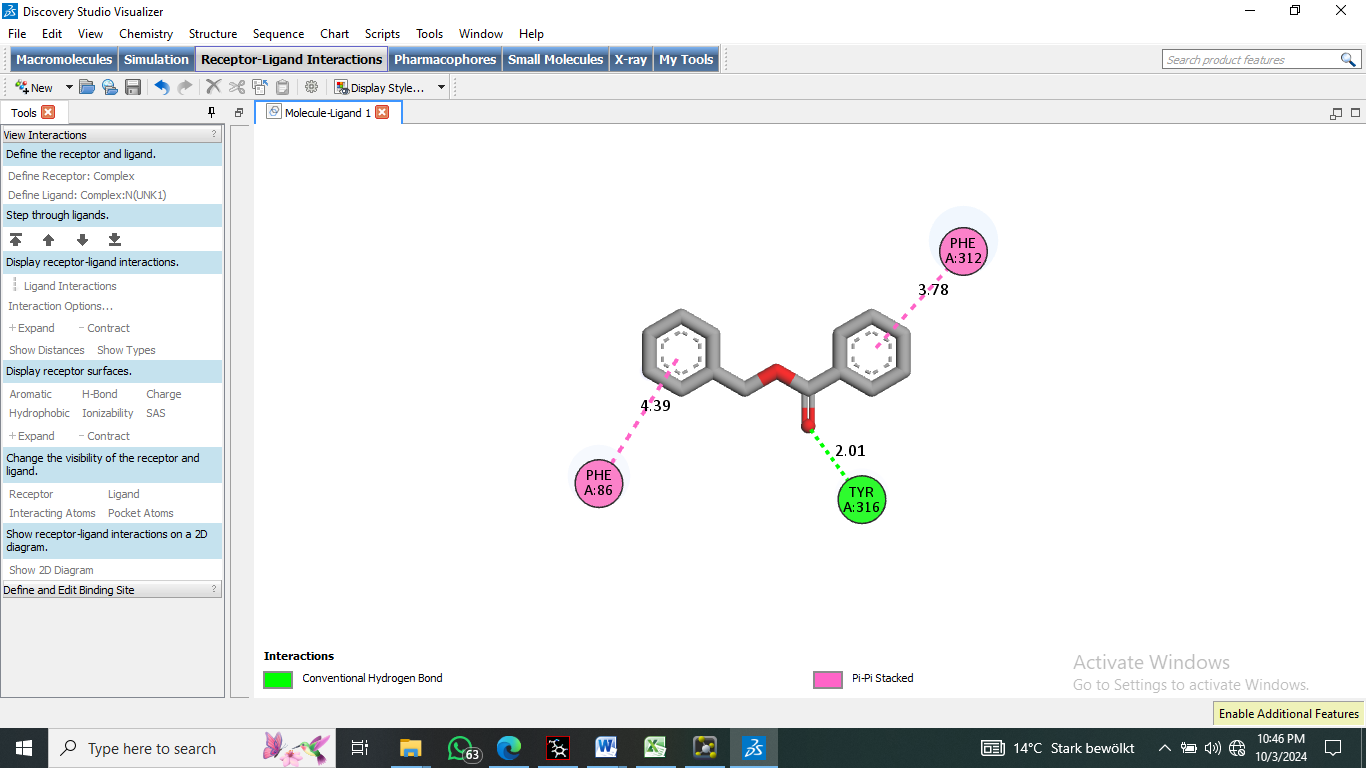


Benzyl Benzoate (2345)

(e)
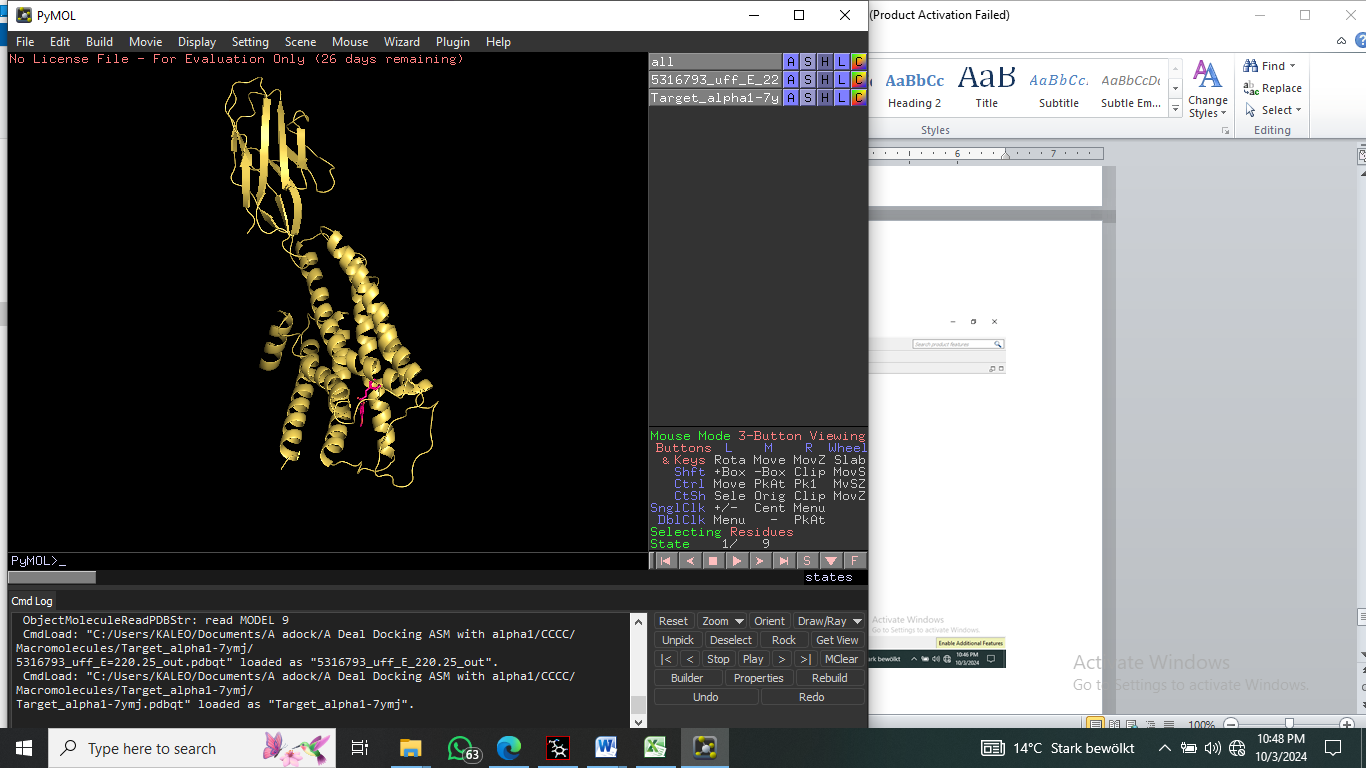

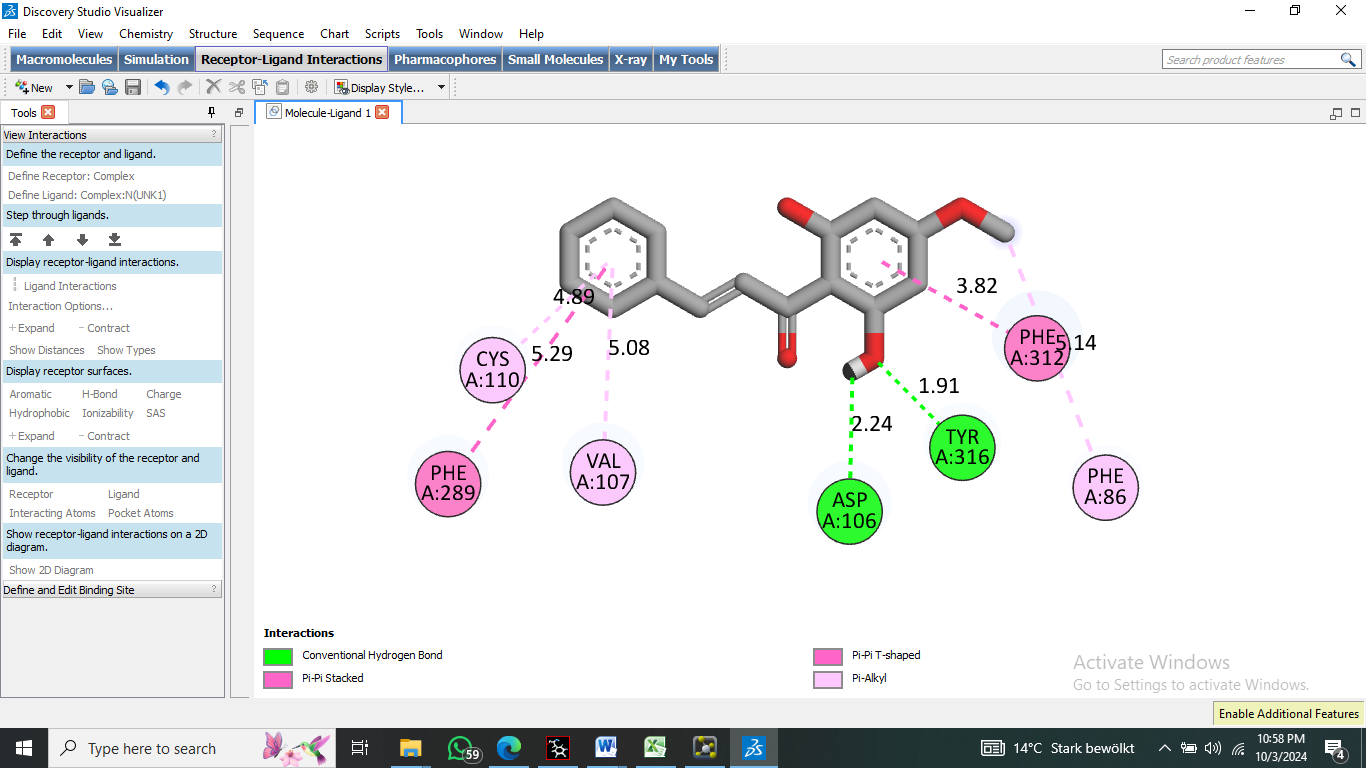


Pinostrobin chalcone (5316793)

(f)
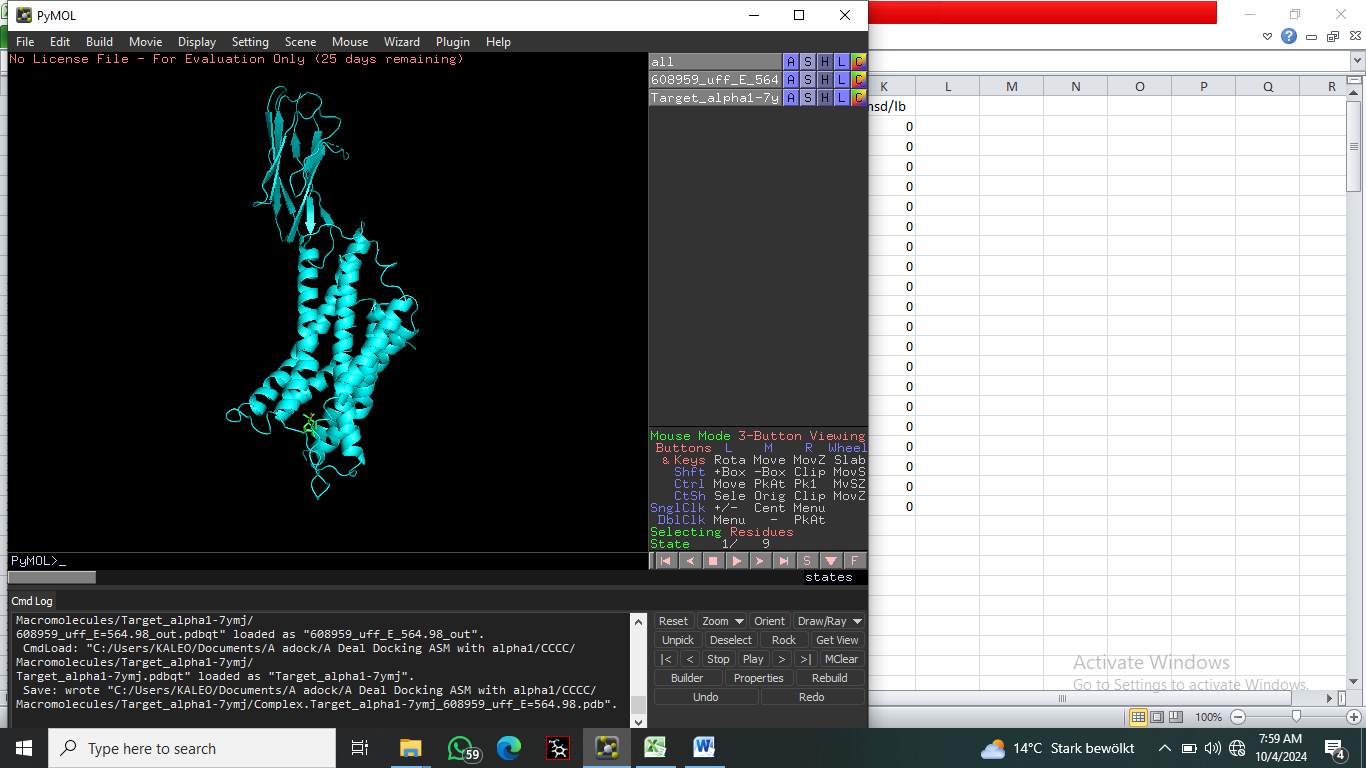

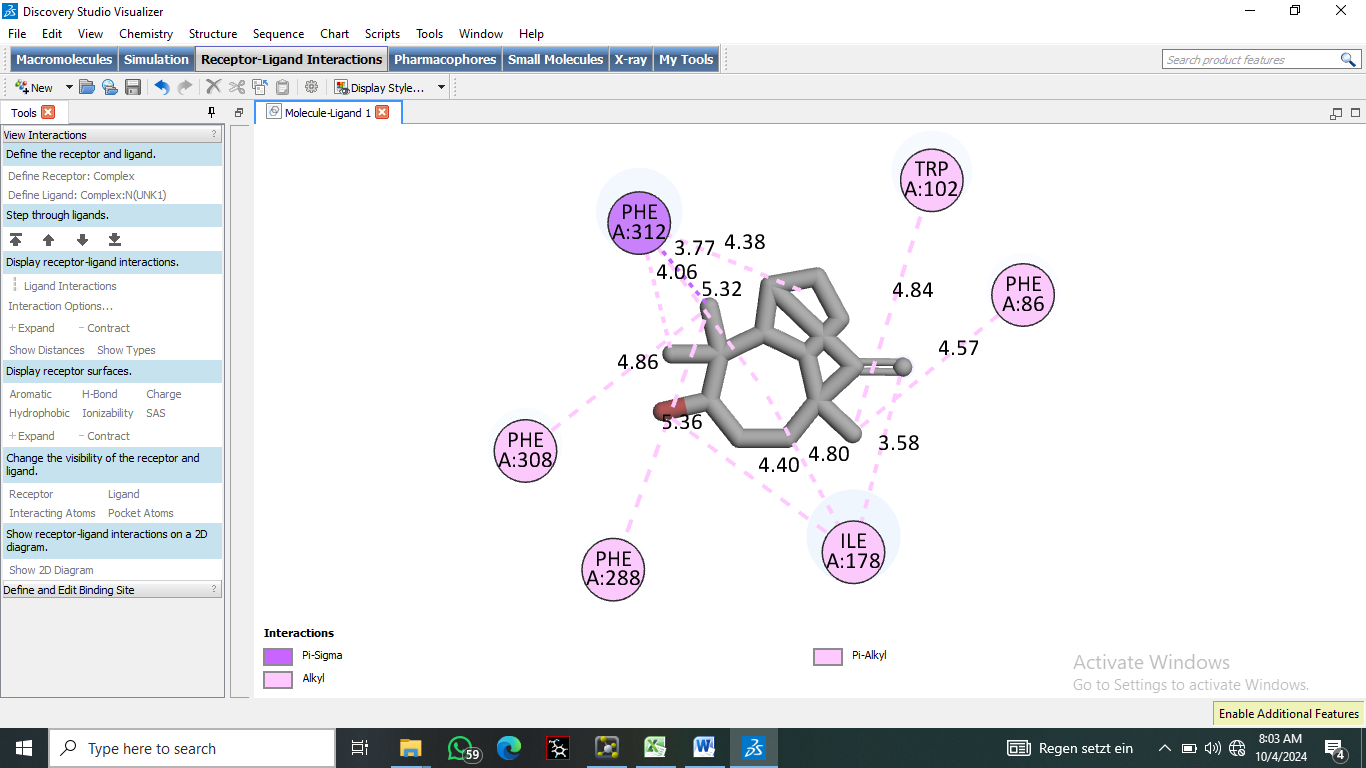


1,4-Methanoazulene (608959)

(g)
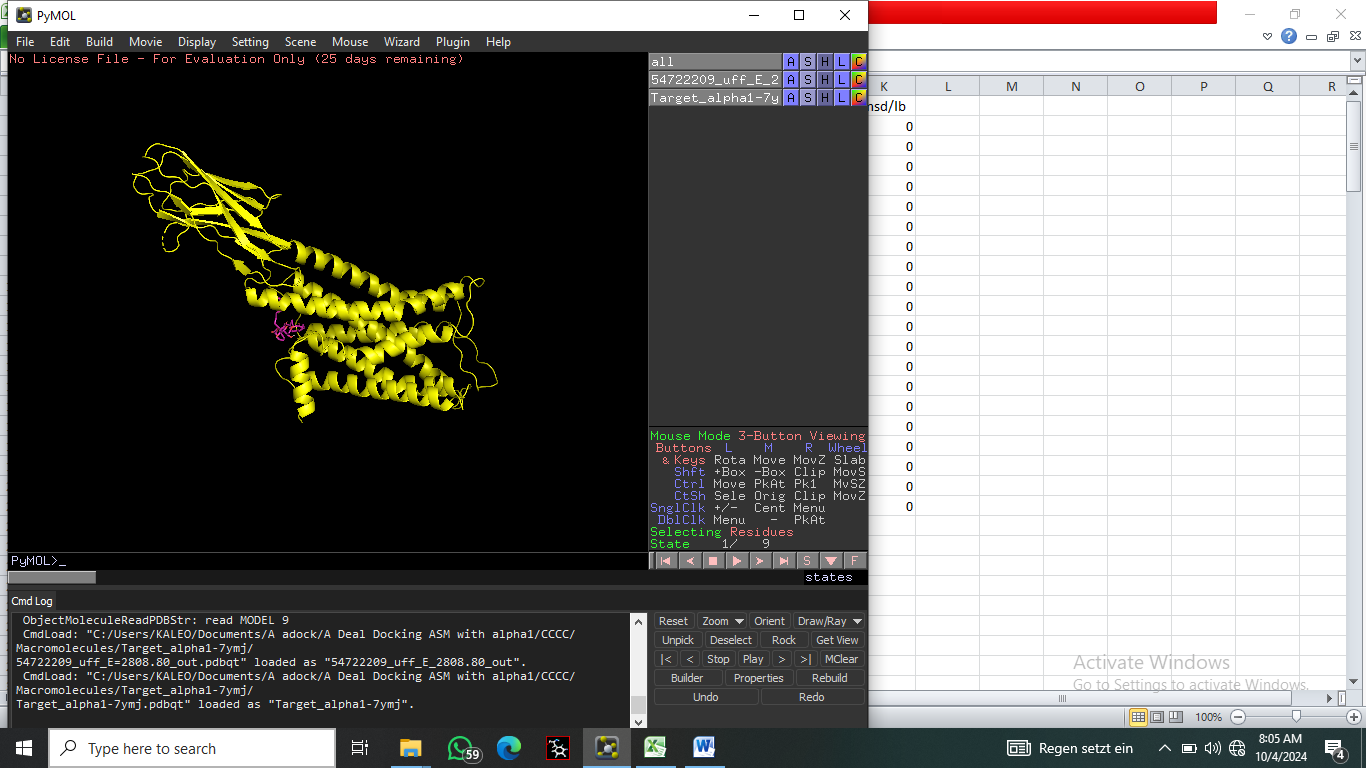

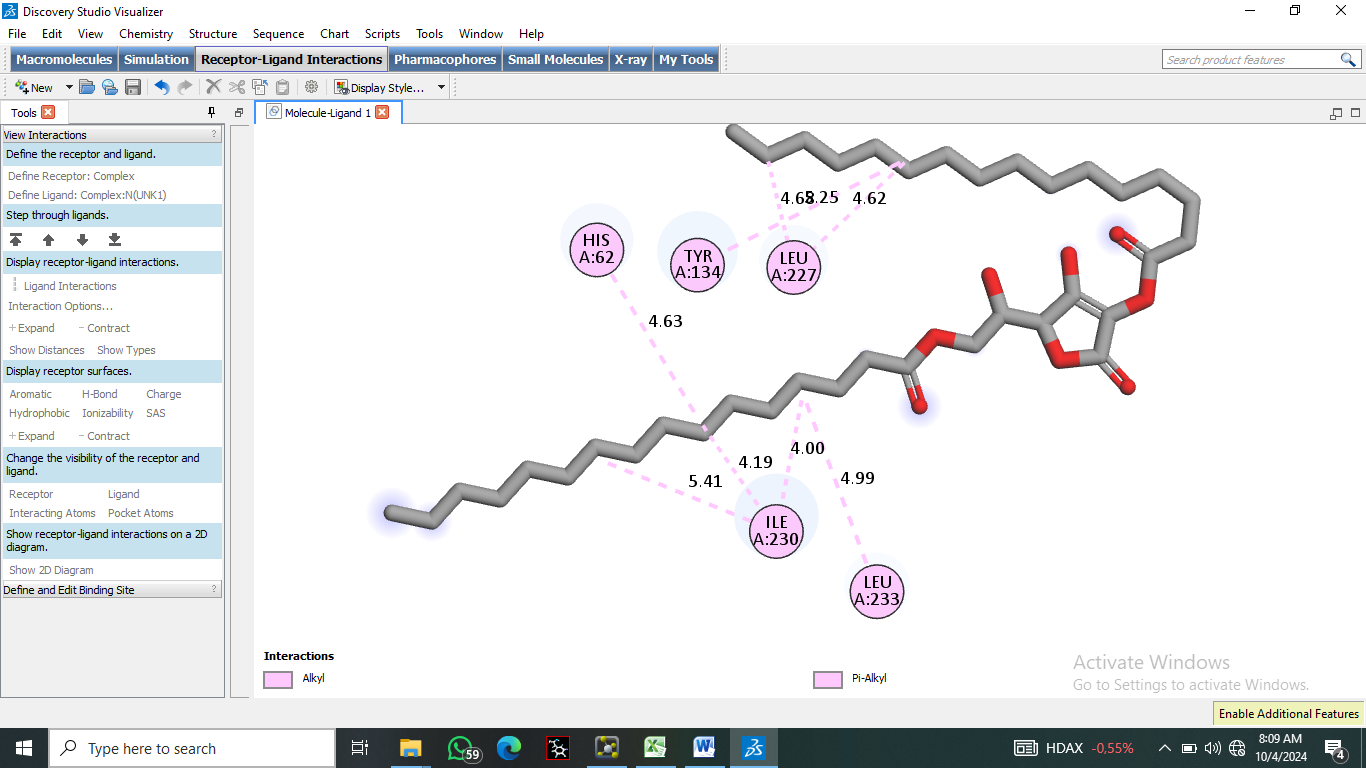


l-(+)-Ascorbic acid 2,6-dihexadecanoate (54722209)

(h)
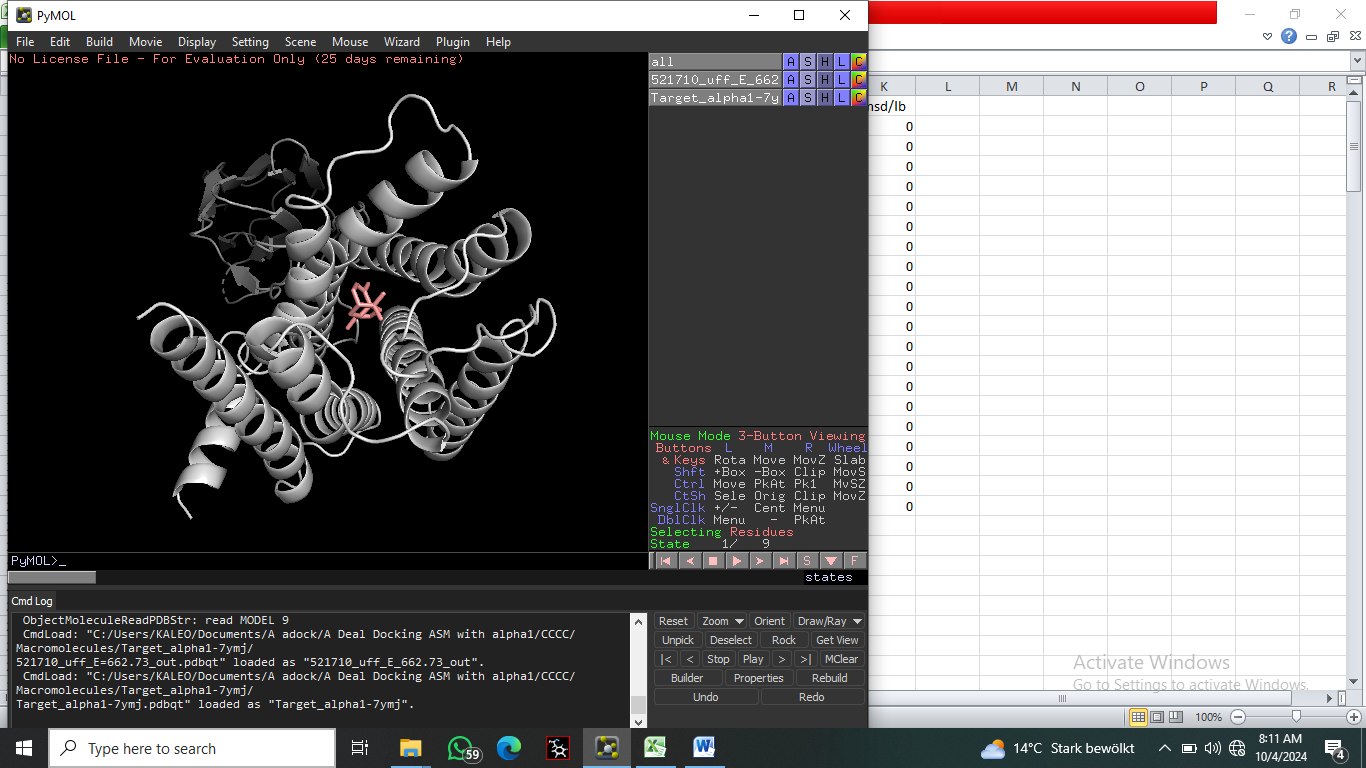

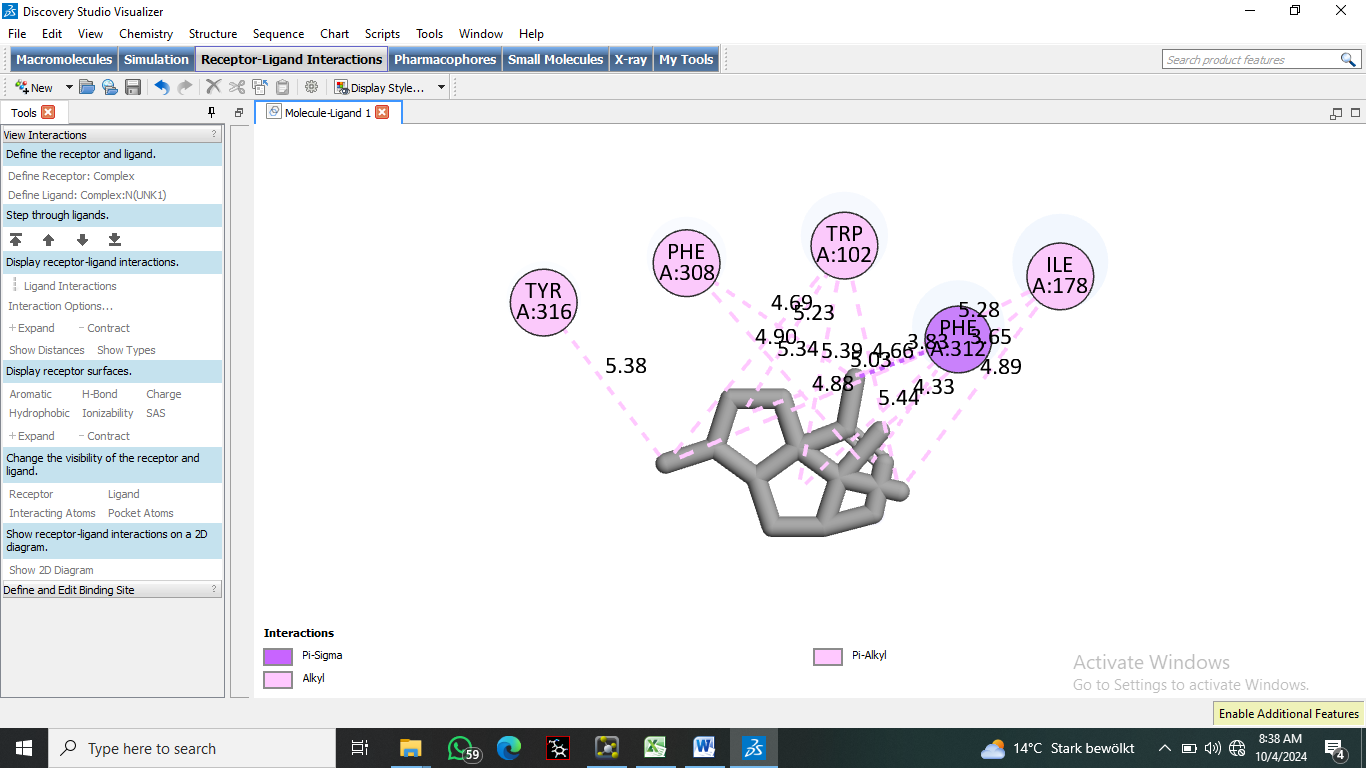


α-Patchoulene (521710)

(i)
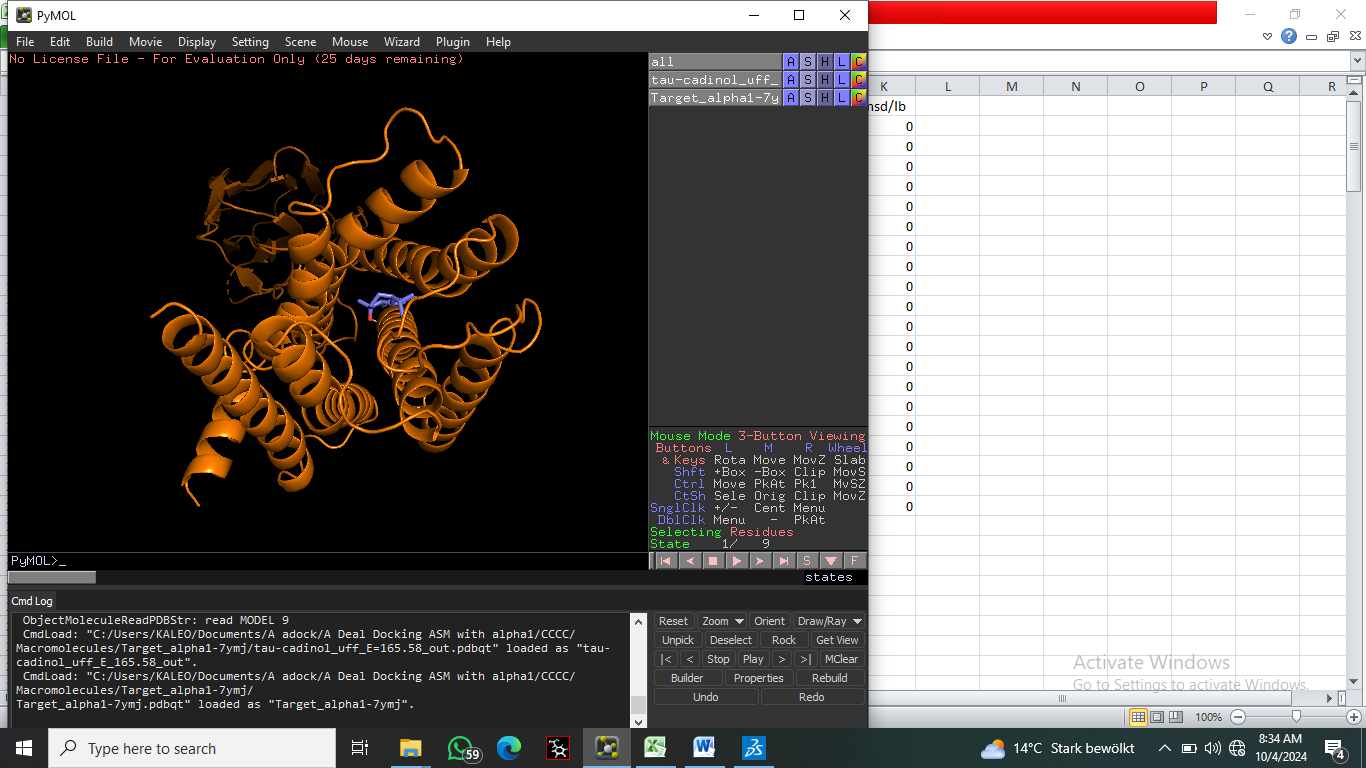

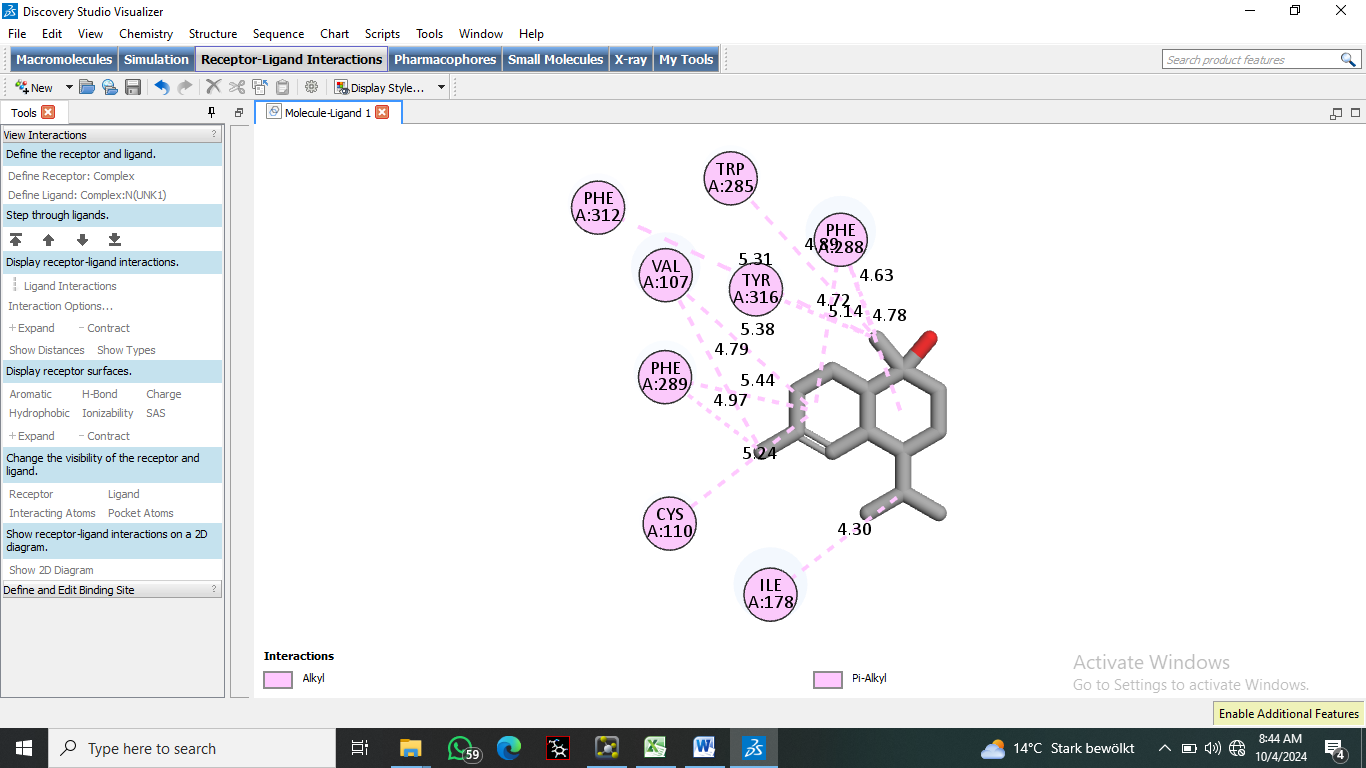


Tau-cadinol (160799)

(j)
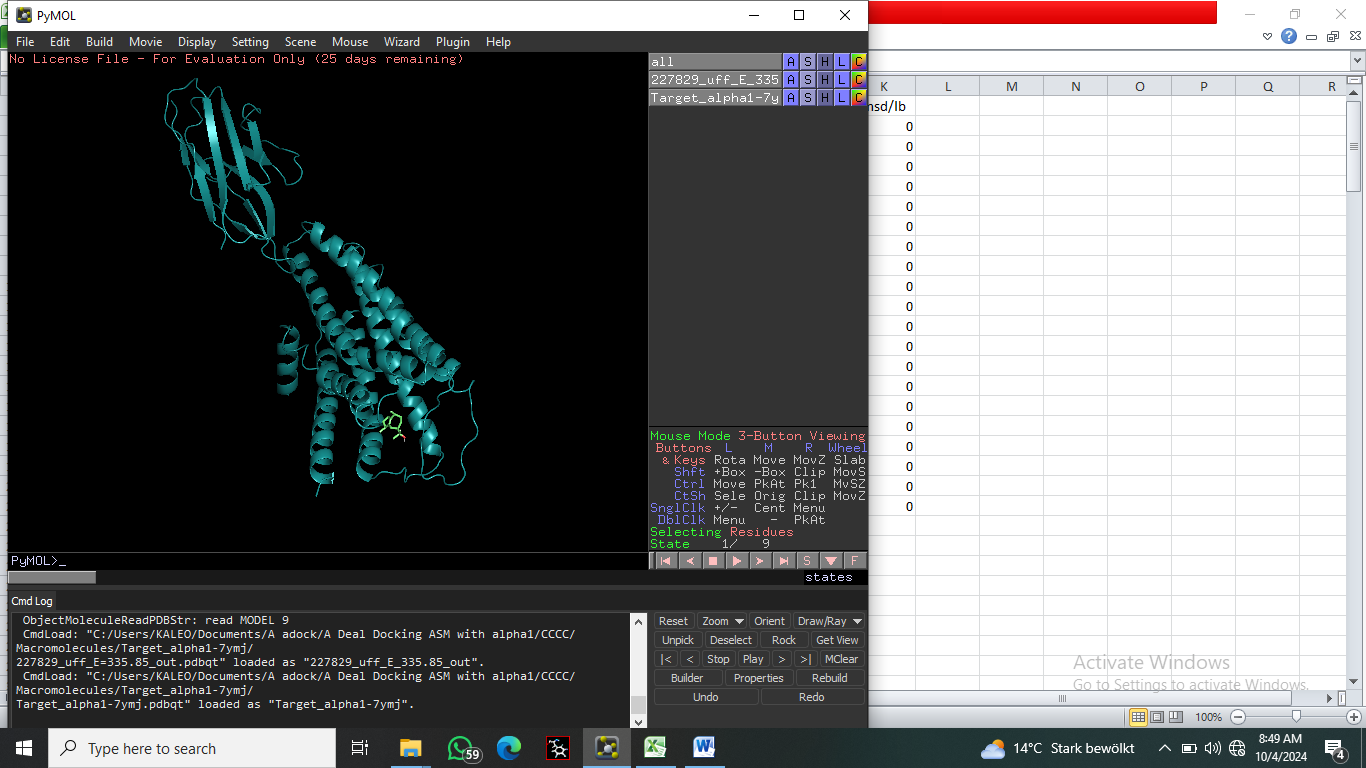

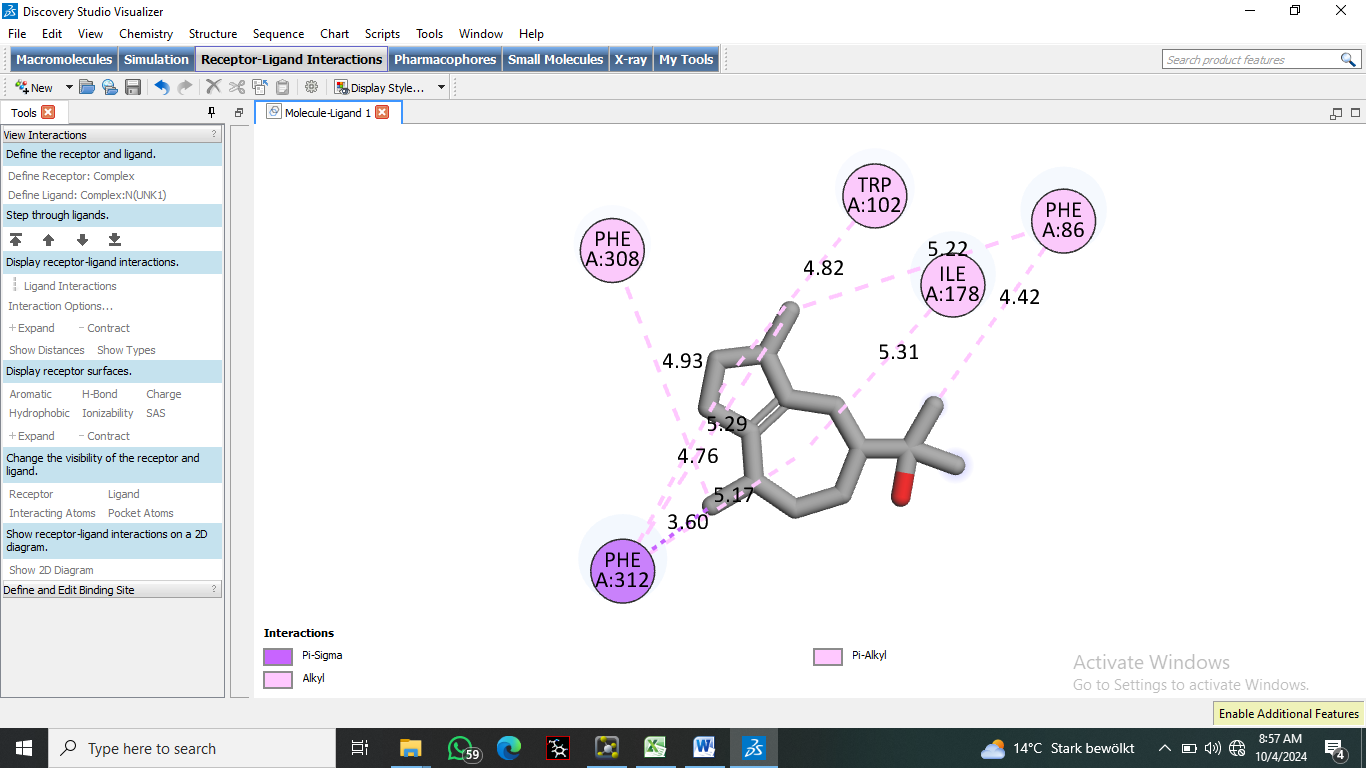


Guaiol (227829)


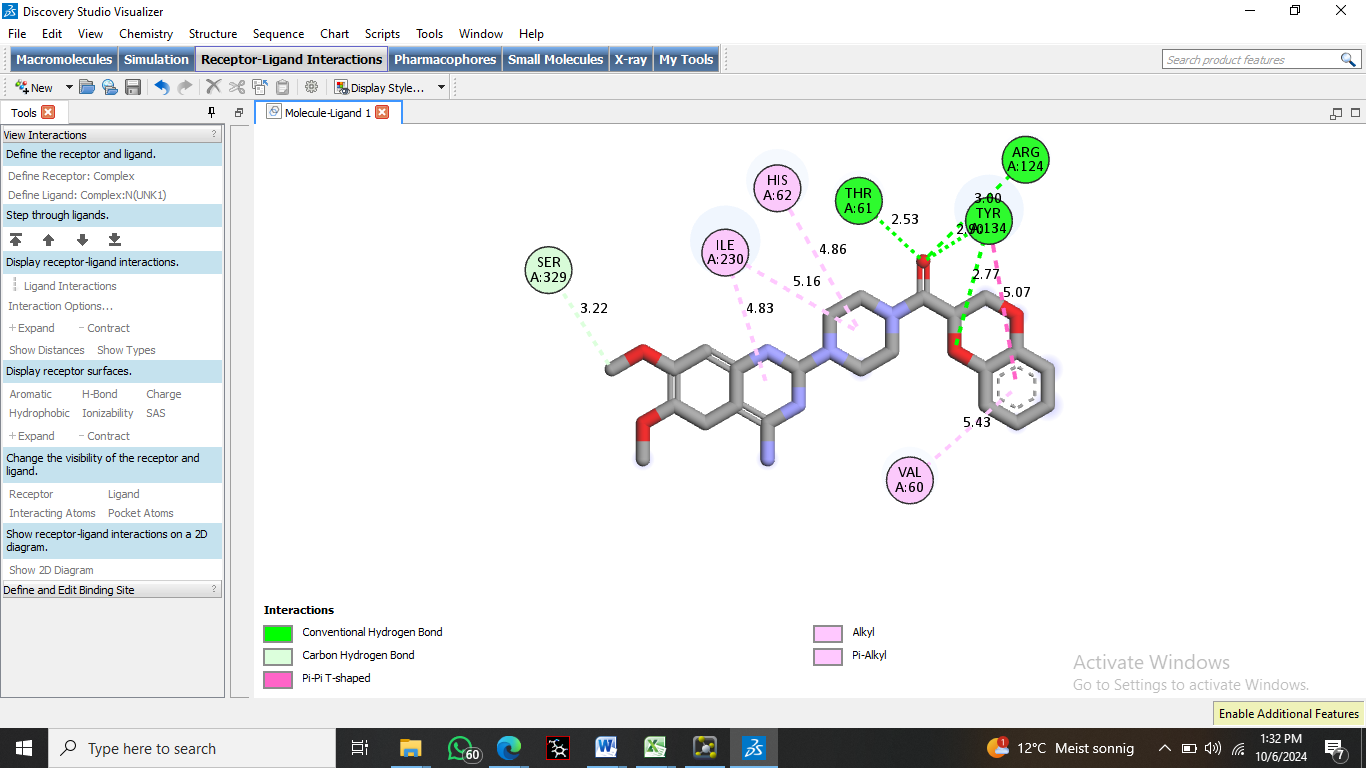

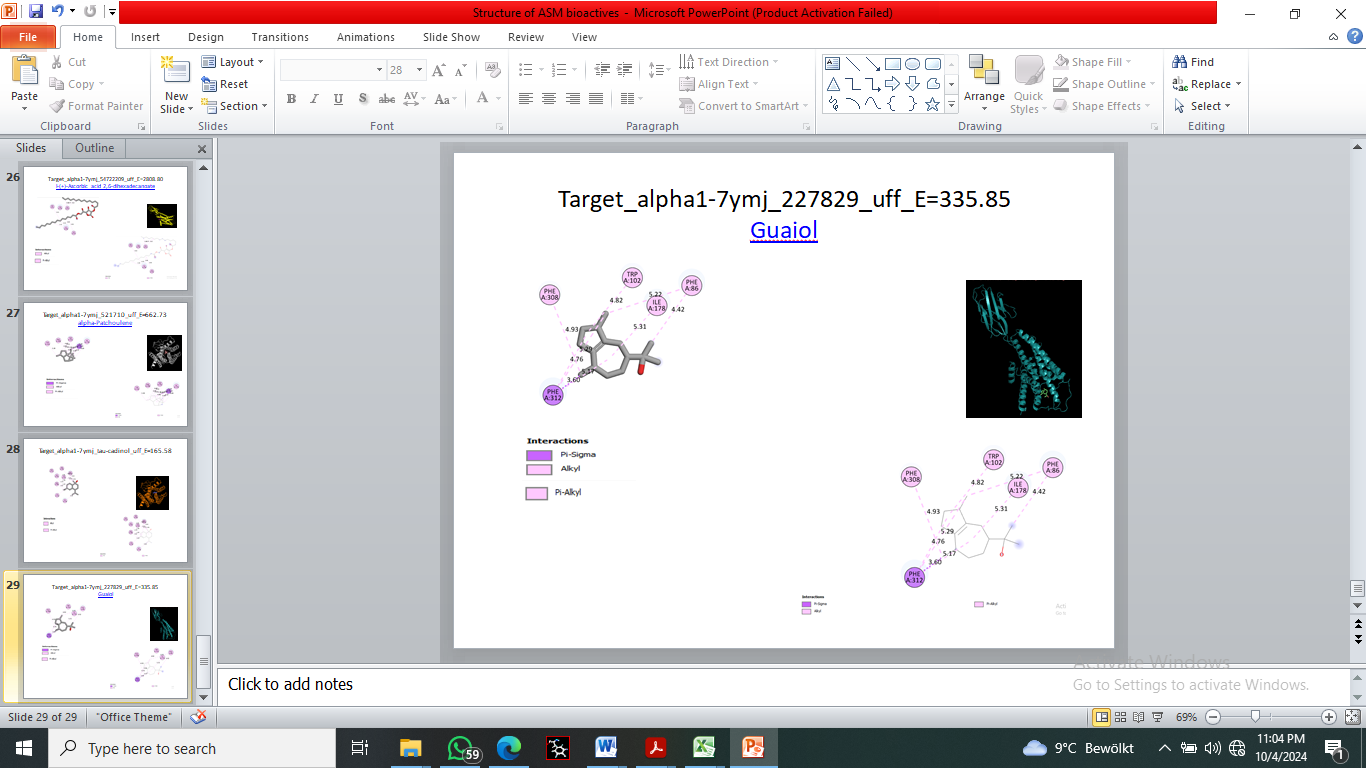


Supplementary Figure 3. Showing 3D and 2D diagram interaction of processed alpha1AAR-Nb6 (7ymj) with (a - j) *A. smeathamannii* bioactive compounds.

Supplementary table 2. SwissADME-pkCSM Comparison of Pharmacokinetic, Physicochemical and Medicinal Chemistry Properties *A. smeathmannii* Bioactives.

| Physicochemical Properties | 1,4-Methanoazulene | 2-α-acetoxyamorpha-4,7(11)-diene | α.Patchoulene | Benzyl Benzoate | γ.-Sitosterol | Glutaric acid, di(2-methoxybenzyl) ester | l-(+)-Ascorbic acid 2,6-dihexadecanoate | Pinostrobin chalcone | Stigmasterol | Tau-cadinol | Guaiol | Tamsulosin |
| --- | --- | --- | --- | --- | --- | --- | --- | --- | --- | --- | --- | --- |
| Num. H-bond acceptors | 0 | 0 | 0 | 2 | 1 | 6 | 8 | 4 | 1 | 1 | 1 | 7 |
| Num. H-bond donors | 0 | 0 | 0 | 0 | 1 | 0 | 2 | 2 | 1 | 1 | 1 | 2 |
|  |  |  |  |  |  |  |  |  |  |  |  |  |
| Lipophilicity |  |  |  |  |  |  |  |  |  |  |  |  |
| L og Po/w | 3.26 | 2.92 | 3.19 | 2.68 | 5.07 | 3.98 | 8.16 | 1.88 | 5.08 | 3.21 | 3.08 | 3.09 |
|  |  |  |  |  |  |  |  |  |  |  |  |  |
| Water Solubility | 1.82e-05 mol/l (M) | 1.33e-04 mol/l (M) | 7.73e-05 mol/l (M) | 1.13e-04 mol/l (M) | 1.26e-08 mol/l (L) | 2.11e-04 mol/l (L) | 3.15e-11 mol/l (I) | 1.26e-04 mol/l (M) | 3.46e-08 mol/l (L) | 5.54e-04 mol/l (M) | 8.19e-04 mol/l (M) | 2.07e-04 mol/l (M) |
|  |  |  |  |  |  |  |  |  |  |  |  |  |
| Pharmacokinetics |  |  |  |  |  |  |  |  |  |  |  |  |
| GI absorption | Low | Low | Low | High | Low | High | Low | High | Low | High | High | High |
| P-gp substrate | No | No | No | No | No | No | Yes | No | No | No | No | No |
| P-glycoprotein I inhibitor | No | Yes | No | No | Yes | Yes | Yes | No | Yes | No | No | Yes |
| P-glycoprotein II inhibitor | No | No | No | No | Yes | Yes | Yes | No | Yes | No | No | Yes |
| CYP1A2 inhibitor | No | No | No | Yes | No | Yes | No | Yes | No | No | No | Yes |
| CYP2C19 inhibitor | Yes | No | Yes | Yes | No | Yes | No | No | No | Yes | No | No |
| CYP2C9 inhibitor | Yes | Yes | Yes | No | No | Yes | No | Yes | Yes | No | No | Yes |
| CYP2D6 inhibitor | No | No | No | No | No | Yes | No | No | No | No | No | Yes |
| CYP3A4 inhibitor | No | No | No | No | No | No | No | Yes | No | No | No | Yes |
| Log *K*_p_ (skin permeation) | -4.48 cm/s | -3.75 cm/s | -4.16 cm/s | -4.78 cm/s | -2.20 cm/s | -6.33 cm/s | -0.30 cm/s | -5.46 cm/s | -2.74 cm/s | -5.29 cm/s | -5.48 cm/s | -6.85 cm/s |
|  |  |  |  |  |  |  |  |  |  |  |  |  |
| Druglikeness |  |  |  |  |  |  |  |  |  |  |  |  |
| Lipinski | Yes; 1 | Yes; 0 | Yes; 1 | Yes; 0 | Yes; 1 | Yes; 0 | No; 2 | Yes; 0 | Yes; 1 | Yes; 0 | Yes; 0 | Yes; 0 |
| Bioavailability Score | 0.55 | 0.55 | 0.55 | 0.55 | 0.55 | 0.55 | 0.56 | 0.55 | 0.55 | 0.55 | 0.55 | 0.55 |
| Medicinal Chemistry | 4.67 | 4.21 | 5.53 | 1.44 | 6.30 | 2.62 | 7.27 | 2.59 | 6.21 | 4.29 | 4.48 | 3.62 |
| Caco_2_ permeability | 1.41 | 1.659 | 1.394 | 1.43 | 1.201 | 1.207 | 0.494 | 1.108 | 1.213 | 1.479 | 1.508 | 1.128 |
| Intestinal absorption (human) | 95.051 | 96.855 | 94.515 | 95.835 | 94.464 | 95.245 | 76.987 | 91.51 | 94.97 | 94.296 | 93.997 | 74.212 |
| Skin Permeability | -2.244 | -2.183 | -1.833 | -2.103 | -2.783 | -2.731 | -2.735 | -2.802 | -2.783 | -1.923 | -2.087 | -2.691 |
| VDss (human) | 0.738 | 0.335 | 0.751 | -0.046 | 0.193 | -0.51 | -1.215 | 0.138 | 0.178 | 0.42 | 0.479 | 0.431 |
| Fraction unbound (human) | 0.123 | 0.188 | 0.157 | 0.078 | 0 | 0 | 0.13 | 0.078 | 0 | 0.28 | 0.347 | 0.167 |
| BBB permeability (log BB) | 0.808 | 0.514 | 0.818 | 0.282 | 0.781 | -0.813 | -1.557 | -0.142 | 0.771 | 0.596 | 0.577 | -0.472 |
| CNS permeability (log PS) | -1.834 | -2.184 | -1.759 | -1.378 | -1.705 | -2.947 | -2.942 | -2.215 | -1.652 | -2.151 | -2.662 | -3.413 |
| Total Clearance (log ml/min/kg) | -0.144 | 1.174 | 0.973 | 0.727 | 0.628 | 0.694 | 1.781 | 0.221 | 0.618 | 1.085 | 1.077 | 0.691 |
| Renal OCT2 substrate | No | Yes | No | No | No | No | No | No | No | No | No | No |
| AMES toxicity | No | No | No | No | No | No | No | Yes | No | No | No | No |
| Max. tolerated dose (human) (log mg/kg/day) | -0.182 | -0.096 | -0.142 | 1.182 | -0.621 | 1.033 | 0.336 | -0.374 | -0.664 | 0.343 | 0.445 | 0.113 |
| hERG I inhibitor | No | No | No | No | No | No | No | No | No | No | No | No |
| hERG II inhibitor | No | No | No | No | Yes | No | No | No | Yes | No | No | No |
| Oral Rat Acute Toxicity (LD50) (mol/kg) | 1.981 | 1.643 | 1.552 | 1.732 | 2.552 | 2.215 | 2.411 | 2.183 | 2.54 | 1.918 | 1.789 | 1.99 |
| Oral Rat Chronic Toxicity (LOAEL) (log mg/kg_bw/day) | 1.204 | 2.021 | 1.334 | 2.394 | 0.855 | 1.538 | 0.217 | 1.566 | 0.872 | 1.475 | 1.212 | 1.109 |
| Hepatotoxicity | No | No | No | No | No | No | No | No | No | No | No | Yes |
| Skin Sensitisation | Yes | Yes | No | Yes | No | No | No | No | No | Yes | Yes | No |
| *T.Pyriformis* toxicity(log ug/L) | 1.084 | 1.752 | 1.431 | 1.361 | 0.43 | 0.51 | 0.285 | 0.869 | 0.433 | 1.49 | 1.254 | 0.339 |
| Minnow toxicity(log mM) | -0.088 | 0.399 | 0.452 | 0.834 | -1.802 | -1.671 | -5.812 | 0.453 | -1.675 | 0.743 | 0.906 | -0.938 |

n-octanol/water coefficient (log Po/w) is a key physicochemical parameter for drug discovery, design and development. Solubility (L: low, M: moderate, H: High, I: Intermediate). n = 3.
